# The Use of Biofluid Markers to Evaluate the Consequences of Sport-Related Subconcussive Head Impact Exposure: A Scoping Review

**DOI:** 10.1186/s40798-023-00665-6

**Published:** 2024-01-25

**Authors:** Liivia-Mari Lember, Michail Ntikas, Stefania Mondello, Lindsay Wilson, Thomas G. Di Virgilio, Angus M. Hunter, Firas Kobeissy, Yehia Mechref, David I. Donaldson, Magdalena Ietswaart

**Affiliations:** 1 Department of Psychology, Faculty of Natural Sciences, University of Stirling, Stirling, UK; 2 The School of Psychology, University of Aberdeen, Aberdeen, UK; 3 Biomedical and Dental Sciences and Morphofunctional Imaging, Faculty of Medicine and Surgery, University of Messina, Messina, Italy; 4 Physiology Exercise and Nutrition Research Group, Faculty of Health Sciences and Sport, University of Stirling, Stirling, UK; 5 Department of Sports Science, Nottingham Trent University, Nottingham, UK; 6 Center for Neurotrauma, Department of Neurobiology and Neuroscience Institute, Morehouse School of Medicine (MSM), Multiomics & Biomarkers, Atlanta, GA 30310 USA; 7 Department of Chemistry and Biochemistry, Texas Tech University, Lubbock, TX USA; 8 School of Psychology and Neuroscience, University of St Andrews, St. Andrews, UK

**Keywords:** Traumatic brain injury, Diagnostics, Neurodegenerative disease, Fluid biomarkers, Contact sport, Heading

## Abstract

**Background:**

Amidst growing concern about the safety of sport-related repetitive subconcussive head impacts (RSHI), biofluid markers may provide sensitive, informative, and practical assessment of the effects of RSHI exposure.

**Objective:**

This scoping review aimed to systematically examine the extent, nature, and quality of available evidence from studies investigating the effects of RSHI on biofluid markers, to identify gaps and to formulate guidelines to inform future research.

**Methods:**

PRISMA extension for Scoping Reviews guidelines were adhered to. The protocol was pre-registered through publication. MEDLINE, Scopus, SPORTDiscus, CINAHL, PsycINFO, Cochrane Library, OpenGrey, and two clinical trial registries were searched (until March 30, 2022) using descriptors for subconcussive head impacts, biomarkers, and contact sports. Included studies were assessed for risk of bias and quality.

**Results:**

Seventy-nine research publications were included in the review. Forty-nine studies assessed the acute effects, 23 semi-acute and 26 long-term effects of RSHI exposure. The most studied sports were American football, boxing, and soccer, and the most investigated markers were (in descending order): S100 calcium-binding protein beta (S100B), tau, neurofilament light (NfL), glial fibrillary acidic protein (GFAP), neuron-specific enolase (NSE), brain-derived neurotrophic factor (BDNF), phosphorylated tau (*p*-tau), ubiquitin C-terminal hydrolase L1 (UCH-L1), and hormones. High or moderate bias was found in most studies, and marker-specific conclusions were subject to heterogeneous and limited evidence. Although the evidence is weak, some biofluid markers—such as NfL—appeared to show promise. More markedly, S100B was found to be problematic when evaluating the effects of RSHI in sport.

**Conclusion:**

Considering the limitations of the evidence base revealed by this first review dedicated to systematically scoping the evidence of biofluid marker levels following RSHI exposure, the field is evidently still in its infancy. As a result, any recommendation and application is premature. Although some markers show promise for the assessment of brain health following RSHI exposure, future large standardized and better-controlled studies are needed to determine biofluid markers’ utility.

**Supplementary Information:**

The online version contains supplementary material available at 10.1186/s40798-023-00665-6.

## Key Points

- This is the first systematically conducted review focused on scoping biofluid markers in sport-related repetitive subconcussive head impact (RSHI) research, identifying a significant body of evidence not previously featured in relevant systematic reviews.
- The scoping review identified critical limitations of the current research in the field, including lack of impact monitoring and failure to sufficiently control for confounding variables such as concussion history and the effect of exercise. Findings reveal that the current evidence base is largely heterogeneous, limiting any firm conclusions at this stage.
- Despite limited and heterogeneous evidence, some markers appeared to show promise in detecting the effects of subconcussive head impacts.

**Supplementary Information:**

The online version contains supplementary material available at 10.1186/s40798-023-00665-6.

## Background

A growing body of evidence demonstrates a link between participation in sports that have a high incidence of (head) impacts and long-term neurological impairment and/or neurodegenerative diseases [1–9]. It is thought that as many as 10–20% of professional boxers suffer from chronic neuropsychiatric disorders [10–12]. Furthermore, an increased incidence of neurodegenerative diseases has been observed in ex-professional soccer [2, 7], rugby [9], and National Football League players [8] compared to the general population. Traumatic brain injury (TBI) is increasingly recognized as a risk factor for later developing neurodegenerative processes and diseases [6, 13, 14]. Evidence for a link between contact sport and chronic traumatic encephalopathy (CTE) is also strengthening [1, 15, 16]. Interestingly, years of contact sport exposure has been associated with CTE pathology regardless of the number of symptomatic TBIs such as sport-concussion [15]. In fact, estimated total cumulative exposure to repetitive head impacts has been found to be a stronger predictor of later cognitive and neurobehavioral impairment than concussion history in American football players [17]. The recently emerging picture is that routine exposure to repetitive head impacts may pose a significant risk to brain health, quite separate from (accidental) impact exposure resulting in TBI (e.g., sport-concussion). Routine impacts in sport are either direct hits to the head (such as soccer headers) or blows to the body (e.g., full-body collisions between players, which are frequent in sports such as rugby, ice hockey, and American football). In recent years, a prominent public debate has started regarding the safety of routine head impacts in contact sports [18]. Such impacts are termed repetitive subconcussive head impacts (RSHI) and characterize the routine and repeated head impacts athletes sustain during contact sport participation that do not result in overt concussion symptoms [19]. Different lines of enquiry are based on the idea that RSHI can trigger subclinical pathology and a complex cascade of molecular alterations [20].

There are two main reasons why the relationship between RSHI and pathological processes has been seemingly neglected until recently. One is that TBI, such as sport-concussion, is common in those sports that also expose participants to RSHI, meaning that the two sources of impact in sport are often conflated (a study challenge addressed in this review). Inevitably, the symptomatic source of impact (concussion) receives more attention with regard to consequences to brain health than the routine and ‘normalized’ source of impact that does not result in evident injury symptoms. The latter issue, lack of evident symptoms, is also the second main reason why RSHI may be under-researched. Until recently, measures to assess brain health consequences of RSHI appeared to lack sensitivity [18]. While it is unclear what risk RSHI poses to brain health, there is a need for measures that are (1) sensitive, (2) specific, and (3) informative in revealing the effects of RSHI on the brain. Biofluid markers of brain injury have developed in recent years, and their use to detect RSHI-induced brain changes is an emerging field of research [21, 22]. Biofluid markers of brain injury can potentially be an efficient and practical method for providing information about routine sport-related RSHI exposure effects on brain health.

Multiple international studies have provided evidence that biofluid markers are associated with brain damage after TBI and have the potential as an objective tool for diagnosis and outcome prediction [23–28]. The implementation of ultrasensitive assays has opened up possibilities to accurately and noninvasively detect subtle structural damage, and more recently, it has been shown that biomarkers can also be used to monitor progressive alterations in the brain, years after TBI [29]. Furthermore, biomarker levels indicate axonal, neuronal and astroglial changes and injury, and their combination can reflect (and provide information on) molecular and cellular responses and underlying pathological mechanisms triggered by head trauma [30–32]. As such, there is evident potential for the use of biomarkers to identify subtle RSHI-induced brain changes that may be undetectable based on clinical criteria or imaging assessment. Assessing the functionality of different biomarkers and their ability to detect the effects of RSHI on the brain is thus of great importance: these markers may aid understanding of RSHI-induced brain pathology and give an insight into the link between acute brain changes and chronic neurodegenerative sequelae. The biofluid marker evidence base specific to the effects of RSHI has, however, not yet been reviewed. The evaluation of the biomarkers in RSHI is complicated by methodological and analytical variability among studies, including research designs, populations, settings, sampling times, analytical approaches, sources, and outcomes being assessed.

Therefore, we conducted a scoping review to identify and comprehensively map the number, features, and quality of studies that have explored the effects of RSHI on biomarker levels. Besides providing an overview of the existing and emerging evidence, we focused on defining methodological problems and identifying potential solutions and research gaps to inform and guide the design and analysis of future studies and research.

## Methods

### Protocol and Registration

This scoping review adheres to the Preferred Reporting Items for Systematic Reviews and Meta-Analyses Extension for Scoping Reviews (PRISMA-ScR) [33] guidelines. The review protocol has been published in BMJ Open [19].

#### Information Sources

The following seven electronic databases were searched from inception until March 2022: Cochrane Library, MEDLINE (EBSCO host), Scopus, SPORTDiscus, CINAHL Complete, PsycINFO, and OpenGrey. The following clinical trial registration platforms were also searched for relevant protocols and corresponding full-text publications: ClinicalTrials.gov and WHO International Clinical Trials Registry Platform. Key descriptors that included terms for subconcussive head impacts, biomarker, and contact sport (see Additional file 1: Table S1 for examples) were used for the search. The full search strategies are available in Additional file 1: Table S1. Reference lists of the included studies were also screened to identify additional records.

### Study Selection and Eligibility Criteria

We used the web-based systematic review software Covidence (Covidence, Veritas Health Innovation, Melbourne, Australia) (available at www.covidence.org) for the selection process. After the removal of duplicates, two reviewers (L-ML and MN) independently screened the titles and abstracts against the predetermined eligibility criteria, followed by full-text review of retained articles. Any disputes between reviewers were resolved through discussion and if necessary, by a third reviewer (SM).

We included studies that investigated biofluid markers, including brain injury markers such as S100 calcium-binding protein beta (S100B), ubiquitin C-terminal hydrolase L1 (UCH-L1), glial fibrillary acidic protein (GFAP), neurofilament light (NfL), tau, and microRNAs (miRNAs), cytokines, chemokines, and hormones, in blood (serum or plasma), cerebrospinal fluid (CSF), saliva or urine in athletes who were acutely or chronically exposed to sport-related RSHI. We excluded studies assessing biomarker concentrations following solely sports-related concussion or traumatic brain injury. Studies that assessed the effects of repetitive head impacts (both RSHI and concussions) were included. However, if those studies did not separate concussions from RSHI through either (1) exclusion of concussion cases or (2) analysis (covariate), then this was reflected in the bias and quality rating conducted as part of this review. Post-mortem and non-human examinations were also excluded. No restrictions were placed on methodological standards, analytical platforms, study design, and sample size. Studies were included regardless of geographic location and date of publication. We considered reports in the English, French, German, and Italian languages. Detailed inclusion criteria including the Population, Exposure, Comparator, Outcomes, and Study Design (PECOS) framework applied in this scoping review are available in Additional file 1: Table S2. A list of excluded articles with reasons for exclusion (e.g., duplication or redundant publication) during full-text screening is provided in Additional file 1(Table S3).

### Data Extraction and Results Categorization

Data were recorded independently by two reviewers using a standardized and piloted data collection form. Disagreements were discussed until consensus was reached, and, if necessary, a third reviewer was consulted for arbitration. Information about the study design, aim(s), population, RSHI definition, exposure to RSHI, and biofluid marker characteristics (including sampling time, source, analytical platform, and concentrations) were extracted. Studies were classified as either laboratory- or field-based, depending on whether the RSHI occurred in a controlled environment or in the field (such as during training, games, or matches). Further, studies were categorized as acute, semi-acute, or chronic. Studies were considered acute if changes in biomarker concentrations were assessed immediately following RSHI exposure (< 2 weeks) and semi-acute if changes were assessed following an extended rest period from RSHI (e.g., ≥ 2 weeks), or if the effects of accumulation of RSHI were assessed over a season. Studies that investigated the relationship between the history of contact sport participation (years of participation, total number of games or competitions in lifetime) and biofluid marker concentrations were considered to assess the chronic effects.

### Risk of Bias and Quality Assessment of Included Studies

A modified version of the risk of bias in non-randomized studies of interventions (ROBINS-I) tool [34] was used to assess the methodological quality of all primary research publications by evaluating four domains: (1) confounding variables, (2) missing data, (3) measurement of outcomes, and (4) selection of reported results. Confounding variables were considered factors, other than RSHI, that could influence the concentration of the biofluid markers, such as exercise, history of concussion, peripheral injuries, neurological diseases, and so on.

In addition, to increase rigor and determine the quality of study reporting, a modified version of the Subconcussion-Specific Tool (SST) was utilized to assess the quality of the included studies [22, 35]. Each study was assessed for the following six criteria: (1) Was there an attempt to define the term ‘subconcussion’? (2) Was the number or magnitude of impacts reported (or used in the analysis)? (3) Were participants who sustained a concussion during the study controlled for or excluded from analyses? (4) Were participants with a history of concussion controlled for or excluded from the analyses? (5) Was the control group matched on two or more variables (e.g., history of concussion, sex, age, etc.)? (6) Did the study analyze sex differences, or acknowledge limitations associated with sampling only males or females? Studies were classified as category A, B, or C (i.e., high, medium, and low quality) depending on how many criteria were fulfilled. Category A studies met five or more criteria, B category studies three or four criteria, and C two or less. Question three was not relevant to cross-sectional studies assessing the chronic effects of RSHI in retired athletes and as such, for the purpose of classification, this criterion was considered achieved for these studies. Two of the review authors (L-ML, MN) independently assessed the studies for the risk of bias and quality. Disagreements were resolved through consensus and if necessary, arbitration by a third reviewer was sought.

### Synthesis and Reporting of the Results

The search results are reported in a flow diagram detailing the review decision process. The synthesis of results includes a narrative and quantitative summary in text and the main characteristics of the included studies are presented in tables. The results are categorized and presented according to a priori defined categories and inductively developed categories (i.e., biofluid markers, the timing of sampling [acute, semi-acute or chronic], setting [laboratory or field], and sample source [blood, CSF or saliva]). Risk of bias graphs were generated using robvis web-based software [36] (available at https://mcguinlu.shinyapps.io/robvis/).

## Results

### Description of Studies

**Fig. 1:**
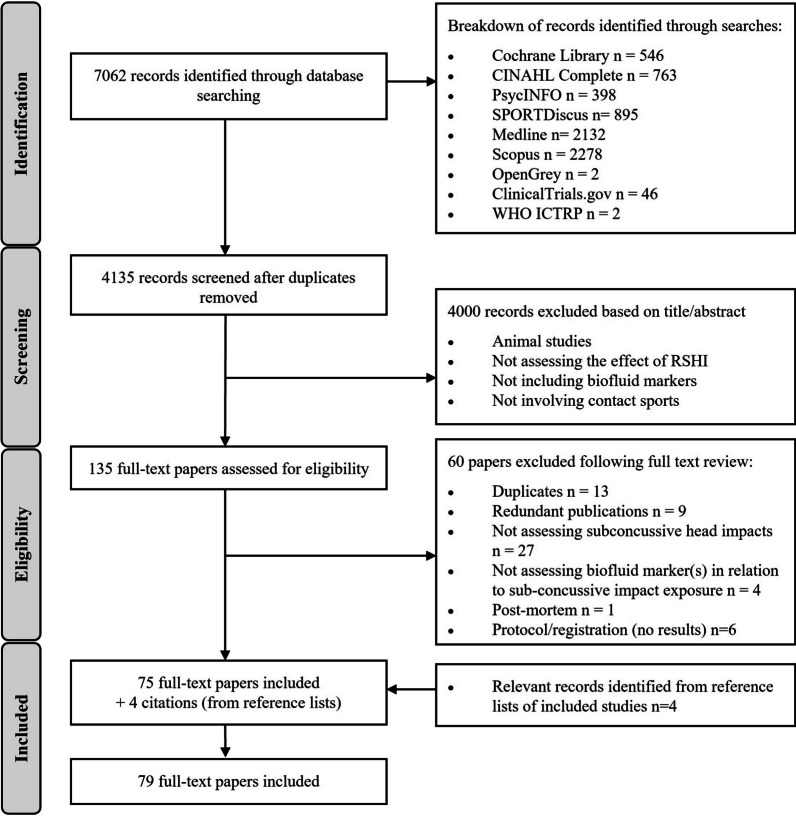
PRISMA flow diagram

Our searches retrieved 7062 records from which 4135 titles and abstracts were screened following the removal of duplicates. One hundred and thirty-five full-text articles were assessed for eligibility and 79 articles were included in the review (see Fig. 1; detailed information about the studies can be found in Additional file 1: Table S4). Inter-rater reliability in the study identification process was substantial for title/abstract screening and moderate for full-text review (*κ *= 0.71 and 0.60, respectively).

**Fig. 2:**
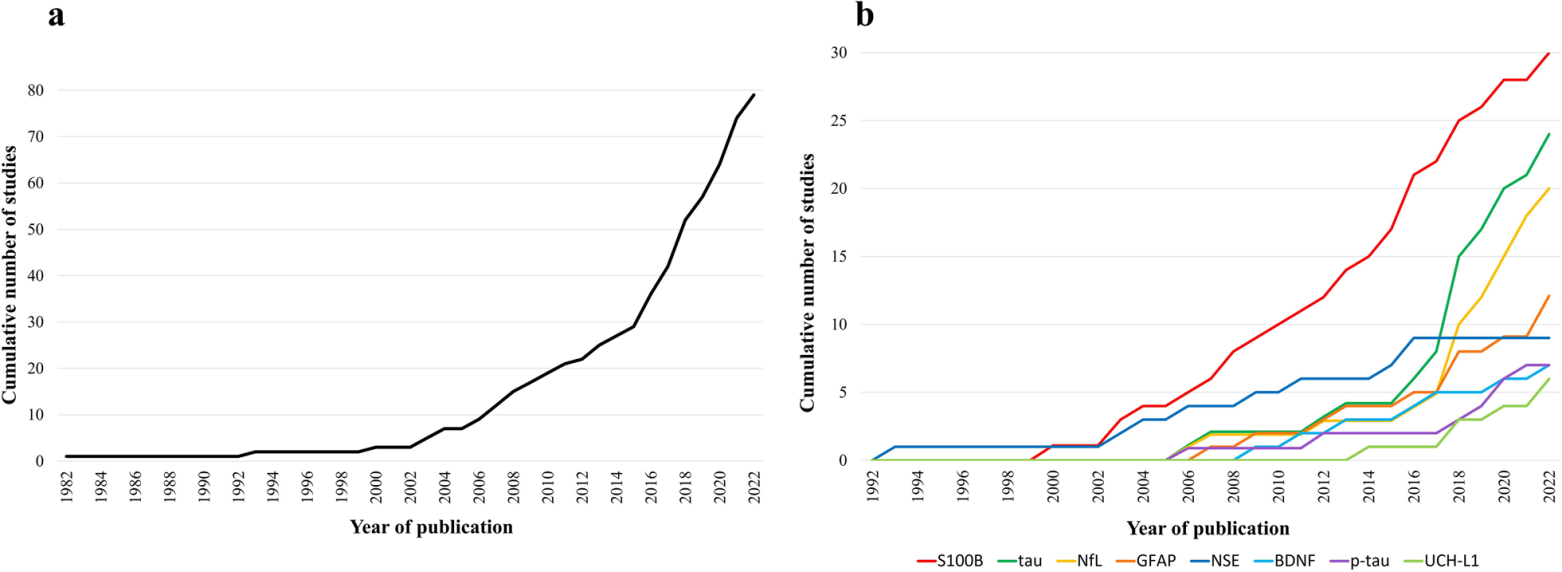
**a** Temporal trend of all the studies; **b** Temporal trend by biomarker

The earliest identified record was published in 1982 [37] with the number of studies increasing remarkably in the last decade (Fig. 2).

The majority (~ 85%) of the studies employed an observational design with 44 cohort, 19 cross-sectional, and four case–control studies. Only 11 studies (~ 14%) employed an experimental design with seven of them being randomized. We identified just one case report relevant to this scoping review.

Forty-nine studies assessed markers acutely, 23 in the semi-acute-phase and 26 investigated long-term effects. Eighteen studies assessed a mix of the acute, semi-acute, or chronic effects of RSHI exposure.

Further, 13 studies (~ 16%) were laboratory-based and 45 were field-based (~ 57%). A case report and the majority of the chronic studies were not considered laboratory- or field-based and were categorized as ‘other’ (22 out of 79, ~ 28% of studies).

Most studies (~ 53%) have been conducted on male athletes (42 out of 79). There were only two studies conducted on female participants [38, 39] and 20 included a mixed population. Sex was not specified in 15 studies. Only three studies included exclusively individuals younger than 18 years (age range ~ 13–17) [40–42]. Fifty-two studies employed either a control condition or had a control cohort.

Most studied markers were: S100B (30 studies), tau (24 studies, including 4 studies assessing tau in extracellular vesicles [EVs]), NfL (20 studies), GFAP (14 studies), NSE (9 studies), BDNF (7 studies), phosphorylated tau (p-tau) (7 studies), and UCH-L1 (6 studies) (see Table 1). Further, nine studies assessed the hormonal response to RSHI (~ 10%). All other biofluid markers had fewer than five research publications available per marker; information about all markers is provided in Additional file 1: Table S4. The vast majority of the samples were venous i.e. from serum and/or plasma (72 studies), while some studies sampled from cerebrospinal fluid (six studies) [43–48], or from saliva (five studies) [49–53].

**Table 1:** Biomarker specific tables for selected biomarkers: S100B, tau, neurofilament light (NfL), glial fibrillary acidic protein (GFAP), neuron-specific enolase (NSE), brain-derived neurotrophic factor (BDNF), and ubiquitin C-terminal hydrolase—L1 (UCH-L1)

Reference	Study type	Design	Setting	Sport	Athlete group	Control group	Exposure	Source	Sample times	Findings	Bias	QA
*S100B*												
Arslan et al. [54]	Acute	Cohort study	Field	Wrestling	15 male Greco-Roman wrestlers, median age (range) 19.0 (19–30); 16 male Free style wrestlers, aged 20.0 (19–26)	N/A	Wrestling competition (3 × 2 min)	Serum	Before and 20 min post	Significant findings	Serious	C (1)
Asken et al. [55]	Chronic	Observational cohort study	Other	Soccer, diving, wrestling, ice hockey, Am. football	415 (256 M, 159 F) collegiate athletes, aged 19.0 ± 1.2	N/A	Cumulative exposure to collision sports in years (and modified CHII)	Serum	Off-season	No significant findings	Moderate	B (4)
Bouvier et al. [56]	Acute	Prospective cohort study	Field	Rugby	39 professional rugby players, aged 28.6 ± 3.98 (27 non-concussed, 5 concussed)	N/A	Rugby match (collisions)	Serum	3 basal levels during the season (> 48 h from competition) and within 2 and 36 h after a match	Significant findings	Moderate	B (4)
DiBattista et al. [57]	Chronic	Cross-sectional study	Other	Ice hockey, football, rugby, lacrosse	41 (39 M, 2 F) collision sport athletes; aged (including all participants): M (*n *= 60) 19.5 ± 2.0, F (*n *= 27) 19.5 ± 1.8	46 (21 M, 25 F) non collision sport athletes (inadvertent contact: soccer, basketball)	Collision sport participation	Plasma	Before the start of varsity season	No significant findings	Moderate	B (4)
Dorminy et al. [58]	Acute	Randomized controlled trial	Laboratory	Soccer	16 (10 M, 6 F), aged 20.4 ± 0.2	N/A	5 linear standing headers	Serum	Before and 1–1.5 h post	No significant findings	Serious	B (4)
Graham et al. [59]	Acute	Retrospective cohort study	Field	Boxing	8 male amateur boxers, aged 17.6 ± 5.3 (PTH – punches to the head and body)	8 male amateur boxers, aged 19.1 ± 3.2 (PTB – punches to the body)	5 × 2-min boxing rounds	Serum	1 h before and after 5 min of cessation	Significant findings	Moderate	C (2)
Graham et al. [60]	Acute	Cohort study	Field	Karate	12 males, aged 30.4 ± 6.7 (KTH – kicks to the head and body)	12 males, aged 28.2 ± 6.5 (KTB – kicks to the body)	4 × 3-min karate round	Serum	Before and immediately after	Significant findings	Moderate	C (1)
Hoffman et al. [61]	Acute	Cohort study	Field	Am. football	15 Israel national football team players, aged 26.2 ± 5.3	N/A	Am. football match	Serum	1 week before, immediately (< 30 min) and 24 h post	No significant findings	Moderate	C (2)
Huibregtse et al. [62]	Acute	Randomized controlled trial	Laboratory	Soccer	37 (19 M, 18 F), median age (IQR) 21 (19–22)	31 (14 M, 17 F), median age (IQR) 21 (20–22)	10 linear headers; controls: 10 kicks	Plasma	Before and 0, 2 and 24 h post	No significant findings	Low	A (5)
Kawata [63]	Semi-acute	Prospective longitudinal cohort study	Field	Am. football	22 male Division I collegiate footballers, aged 20.6 ± 1.5	N/A	Am. football season	Plasma	Pre- and post-season, and before and after 5 practices (1 non-contact, 4 full contact)	No significant findings	Moderate	A (6)
Kawata et al. [64]	Acute	Prospective longitudinal cohort study	Field	Am. football	22 male Division I collegiate footballers, aged 20.6 ± 1.5	N/A	Pre-season Am. football practices	Plasma	Baseline, before and after 5 pre-season practices (1 non-contact, 4 full contact)	Significant findings (both in contact and no contact). Impacts correlated with the increase	Moderate	A (5)
Marchi et al. [65]	Acute and semi-acute	Cohort study	Field	Am. football	Acute: 27 collegiate players, aged ~ 21 Semi-acute: 10 collegiate players	N/A	Am. football matches and season	Serum	Acute: baseline (prior to any football related activity), 24 h before, 1 and 24 h post Semi-acute: pre- and post-season	Acute: significant findings in players that had frequent head impacts. Increase correlated with head impact index Semi-acute: anti-S100b Ab increased in 5 out of 10 players	Serious	B (4)
Mussack et al. [40]	Acute	Non-randomized experimental study	Laboratory	Soccer	61 male amateur players, median age (IQR) 15.3 (14.8—16.4)	58 male amateur players, median age (IQR) 15.9 (15.0—16.8); 81 mTBI controls: 20 CCT + 41.8 (32.3—61.1), 61 CCT- 37.1 (27.6—53.5)	Controlled soccer heading aimed at the forehead performed for a median: 55 min; control: 61 min of exercise	Serum	Baseline and 1 and 6 h post	No significant findings. Significant findings in the mTBI group	Moderate	B (3)
Neselius et al. [46]	Acute and semi-acute	Prospective cohort study	Field	Boxing	30 (28 M, 2 F) Olympic boxers, mean age (range) 22 (17–34)	25 (20 M, 5 F) healthy controls, mean age (range) 22 (17–30)	Boxing bout	CSF	1–6 days post and after ≥ 14 days rest	Acute: significant findings Semi-acute: no significant findings	Moderate	C (2)
Neselius et al. [66]	Acute and semi-acute	Prospective cohort study	Field	Boxing	30 (28 M, 2 F) amateur boxers (competing at elite level), mean age (range) 22 (17–34)	25 (20 M, 5 F) healthy controls, mean age (range) 22 (17–30)	Boxing	Serum	1–6 days post and after ≥ 14 days rest	No significant findings	Serious	C (2)
O'Connell et al. [67]	Acute and semi-acute	Prospective longitudinal cohort study	Field	Rugby	38 professional male rugby players, aged 26.6 ± 4.4	15 rowers, median age (IQR) 22.0 (20.0—24.0)	Rugby training and games	Serum	Pre- and post-season, ≤ 2 h post-games Controls: pre- and post-80 min of training	Acute: significant findings Semi-acute: no significant findings	Moderate	C (1)
O'Keeffe et al. [68]	Acute and semi-acute	Cohort study	Field	Rugby	8 rugby university team players, mean age (range) 22.1 (18–23); 11 male rugby school team players, mean age 17.4	27 non-contact sport athletes, median age (range) 28 (18–36); 26 healthy non-athlete controls, median age (range) 30 (18–40)	Rugby match (university team) and season (school and university team)	Plasma	University team: pre-season, ≤ 2 h post-match, 2 months post-season School team: pre- and post-season	Acute: significant findings Semi-acute: significant findings (decrease)	Moderate	B (3)
Otto et al. [69]	Acute	Cohort study	Field, laboratory	Boxing, soccer	25 male amateur boxers: competitive fights *n *= 10, sparring fights *n *= 15 (13 with head protector), aged 17–40; heading: 12 sportsmen, aged 20–52	35 male runners (sprinters, 10 and 25 km), aged 20–52. 12 male cyclists, aged 23–52	(1) 5 × 2-min competitive boxing rounds (2) 3 or 5 × 2-min sparring fights (3) 20 standing soccer headers (ball dropped from 7.5 m)	Serum	Before and ≤ 15 min post	Mixed findings (boxing: significant; soccer: not significant)	Serious	C (1)
Puvenna et al. [70]	Acute	Cohort study	Field	Am. football	15 athletes	406 positive controls with mTBI and 465 negative controls	2 Am. football games	Serum	Baseline (day before) and post (< 1 h) (positive controls: < 6 h of injury)	Significant findings	Serious	B (3)
Rogatzki et al. [71]	Acute	Cohort study	Field	Am. football	17 male Division III collegiate footballers, aged 19.5 ± 0.9	N/A	Am. football game	Serum	2 days before and 1 h post	Significant findings	Moderate	C (2)
Rogatzki et al. [72]	Acute and semi-acute	Cohort study	Field	Am. football	16 male Division III collegiate footballers, age range 18 to 22	32 controls, age range 18–22 [control groups: resistance exercise *n *= 18 (10 M, 8 F); treadmill running *n *= 8 (5 M, 3 F); treadmill walking *n *= 6 (3 M, 3 F)]	Am. football game	Serum	Baseline (prior to training camp); before (day before, ≤ 30 min post-practice) and ≤ 30 min post-4 games Controls: immediately before and ≤ 30 min post	Acute: significant findings for experimental and 2 control groups Semi-acute: no significant findings Number of hits and plays correlated with S100B	Moderate	B (3)
Soriano et al. [52]	Semi-acute	Cohort study	Field	Am. football	33 male collegiate players, aged 19.3 ± 1.4	N/A	Am. football season (games and training)	Serum	Mid-season, post-season and off-season (after a rest period)	No significant findings	Serious	B (4)
Stålnacke and Sojka [73]	Acute	Randomized controlled trial	Laboratory	Soccer	10 male amateur players, aged 22 ± 8 (age for entire sample *n *= 19)	9 male amateur players	5 headers in 15–20 min (ball dropped from 18 m, velocity 63.6 km/h)	Serum	Before and 0.5, 2 and 4 h post	No significant findings	Moderate	B (3)
Stålnacke et al. [74]	Acute	Cohort study	Field	Ice hockey	26 male elite ice hockey players, aged 28 ± 4	18 elite basketball players, aged 25 ± 4	Ice hockey game (body checkings, falls, collisions, boardings); basketball game (jumps, collisions, falls)	Serum	1–2 h before and ≤ 1 h post	Significant findings in all conditions	Serious	C (2)
Stålnacke et al. [75]	Acute	Cohort study	Field	Soccer	28 male elite players, aged 26 ± 5	N/A	Headers, jumps, falls and collision during a competitive soccer game	Serum	1–5 h before and immediately post	Significant findings	Moderate	B (3)
Stålnacke et al. [38]	Acute	Cohort study	Field	Soccer	44 female elite players, aged 23 ± 3	N/A	Headers, jumps, falls and collisions during a competitive soccer game	Serum	Before and immediately post	Significant findings (changes correlated with headers and jumps, collisions, and falls)	Moderate	B (3)
Straume-Naesheim et al. [76]	Acute	Prospective cohort study	Field	Soccer	Professional soccer players: heading exercise *n *= 46, mean age 26.1; head impacts during a match *n *= 69, 28.1	Professional soccer players: high intensity exercise *n *= 48, 26.1; match control *n *= 56, 26.2	Heading exercise (90 min), head impacts (some concussive) during match play Controls: 90 min exercise, match w/o head trauma	Serum	Baseline, 1 and 12 h post	Significant findings in all conditions	Serious	B (3)
Zetterberg et al. [48]	Acute	Non-randomized experimental study	Laboratory	Soccer	23 male amateur soccer players, median age (range): 10 headers *n *= 10, 26 (19–32); 20 headers *n *= 13, 23 (20–28)	9 male non athletes, median age (range) 24 (22–27)	10 or 20 standing headers from a corner kick (kicked from 30 m)	CSF and serum	7–10 days post	No significant findings	Serious	B (3)
Zetterberg et al. [77]	Chronic	Observational case–control study	Other	Boxing	44 male amateur boxers, median age (range) 19 (17–28)	23 healthy males w/o contact sport history, median age (range) 28 (19–50)	Boxing participation (boxing debut, boxing duration in yr, number of bouts)	Serum	After a 2-month period of nonparticipation in boxing	No significant findings	Moderate	C (1)
Zonner et al. [41]	Acute and semi-acute	Longitudinal prospective cohort study	Field	Am. football	15 high school footballers, aged 16.4 ± 0.5	N/A	Am. football games and season	Serum	Semi-acute: pre- and post-season; acute: 4–5 h before and ≤ 1 h after 5 games	Acute: significant findings Semi-acute: no significant findings	Low	A (5)
*Tau*												
Alosco et al. [78]	Chronic	Cross-sectional study	Other	Am. football	96 male symptomatic former NFL players, aged 55.2 ± 7.9	25 asymptomatic controls w/o contact sport history, aged 57.0 ± 6.6	Am. football (NFL) career	Plasma	N/A	Mixed	Moderate	C (2)
Alosco et al. [43]	Chronic	Cross-sectional study	Other	Am. football	68 male symptomatic former NFL players, aged 54.4 ± 8.0	21 asymptomatic controls w/o contact sport history, aged 57.6 ± 7.1	Am. football (NFL) participation (CHII)	CSF	N/A	Tau: mixed P-tau: no significant findings	Moderate	C (2)
Asken et al. [55]	Chronic	Observational cohort study	Other	Soccer, diving, wrestling, ice hockey, Am. football	415 (256 M, 159 F) collegiate athletes, aged 19.0 ± 1.2	N/A	Cumulative exposure to collision sports in yr (and modified CHII)	Serum	Off-season	No significant findings	Moderate	B (4)
Bernick et al. [79]	Acute, semi-acute, and chronic	Longitudinal cohort study	Other	Boxing, MMA	52 (50 M, 2 F) retired professional boxers, aged 48.0 ± 10.3 117 (110 M, 7 F) active professional boxers, aged 30.4 ± 6.9 169 (152 M, 17 F) active professional MMA fighters, aged 29.6 ± 4.8	79 (69 M, 10 F) controls w/o contact sport history, aged 30.8 ± 10.0	Fights and sparring (martial arts or boxing)	Plasma	Baseline and ≥ 2 measurements over 1.6 years (average) (range 1–5 years); active fighters: ≥ 45 days from a sanctioned fight	Acute and chronic: no significant findings Semi-acute: significant findings	Serious	C (1)
Di Battista et al. [80]	Chronic	Cross-sectional study	Other	Ice hockey, football, rugby, lacrosse	41 (39 M, 2 F) collision sport athletes; aged (including all participants): M (*n *= 60) 19.5 ± 2.0, F (*n *= 27) 19.5 ± 1.8	46 (21 M, 25 F) non collision sport athletes (inadvertent contact: soccer, basketball)	Collision sport participation	Plasma	Before the start of varsity season	Significant findings	Moderate	B (4)
Hoffman et al. [61]	Acute	Cohort study	Field	Am. Football	15 Israel national football team players, aged 26.2 ± 5.3 (range 18–35)	N/A	Am. football match	Serum	1 week before, immediately (< 30 min) and 24 h post	No significant findings	Moderate	C (2)
Joseph et al. [42]	Acute and semi-acute	Prospective observational cohort study	Field	Am. football	16 male high-school varsity footballers, aged 16.9 ± 0.2 (pre- and post-season sample *n *= 12)	N/A	Am. football games, practices and season	Serum	Semi-acute: pre- and post-season; acute: 1–2 h post	Significant findings	Moderate	B (3)
Kawata et al. [81]	Acute	Prospective longitudinal cohort study	Field	Am. football	23 male Division I collegiate footballers, aged 20.5 ± 1.3	N/A	Pre-season Am. football practices	Plasma	Pre-season baseline, immediately before and ≤ 1 h after 4 practices (1 non-contact)	Significant findings	Moderate	A (5)
Kawata et al. [82]	Semi-acute	Cohort study	Field	Ice hockey	8 male professional players (including 2 concussed athletes), aged 26.6 ± 1.6	N/A	Ice hockey season	Plasma	Pre- and post-season	No significant findings	Critical	C (1)
Major et al. [83]	Chronic	Cross-sectional study	Other	Au. football	81 (50 M, 31 F) amateur footballers (no-mTBI history *n *= 42; mTBI history *n *= 39), aged ~ 24	42 (23 M, 19 F) age-matched non-contact sport athletes	Au. football participation	Serum	Pre-season	Tau: no significant findings P-tau: no significant findings	Moderate	A (5)
Muraoka et al. [44]	Chronic	Cross-sectional study	Other	Am. football	15 male symptomatic former NFL players, aged 56.3 ± 7.3	16 asymptomatic males w/o contact sport history, aged 57.1 ± 7.0	Am. football career	CSF	N/A	Tau: no significant findings P-tau: no significant findings	Moderate	B (3)
Muraoka et al. [84]	Chronic	Cross-sectional study	Other	Am. football	27 male symptomatic former NFL players, aged 56.6 ± 7.6	25 asymptomatic males w/o contact sport history, aged 57.0 ± 6.6	Am. football career	Plasma	N/A	Tau: significant findings P-tau: significant findings	Moderate	B (3)
Neselius et al. [46]	Acute and semi-acute	Prospective cohort study	Field	Boxing	30 (28 M, 2 F) Olympic boxers, mean age (range) 22 (17–34)	25 (20 M, 5 F) healthy controls, mean age (range) 22 (17–30)	Boxing bout	CSF	1–6 days post and after ≥ 14 days rest	Tau: Acute: significant findings Semi-acute: no significant findings P-tau: no significant findings	Moderate	C (2)
Neselius et al. [66]	Acute and semi-acute	Prospective cohort study	Field	Boxing	30 (28 M, 2 F) amateur boxers (competing at elite level), mean age (range) 22 (17–34)	25 (20 M, 5 F) healthy controls, mean age (range) 22 (17–30)	Boxing bout	Plasma	1–6 days post and after ≥ 14 days rest	Acute: significant findings Semi-acute: no significant findings	Serious	C (2)
Nowak et al. [85]	Acute	Case–control study	Laboratory	Soccer	17 (6 M, 11 F) soccer players with ADHD, aged 20.2 ± 0.2; 17 (10 M, 6 F) w/o ADHD, aged 21.1 ± 0.1	17 (7 M, 10 F) soccer players with ADHD, aged 20.5 ± 0.1	10 linear headers; controls: 10 kicks	Plasma	Baseline, 2 and 24 h post	No significant findings	Low	A (5)
Oliver et al. [86]	Semi-acute	Longitudinal observational cohort study	Field	Am. football	19 (11 starters, 8 non-starters) Division I footballers, aged 20 ± 1	19 NCAA swimmers, aged 20 ± 1 (baseline sample only)	Am. football season	Plasma	T1: after 9 weeks of non-contact; T2: after training camp; T3: following pre-season camp (highest concentration of impacts); T4 through T8 mid-season, 36–48 h post-games	No significant findings	Moderate	B (3)
Oliver et al. [87]	Semi-acute	Prospective longitudinal cross-sectional study	Field	Am. football	35 (20 starters, 15 non-starters) Division III footballers, aged 21 ± 1	N/A	Am. football season	Plasma	T1: after 14-weeks of non-contact; T2: end of camp (period with most impacts); T3: 72 h post-full-contact practice, T4 and T5: ~ 36 h following a game; T6 and T7: post-season	Significant findings (decrease)	Moderate	B (3)
Sandmo et al. [88]	Acute and chronic	Prospective cohort study	Field	Soccer	Male premier league players: heading exercise group *n *= 47, head impacts during a match *n *= 35	Male premier league players: high intensity exercise *n *= 47	(1) heading exercise (90 min), (2) head impacts (some concussive) during match play	Serum	Baseline, 1 and 12 h post	No significant findings	Serious	B (2)
Soriano et al. [52]	Semi-acute	Cohort study	Field	Am. football	33 collegiate athletes, aged 19.3 ± 1.4	N/A	Am. football season (games and training)	Serum	Mid-season, post-season and off-season (after a rest period)	Not detectable	Serious	B (4)
Stern et al. [89]	Chronic	Case–control study	Other	Am. football	78 male symptomatic former NFL players, aged 54.5 ± 8.0	16 male asymptomatic non-contact sport athletes, 56.9 ± 7.2	Am. football career	Plasma	N/A	Significant findings	Moderate	C (2)
Symons et al. [53]	Chronic	Cross-sectional study	Other	Au. football	95 (69 M, aged 23.3 ± 0.4; 26 F, aged 23.2 ± 0.9) amateur players	49 (28 M, aged 22.5 ± 0.4; 21 F, aged 23.1 ± 0.8) amateur basketball, tennis, cricket, track and field athletes	Au. football participation	Serum	N/A (pre-season)	Tau: significant findings P-tau: significant findings	Moderate	B (4)
Wallace et al. [90]	Acute	Prospective controlled cohort study	Laboratory	Soccer	11 male collegiate players, aged 23.7 ± 3.9	N/A	40 headers; sham condition: contact with ball using hands, chest or thigh	Plasma	Immediately before and 1 h and 3 weeks post	No significant findings	Critical	C (2)
Zetterberg et al. [47]	Acute and chronic	Longitudinal cohort study	Field	Boxing	14 (11 M, 3 F) amateur boxers, aged 22 ± 3.8	10 male nonathletic controls, aged 30 ± 6.3	Boxing bout	CSF	7–10 days post and after 3 months of rest	Tau: Acute: significant findings Chronic: no significant findings P-tau: no significant findings	Moderate	C (0)
Zetterberg et al. [48]	Acute	Non-randomized experimental study	Laboratory	Soccer	23 male amateur soccer players, median age (range): 10 headers *n *= 10, 26 (19–32); 20 headers *n *= 13, 23 (20–28)	9 male non athletes, median age (range) 24 (22–27)	10 or 20 standing headers from a corner kick (kicked from 30 m)	CSF	7–10 days post	No significant findings	Serious	B (3)
*NfL*												
Antonio et al. [39]	Chronic	Cross-sectional study	Other	Soccer	8 female Division II soccer players, aged 22 ± 6	17 female non-contact sport athletes, aged 25 ± 8	Soccer participation	Plasma	N/A	Significant findings	Serious	C (2)
Austin et al. [91]	Acute	Randomized controlled trial	Laboratory	Soccer	36 males (12 in each heading group), aged 23.7 ± 4.8	8 males, aged 23.7 ± 4.8	10, 20 and 40 linear headers	Serum	Baseline, 6 h, 24 h, 7 days	No significant findings	Low	A (5)
Bernick et al. [79]	Acute, semi-acute, and chronic	Longitudinal cohort study	Other	Boxing, MMA	52 (50 M, 2 F) retired professional boxers, aged 48.0 ± 10.3 117 (110 M, 7 F) active professional boxers, aged 30.4 ± 6.9 169 (152 M, 17 F) active professional MMA fighters, aged 29.6 ± 4.8	79 (69 M, 10 F) controls w/o contact sport history, aged 30.8 ± 10.0	Fights and sparring	Plasma	Baseline and ≥ 2 measurements over 1.6 years (average) (range 1–5 years); active fighters: ≥ 45 days from a sanctioned fight	Acute: significant findings (boxers) Semi-acute and chronic: not significant	Serious	C (1)
Heileson et al. [92]	Semi-acute	Non-randomized controlled trial	Field	Am. football	66 male NCAA Am. football players	N/A	Am. football games and practices	Serum	Baseline: following > 14-week period of non-contact, after pre-season camp and throughout season	Significant findings	Moderate	C (2)
Joseph et al. [42]	Acute and semi-acute	Prospective observational cohort study	Field	Am. football	16 male high-school varsity footballers, aged 16.9 ± 0.2 (pre- and post-season sample *n *= 12)	N/A	Am. football games, practices and season	Serum	Semi-acute: pre- and post-season; acute: 1–2 h post	No significant findings	Moderate	B (3)
Kawata et al. [82]	Semi-acute	Cohort study	Field	Ice hockey	8 male professional players (including 2 concussed athletes), aged 26.6 ± 1.6	N/A	Ice hockey season	Plasma	Pre- and post-season	Significant findings	Critical	C (1)
Major et al. [83]	Chronic	Cross-sectional study	Other	Au. football	81 (50 M, 31 F) amateur footballers (no-mTBI history *n *= 42; mTBI history *n *= 39), aged ~ 24	42 (23 M, 19 F) age-matched non-contact sport athletes	Au. football participation	Serum	Pre-season	No significant findings	Moderate	A (5)
Neselius et al. [46]	Acute and semi-acute	Prospective cohort study	Field	Boxing	30 (28 M, 2 F) Olympic boxers, mean age (range) 22 (17–34)	25 (20 M, 5 F) healthy controls, mean age (range) 22 (17–30)	Boxing bout	CSF	1–6 days post and after ≥ 14 days rest	Significant findings	Moderate	C (2)
Nowak et al. [85]	Acute	Case–control study	Laboratory	Soccer	17 (6 M, 11 F) soccer players with ADHD, aged 20.2 ± 0.2; 17 (10 M, 6 F) w/o ADHD, aged 21.1 ± 0.1	17 (7 M, 10 F) soccer players with ADHD, aged 20.5 ± 0.1	10 linear headers; controls: 10 kicks	Plasma	Baseline, 2 and 24 h post	Significant findings (in w/o ADHD group)	Low	A (5)
Oliver et al. [93]	Semi-acute	Observational cohort study	Field	Am. football	116 Division I American footballers (baseline), aged 20 ± 1 (of whom 19 were sampled over the season; 9 non-starters, 11 starters)	19 male NCAA Division I swimmers, aged 20 ± 1 (baseline sample only)	Am. football season	Serum	T1: after 9 weeks of non-contact; T2: after training camp; T3: following pre-season camp (highest concentration of impacts); T4 through T8 mid-season, 36–48 h post-games	Significant findings	Moderate	C (2)
Oliver et al. [87]	Semi-acute	Prospective longitudinal cross-sectional study	Field	Am. football	35 (20 starters, 15 non-starters) Division III footballers, aged 21 ± 1	N/A	Am. football season	Serum	T1: after 14-weeks of non-contact; T2: end of camp (period with most impacts); T3: 72 h post-full-contact practice, T4 and T5: ~ 36 h following a game; T6 and T7: post-season	Significant findings	Moderate	B (3)
Rubin et al. [94]	Acute	Cohort study	Field	Am. football	18 Division I college footballers, median age (IQR) 20.5 (20–22)	N/A	Am. football pre-season practices	Plasma	Baseline: 2 months prior to any practices; < 1 h before and < 1 h post-practices	Significant findings	Moderate	A (6)
Sandmo et al. [88]	Acute and chronic	Prospective cohort study	Field	Soccer	Male premier league players: heading exercise group *n *= 47, head impacts during a match *n *= 35	Male premier league players: high intensity exercise *n *= 47	(1) heading exercise (90 min), (2) head impacts (some concussive) during match play	Serum	Baseline, 1 and 12 h post	No significant findings	Serious	B (2)
Shahim et al. [95]	Acute and chronic	Prospective cohort study	Field	Boxing	14 (11 M, 3 F) amateur boxers, median age (IQR) 21.5 (20–26)	14 healthy nonathletic controls, 23.5 (23–26); 12 gymnasts, 19 (18–22)	Boxing bout	Serum	7–10 days post and after 3 months of rest	Significant findings	Serious	C (1)
Soriano et al. [52]	Semi-acute	Cohort study	Field	Am. football	33 collegiate athletes, aged 19.3 ± 1.4	N/A	Am. football season (games and training)	Serum	Mid-season, post-season and off-season (after a rest period)	No significant findings	Serious	B (4)
Symons et al. [53]	Chronic	Cross-sectional study	Other	Au. football	95 (69 M, aged 23.3 ± 0.4; 26 F, aged 23.2 ± 0.9) amateur players	49 (28 M, aged 22.5 ± 0.4; 21 F, aged 23.1 ± 0.8) amateur basketball, tennis, cricket, track and field athletes	Au. football participation	Serum	N/A (pre-season)	No significant findings	Moderate	B (4)
Wallace et al. [90]	Acute	Prospective controlled cohort study	Laboratory	Soccer	11 male collegiate players, aged 23.7 ± 3.9	N/A	40 headers; sham condition: contact with ball using hands, chest or thigh	Serum	Immediately before and 1 h and 3 weeks post	Mixed	Critical	C (2)
Wirsching et al. [96]	Acute	Randomized controlled trial	Laboratory	Soccer	18 (7 M, 11F), aged 20.3 ± 1.5	16 (6 M, 10F), aged 21.2 ± 1.4	10 soccer headers; controls: 10 kicks	Plasma	Before and 0, 2 and 24 h post	Significant findings	Low	A (5)
Zetterberg et al. [47]	Acute and chronic	Longitudinal cohort study	Field	Boxing	14 (11 M, 3F) amateur boxers, aged 22 ± 3.8	10 male nonathletic controls, aged 30 ± 6.3	Boxing bout	CSF	7–10 days post and after 3 months of rest	Significant findings	Moderate	C (0)
Zetterberg et al. [48]	Acute	Non-randomized experimental study	Laboratory	Soccer	23 male amateur soccer players, median age (range): 10 headers *n *= 10, 26 (19–32); 20 headers *n *= 13, 23 (20–28)	9 male non athletes, median age (range) 24 (22–27)	10 or 20 standing headers from a corner kick (kicked from 30 m)	CSF	7–10 days post	Not detectable	Serious	B (3)
*GFAP*												
Asken et al. [55]	Chronic	Observational cohort study	Other	Soccer, diving, wrestling, ice hockey, Am. football	415 (256 M, 159 F) collegiate athletes, aged 19.0 ± 1.2	N/A	Cumulative exposure to collision sports in yr (and modified CHII)	Serum	Off-season	No significant findings	Moderate	B (4)
DiBattista et al. [57]	Chronic	Cross-sectional study	Other	Ice hockey, football, rugby, lacrosse	41 (39 M, 2 F) collision sport athletes; aged (including all participants): M (*n *= 60) 19.5 ± 2.0, F (*n *= 27) 19.5 ± 1.8	46 (21 M, 25 F) non collision sport athletes (inadvertent contact: soccer, basketball)	Collision sport participation	Plasma	Before the start of varsity season	No significant findings	Moderate	B (4)
Hoffman et al. [61]	Acute	Cohort study	Field	Am. football	15 Israel national football team players, aged 26.2 ± 5.3	N/A	Am. football match	Serum	1 week before, immediately (< 30 min) and 24 h post	No significant findings	Moderate	C (2)
Joseph et al. [42]	Acute and semi-acute	Prospective observational cohort study	Field	Am. football	16 male high-school varsity footballers, aged 16.9 ± 0.2 (pre- and post-season testing *n *= 12)	N/A	Am. football games, practices and season	Serum	Semi-acute: pre- and post-season; acute: 1–2 h post	No significant findings	Moderate	B (3)
Kawata et al. [82]	Semi-acute	Cohort study	Field	Ice hockey	8 male professional players (including 2 concussed athletes), aged 26.6 ± 1.6	N/A	Ice hockey season	Plasma	Pre- and post-season	No significant findings	Critical	C (1)
Major et al. [83]	Chronic	Cross-sectional study	Other	Au. football	81 (50 M, 31 F) amateur footballers (no-mTBI history *n *= 42; mTBI history *n *= 39), aged ~ 24	42 (23 M, 19 F) age-matched non-contact sport athletes	Au. football participation	Serum	Pre-season	No significant findings	Moderate	A (5)
Neselius et al. [46]	Acute and semi-acute	Prospective cohort study	Field	Boxing	30 (28 M, 2 F) Olympic boxers, mean age (range) 22 (17–34)	25 (20 M, 5 F) healthy controls, mean age (range) 22 (17–30)	Boxing bout	CSF	1–6 days post and after ≥ 14 days rest	Significant findings	Moderate	C (2)
Neselius et al. [66]	Acute and semi-acute	Prospective cohort study	Field	Boxing	30 (28 M, 2 F) amateur boxers (competing at elite level), mean age (range) 22 (17–34)	25 (20 M, 5 F) healthy controls, mean age (range) 22 (17–30)	Boxing bout	Serum	1–6 days post and after ≥ 14 days rest	GFAP not detectable	Serious	C (2)
Nowak et al. [85]	Acute	Case–control study	Laboratory	Soccer	17 (6 M, 11 F) soccer players with ADHD, aged 20.2 ± 0.2; 17 (10 M, 6 F) w/o ADHD, aged 21.1 ± 0.1	17 (7 M, 10 F) soccer players with ADHD, aged 20.5 ± 0.1	10 linear headers; controls: 10 kicks	Plasma	Baseline, 2 and 24 h post	Significant findings (ADHD cohort only)	Low	A (5)
O'Keeffe et al. [68]	Acute and semi-acute	Cohort study	Field	Rugby	8 rugby university team players, mean age (range) 22.1 (18–23); 11 male rugby school team players, mean age 17.4	27 non-contact sport athletes, median age (range) 28 (18–36); 26 healthy non-athlete controls, median age (range) 30 (18–40)	Rugby match (university team) and season (school and university team)	Plasma	University team: pre-season, ≤ 2 h post-match, 2 months post-season School team: pre- and post-season	GFAP not detectable	Moderate	B (3)
Soriano et al. [52]	Semi-acute	Cohort study	Field	Am. football	33 collegiate athletes, aged 19.3 ± 1.4	N/A	Am. football season (games and training)	Serum	Mid-season, post-season and off-season (after a rest period)	Significant findings	Serious	B (4)
Zetterberg et al. [47]	Acute and chronic	Longitudinal cohort study	Field	Boxing	14 (11 M, 3 F) amateur boxers, aged 22 ± 3.8	10 male nonathletic controls, aged 30 ± 6.3	Boxing bout	7–10 days post and after 3 months of rest	7–10 days post and after 3 months of rest	Acute: significant findings Chronic: no significant findings	Moderate	C (0)
Zetterberg et al. [48]	Acute	Non-randomized experimental study	Laboratory	Soccer	23 male amateur soccer players, median age (range): 10 headers *n *= 10, 26 (19–32); 20 headers *n *= 13, 23 (20–28)	9 male non athletes, median age (range) 24 (22–27)	10 or 20 standing headers from a corner kick (kicked from 30 m)	CSF	7–10 days post	No significant findings	Serious	B (3)
Zetterberg et al. [77]	Chronic	Observational case–control study	Other	Boxing	44 male amateur boxers, median age (range) 19 (17–28)	23 healthy males w/o contact sport history, median age (range) 28 (19–50)	Boxing participation (boxing debut, boxing duration in yr, number of bouts)	Serum	After a 2-month period of nonparticipation in boxing	GFAP not detectable	Moderate	C (1)
*NSE*												
DiBattista et al. [57]	Chronic	Cross-sectional study	Other	Ice hockey, football, rugby, lacrosse	41 (39 M, 2F) collision sport athletes; aged (including all participants): M (*n *= 60) aged 19.5 ± 2.0, F (*n *= 27) aged 19.5 ± 1.8	46 (21 M, 25F) non collision sport athletes (inadvertent contact: soccer, basketball)	Collision sport participation	Plasma	Before the start of varsity season	No significant findings	Moderate	B (4)
Graham et al. [59]	Acute	Retrospective cohort study	Field	Boxing	8 male amateur boxers, aged 17.6 ± 5.3 (PTH – punches to the head and body)	8 male amateur boxers, aged 19.1 ± 3.2 (PTB – punches to the body)	5 × 2-min boxing rounds	Serum	1 h before and after 5 min of cessation	Significant findings	Moderate	C (2)
Graham et al. [60]	Acute	Cohort study	Field	Karate	12 males, aged 30.4 ± 6.7 (KTH – kicks to the head and body)	12 males, aged 28.2 ± 6.5 (KTB – kicks to the body)	4 × 3-min karate round	Serum	Before and immediately after	Significant findings	Moderate	C (1)
Horner et al. [97]	Acute	Cohort study	Field	Boxing	8 male Olympic boxers, age range 18–28	17 male amateur oarsmen, age range 18–23	3 × 3-min boxing rounds; controls: 6-min ergometer test	Serum	Before and after	Significant findings	Moderate	C (1)
Rogatzki et al. [71]	Acute	Cohort study	Field	Am. football	17 male Division III collegiate footballers, aged 19.5 ± 0.9	N/A	Am. football game	Serum	2 days before and 1 h post	Significant findings	Moderate	C (2)
Stålnacke et al. [74]	Acute	Cohort study	Field	Ice hockey	26 male elite ice hockey players, aged 28 ± 4	18 elite basketball players, aged 25 ± 4	Ice hockey game (body checkings, falls, collisions, boardings); basketball game (jumps, collisions, falls)	Serum	1–2 h before and within 1 h post	No significant findings	Serious	C (2)
Stålnacke et al. [75]	Acute	Cohort study	Field	Soccer	28 male elite players, aged 26 ± 5	N/A	Headers, jumps, falls and collision during a competitive soccer game	Serum	1–5 h before and immediately post	Significant findings	Moderate	B (3)
Stålnacke et al. [38]	Acute	Cohort study	Field	Soccer	44 female elite players, aged 23 ± 3	N/A	Headers, jumps, falls and collisions during a competitive soccer game	Serum	Before and immediately post	Significant findings	Moderate	B (3)
Zetterberg et al. [77]	Chronic	Observational case–control study	Other	Boxing	44 male amateur boxers, median age (range) 19 (17–28)	23 healthy males w/o contact sport history, median age (range) 28 (19–50)	Boxing participation (boxing debut, boxing duration (yr), number of bouts)	Serum	After a 2-month period of nonparticipation in boxing	Significant findings	Moderate	C (1)
*BDNF*												
Bamaç et al. [98]	Acute	Non-randomized experimental study	Laboratory	Soccer	17 male professional soccer players, aged 24.6 ± 4.4	N/A	15 jumping soccer headers; headed from a corner kick	Serum	Before and after	Significant findings	Serious	B (4)
DiBattista et al. [57]	Chronic	Cross-sectional study	Other	Ice hockey, football, rugby, lacrosse	41 (39 M, 2 F); age for all sample: M (n = 60) 19.5 ± 2.0, F (*n *= 27) 19.5 ± 1.8	46 (21 M, 25 F) non collision sport athletes (inadvertent contact: soccer, basketball)	Collision sport participation	Plasma	Before the start of varsity season	No significant findings	Moderate	B (4)
Hoffman et al. [61]	Acute	Cohort study	Field	Am. football	15 Israel national football team players, aged 26.2 ± 5.3	N/A	Am. football match	Serum	1 week before, immediately (< 30 min) and 24 h post	Significant findings	Moderate	C (2)
Neselius et al. [66]	Acute and semi-acute	Prospective cohort study	Field	Boxing	30 (28 M, 2 F) amateur boxers (competing at elite level), mean age (range) 22 (17–34)	25 (20 M, 5 F) healthy controls, mean age (range) 22 (17–30)	Boxing bout	Serum	1–6 days post and after ≥ 14 days rest	No significant findings	Serious	C (2)
O'Keeffe et al. [68]	Acute and semi-acute	Cohort study	Field	Rugby	8 rugby university team players, mean age (range) 22.1 (18–23); 11 male rugby school team players, mean age 17.4	27 non-contact sport athletes, median age (range) 28 (18–36); 26 healthy non-athlete controls, median age (range) 30 (18–40)	Rugby match (university team) and season (school and university team)	Plasma	University team: pre-season, ≤ 2 h post-match, 2 months post-season School team: pre- and post-season	Acute: no effects Semi-acute: significant findings	Moderate	B (3)
Oztasyonar [99]	Acute	Cohort study	Field	Boxing, Tae Kwon Do	20 male boxers, aged 20.15 ± 1.52; 20 male Tae Kwon Do fighters, aged 20.60 ± 1.65	20 male runners, aged 19.87 ± 1.60; 20 sedentary participants, aged 20.40 ± 1.85	Boxing: 3 × 3-min rounds; Tae Kwon Do: 2 × 3-min rounds; controls: running	Serum	Immediately before and after	Significant findings	Moderate	C (1)
Zetterberg et al. [77]	Chronic	Observational case–control study	Other	Boxing	44 male amateur boxers, median age (range) 19 (17–28)	23 healthy males w/o contact sport history, median age (range) 28 (19–50)	Boxing participation (boxing debut, boxing duration in yr, number of bouts)	Serum	After a 2-month period of nonparticipation in boxing	No significant findings	Moderate	C (1)
*UCH-L1*												
Asken et al. [55]	Chronic	Observational cohort study	Other	Soccer, diving, wrestling, ice hockey, Am. football	415 (256 M, 159 F) collegiate athletes, aged 19.0 ± 1.2	N/A	Cumulative exposure to collision sports in yr (and modified CHII)	Serum	Off-season	No significant findings	Moderate	B (4)
Joseph et al. [42]	Acute and semi-acute	Prospective observational cohort study	Field	Am. football	16 male high-school varsity footballers, aged 16.9 ± 0.2 (pre- and post-season testing *n *= 12)	N/A	Am. football games, practices and season	Serum	Semi-acute: pre- and post-season; acute: 1–2 h post	Significant findings	Moderate	B (3)
Major et al. [83]	Chronic	Cross-sectional study	Other	Au. football	81 (50 M, 31 F) amateur footballers (no-mTBI history *n *= 42; mTBI history *n *= 39), aged ~ 24	42 (23 M, 19 F) age-matched non-contact sport athletes	Au. rules football participation	Serum	N/A	No significant findings	Moderate	A (5)
Nowak et al. [85]	Acute	Case–control study	Laboratory	Soccer	17 (6 M, 11 F) soccer players with ADHD, aged 20.2 ± 0.2; 17 (10 M, 6 F) w/o ADHD, aged 21.1 ± 0.1	17 (7 M, 10 F) soccer players with ADHD, aged 20.5 ± 0.1	10 linear headers; control: 10 kicks	Plasma	Baseline, 2 and 24 h post	Significant findings (for ADHD cohort only at 24 h post)	Low	A (5)
Puvenna et al. [70]	Acute	Cohort study	Field	Am. football	15 athletes	406 positive controls with mTBI and 465 negative controls	2 Am. football games	Serum	Baseline (day before) and post (< 1 h) (positive controls: < 6 h of injury)	Significant findings. No correlation with head hits	Serious	B (3)
Soriano et al. [52]	Semi-acute	Cohort study	Field	Am. football	33 male collegiate players, aged 19.3 ± 1.4	N/A	Am. football season (games and training)	Serum	Mid-season, post-season and off-season (after a rest period)	UCH-L1 levels not detectable (for majority of samples)	Serious	B (4)

*Am. football* American football, *Au football* Australian Rules football, *CHII* Cumulative head impact index, *MMA* Mixed martial arts, *NCAA* National Collegiate Athletic Association, *NFL* National Football League

**Fig. 3:**
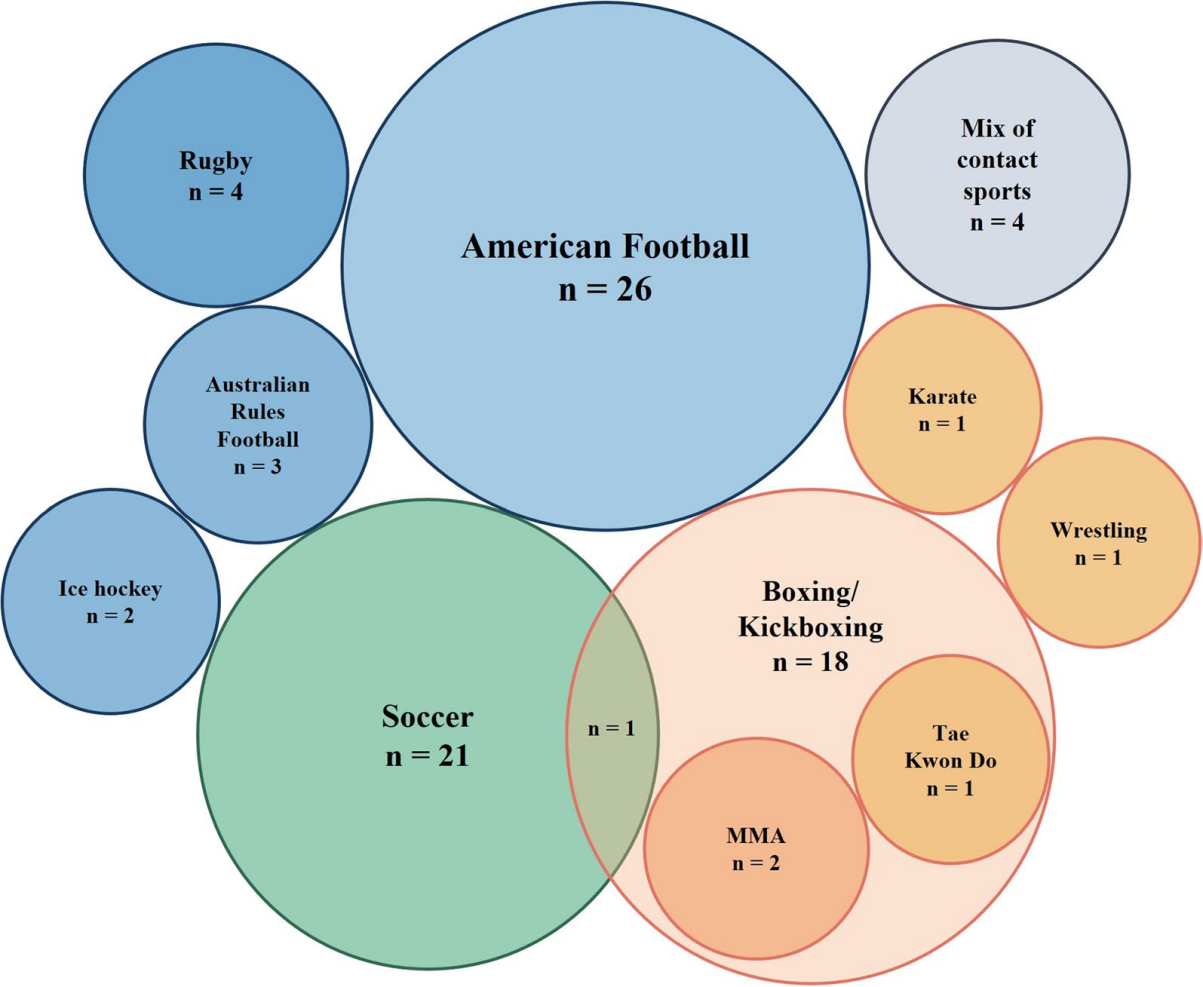
Number of articles per sport (MMA—mixed martial arts)

American football was the most studied sport with 26 studies, followed by soccer with 21, and boxing with 18 (including 2 kickboxing) studies (Fig. 3).

Fifteen research reports (~ 19%) provided a definition for subconcussive head impacts (definitions provided in Additional file 1: Table S5). Forty-seven studies (~ 60%) quantified or estimated RSHI exposure. Of the acute and semi-acute studies, 12 employed the use of accelerometers to document impact (see Additional file 1: Table S6 for impact information). Five of the 12 studies assessed impact data from soccer heading and six studies assessed RSHI metrics in American football. Where reported, peak average (or median) linear acceleration per impact ranged from 13.3 to 114.7 g.

Thirty (~ 38%) of the studies included an additional outcome measure other than biofluid marker(s) to assess the effects of RSHI. Commonly used measures included brain imaging (in 9 studies) and neurocognitive tests, motor control, and/or concussion symptom assessment (in 26 studies); five studies had a multimodal approach integrating brain imaging and neurocognitive tests, motor control, and/or concussion symptom assessment.

### Methodological Quality of Evidence

**Fig. 4:**
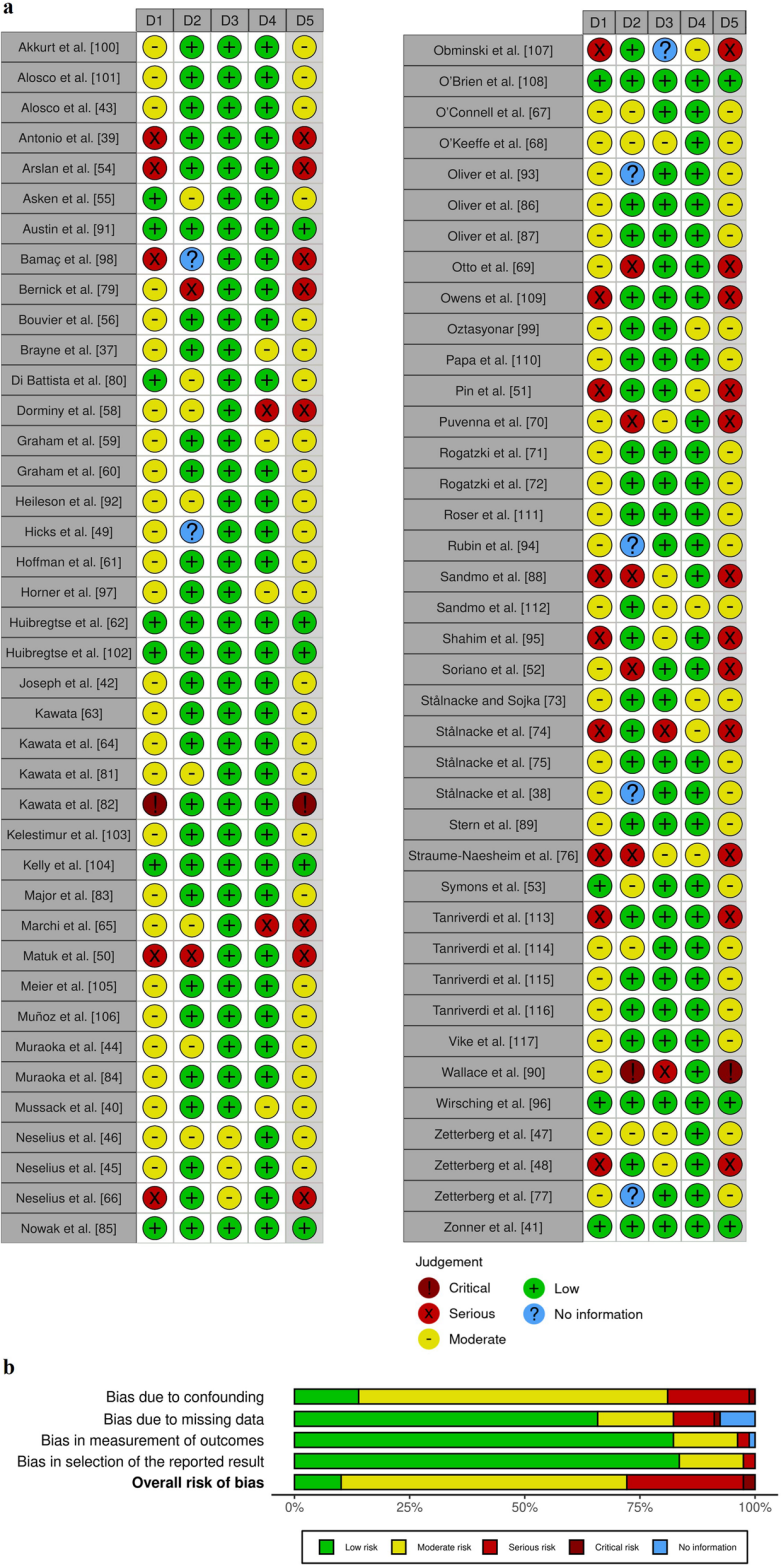
**a** Review authors’ rating for individual risk of bias domains and the overall score for each study; **b** An applicability concerns graph summarizing the pooled risk of bias score for each domain as a percentage. D1: Bias due to confounding; D2: Bias due to missing data; D3: Bias in measurement of outcomes; D4: Bias in selection of the reported results; D5: Overall bias result

Based on our analysis of the risk of bias, two studies (~ 2.5%) were scored as critical, 20 (~ 25%) as serious, 49 (~ 62%) as moderate, and only 8 (~ 10%) of the studies received a low risk of bias rating. Most studies that received moderate or higher risk of bias did so due to failing to control for confounding variables (Fig. 4). The findings of all identified studies are considered in this review to fully scope the body of evidence.

Specific subconcussion methodological quality assessment results are displayed in Table 2; ~ 46% of studies received a category C (*n *= 36), ~ 40.5% category B (*n *= 32), and ~ 14% category A (*n *= 11) rating meeting almost all of the criteria with regard to subconcussion methodological quality. The most common unmet criteria were a failure to provide a definition for RSHI or account for sex differences.

**Table 2:** Quality assessment outcomes for individual studies using a modified version of the Subconcussion-Specific Tool

Reference	1	2	3	4	5	6	Category	Score
Akkurt et al. [100]	No	Yes	Yes	Yes	Yes	No	B	4
Alosco et al. [101]	No	Yes	Yes	No	No	No	C	2
Alosco et al. [43]	No	Yes	Yes	No	No	No	C	2
Antonio et al. [39]	No	No	Yes	No	Yes	No	C	2
Arslan et al. [54]	No	No	No	No	Yes	No	C	1
Asken et al. [55]	No	No	Yes	Yes	Yes	Yes	B	4
Austin et al. [91]	No	Yes	Yes	Yes	Yes	Yes	A	5
Bamaç et al. [98]	No	Yes	Yes	Yes	Yes	No	B	4
Bernick et al. [79]	No	No	No	No	Yes	No	C	1
Bouvier et al. [56]	No	Yes	Yes	No	Yes	Yes	B	4
Brayne et al. [37]	No	Yes	No	No	Yes	No	C	2
Di Battista et al. [80]	No	No	Yes	Yes	Yes	Yes	B	4
Dorminy et al. [58]	No	Yes	Yes	Yes	Yes	No	B	4
Graham et al. [59]	No	Yes	No	No	Yes	No	C	2
Graham et al. [60]	No	No	No	No	Yes	No	C	1
Heileson et al. [92]	No	No	Yes	No	Yes	No	C	2
Hicks et al. [49]	No	Yes	Yes	No	Yes	No	B	3
Hoffman et al. [61]	No	No	Yes	No	Yes	No	C	2
Horner et al. [97]	No	No	No	No	Yes	No	C	1
Huibregtse et al. [62]	Yes	Yes	Yes	Yes	Yes	No	A	5
Huibregtse et al. [102]	No	Yes	Yes	Yes	Yes	No	B	4
Joseph et al. [42]	No	Yes	Yes	No	Yes	No	B	3
Kawata [63]	Yes	Yes	Yes	Yes	Yes	Yes	A	6
Kawata et al. [64]	Yes	Yes	Yes	Yes	Yes	No	A	5
Kawata et al. [81]	Yes	Yes	Yes	Yes	Yes	No	A	5
Kawata et al. [82]	No	No	No	No	Yes	No	C	1
Kelestimur et al. [103]	No	No	Yes	No	Yes	No	C	2
Kelly et al. [104]	No	No	Yes	Yes	Yes	No	B	3
Major et al. [83]	Yes	No	Yes	Yes	Yes	Yes	A	5
Marchi et al. [65]	No	Yes	Yes	Yes	Yes	No	B	4
Matuk et al. [50]	No	No	No	No	No	Yes	C	1
Meier et al. [105]	No	No	Yes	Yes	Yes	No	B	3
Muñoz et al. [106]	No	Yes	Yes	Yes	No	No	B	3
Muraoka et al. [44]	No	Yes	Yes	No	Yes	No	B	3
Muraoka et al. [84]	No	Yes	Yes	No	Yes	No	B	3
Mussack et al. [40]	No	No	Yes	Yes	Yes	No	B	3
Neselius et al. [46]	No	No	No	Yes	Yes	No	C	2
Neselius et al. [45]	No	No	No	Yes	Yes	No	C	2
Neselius et al. [66]	No	No	No	Yes	Yes	No	C	2
Nowak et al. [85]	Yes	Yes	Yes	Yes	Yes	Yes	A	5
Obminski et al. [107]	No	No	No	No	No	No	C	0
O’Brien et al. [108]	No	No	Yes	Yes	Yes	Yes	B	4
O'Connell et al. [67]	No	No	Yes	No	No	No	C	1
O'Keeffe et al. [68]	Yes	No	No	Yes	Yes	No	B	3
Oliver et al. [93]	No	No	Yes	No	Yes	No	C	2
Oliver et al. [86]	Yes	No	Yes	No	Yes	No	B	3
Oliver et al. [87]	Yes	No	Yes	No	Yes	No	B	3
Otto et al. [69]	No	Yes	No	No	No	No	C	1
Owens et al. [109]	No	Yes	No	No	Yes	No	C	2
Oztasyonar [99]	No	No	No	No	Yes	No	C	1
Papa et al. [110]	No	No	No	Yes	No	Yes	C	2
Pin et al. [51]	Yes	Yes	Yes	Yes	Yes	Yes	A	6
Puvenna et al. [70]	Yes	Yes	Yes	No	No	No	B	3
Rogatzki et al. [71]	No	No	Yes	No	Yes	No	C	2
Rogatzki et al. [72]	No	Yes	Yes	No	Yes	No	B	3
Roser et al. [111]	No	No	Yes	No	Yes	No	C	2
Rubin et al. [94]	Yes	Yes	Yes	Yes	Yes	Yes	A	6
Sandmo et al. [88]	No	Yes	Yes	No	Yes	No	B	3
Sandmo et al. [112]	No	Yes	Yes	No	Yes	No	B	3
Shahim et al. [95]	No	No	No	No	Yes	No	C	1
Soriano et al. [52]	Yes	No	Yes	Yes	Yes	No	B	4
Stålnacke and Sojka [73]	No	Yes	Yes	No	Yes	No	B	3
Stålnacke et al. [74]	No	Yes	No	No	Yes	No	C	2
Stålnacke et al. [75]	No	Yes	Yes	No	Yes	No	B	3
Stålnacke et al. [38]	No	Yes	No	No	Yes	Yes	B	3
Stern et al. [89]	No	No	Yes	No	Yes	No	C	2
Straume-Naesheim et al. [76]	No	Yes	Yes	No	Yes	No	B	3
Symons et al. [53]	No	No	Yes	Yes	Yes	Yes	B	4
Tanriverdi et al. [113]	No	No	No	No	Yes	No	C	1
Tanriverdi et al. [114]	No	No	Yes	No	Yes	No	C	2
Tanriverdi et al. [115]	No	No	Yes	No	Yes	No	C	2
Tanriverdi et al. [116]	No	No	Yes	No	Yes	No	C	2
Vike et al. [117]	No	Yes	No	Yes	Yes	No	B	3
Wallace et al. [90]	No	Yes	No	No	Yes	No	C	2
Wirsching et al. [96]	Yes	Yes	Yes	Yes	Yes	No	A	5
Zetterberg et al. [47]	No	No	No	No	No	No	C	0
Zetterberg et al. [48]	No	Yes	Yes	No	Yes	No	B	3
Zetterberg et al. [77]	No	No	Yes	No	No	No	C	1
Zonner et al. [41]	Yes	Yes	Yes	Yes	Yes	No	A	5

Category A: met five or more criteria, Category B: met three or four criteria and Category C: met two or less criteria. Domains assessed: (1) Was there an attempt to define the term ‘subconcussion’? (2) Was the number or magnitude of impacts reported? (note that if impacts were recorded however not reported but impact data were included in the analysis, then the criterion was considered met) (3) Were subjects who sustained a concussion during the study controlled for or excluded from analyses? (4) Were subjects with a history of concussion controlled for or excluded from the analyses? (5) Was the control group matched on two or more variables (e.g., history of concussion, history of contact sport participation, age etc.)? (6) Did the study analyze sex differences, or acknowledge limitations associated with sampling only males or females?

### Summaries for the Most Studied Biofluid Markers

#### S100 Calcium-Binding Protein beta (S100B)

Glial injury marker S100B was the most examined protein with 30 studies (Table 1), with a median study sample size of 29 (range 8 to 415) contact sport athletes. Twenty-four studies assessed the acute effects of RSHI on S100B concentrations in blood [38, 40, 41, 48, 54, 56, 58–62, 64–76], of which 17 studies found a significant increase in S100B within two hours of RSHI exposure [38, 41, 54, 56, 59, 60, 64, 65, 67–72, 74–76] (range 1.3–5.3-fold, 26%-431% increase). All 17 studies were field-based, where the effect of physical activity could not be eliminated. Eight of the 17 studies employed a control group or condition investigating the effect of exercise and/or peripheral injuries on S100B levels [59, 60, 64, 67, 69, 72, 74, 76]. Critically, in six of the eight studies, a significant increase in S100B was observed also in the control group or control condition [64, 67, 69, 72, 74, 76]. Overall, S100B increased 1.3–1.8-fold (26–78% increase) following exercise alone [67, 72, 76]. Laboratory-based studies investigating the effects of soccer heading, where physical activity was controlled, reported no effect of RSHI on S100B [40, 48, 58, 69, 73, 118].

Increases in S100B were found to be significantly correlated with impact metrics, with studies reporting correlation coefficients ranging from 0.43 to 0.66. However, one study reported a correlation between increases in S100B and the number of jumps in a basketball game (*r *= 0.71) [74].

Two studies measured S100B in CSF following exposure to RSHI. One study reported significantly (∼1.2-fold) higher S100B concentrations in CSF (but not in serum) 1–6 days after a boxing bout compared to the controls [46, 66]. In the other study, S100B levels in CSF and serum were not significantly higher compared to the control group 7–10 days after controlled soccer heading [48]. Overall, S100B appears to increase following RSHI only if accompanied by physical exertion and the marker is sensitive to the effect of exercise regardless of head impacts.

None of the nine studies that assessed the semi-acute effects of RSHI found a significant increase in S100B [41, 46, 52, 63, 65–68, 72]. Also, no relationship between prior contact sport exposure and S100B was found in the three studies that investigated the chronic effects of RSHI in active contact sport athletes following a period of rest (∼2–6 months) from contact sport participation [55, 77, 80]. Overall, semi-acute and chronic RSHI exposure does not appear to cause elevations in S100B levels.

#### Tau

Twenty-four studies examining the effects of RSHI on tau were identified (all 24 studies assessed total tau levels), with a median study sample size of 32 (range 8 to 415) contact sport athletes. Information about study type and design, type of exposure, and participant characteristics can be found in Table 1. Of these, 11 studies examined the acute effects of RSHI on tau concentrations; six studies had a moderate or low risk of bias, four of which reported significant tau increases after impacts incurred in boxing [46, 47] and American football [42, 81], the other two did not report significant findings after soccer heading [85] and an American football match [61]. Eight studies investigated the semi-acute effects of RSHI yielding mixed findings. Four of the studies found no significant differences in tau concentrations [46, 66, 86, 119], while two found significant increases [42, 79] (albeit one of these studies found yearly increases only in active mixed martial arts fighters but not in boxers [79]) and another a significant decrease [87] (one study failed to detect tau in serum [52]). Twelve of the 24 studies examined if RSHI causes chronic tau increases, of which six did not find significant differences [44, 47, 55, 79, 83, 88], while four studies found increased tau levels [53, 80, 84, 89]. Furthermore, two studies found a correlation between RSHI career exposure and tau concentrations, although the concentrations were not significantly different to those of controls [43, 78]. Therefore, although tau is one of the markers currently receiving the most attention (see Fig. 2), its utility in evidencing the effects of RSHI in contact sport is uncertain.

Seven of the aforementioned 24 studies also examined phosphorylated tau (p-tau). Two investigated the acute effects of RSHI on p-tau, finding no significant differences [46, 47]. One study [46] also investigated the semi-acute effects, again reporting no significant results. The chronic sequelae of RSHI on p-tau concentrations in active and former athletes were investigated in six studies, with four of them reporting no significant effects in American [43, 44] and Australian Rules football players [83], or boxers [47], while two studies reported significant 1.2–1.8-fold increases in former and active American football players [53, 84]. All seven studies had a moderate risk of bias. Overall, the utility of p-tau in evidencing the effects of RSHI in contact sports is uncertain.

#### Neurofilament Light (NfL)

Twenty studies examining the effects of RSHI on NfL concentrations were identified, with a median study sample size of 32 (range 8 to 338) contact sport athletes. Information about study type and design, type of exposure, and participant characteristics can be found in Table 1. Twelve studies investigated the acute effects of RSHI on NfL concentration, with eight of them reporting a significant increase (boxing *n *= 4, soccer heading *n *= 3, American football *n *= 1) [46, 47, 79, 85, 90, 94–96]. NfL levels increased ~ 1.2–1.9-fold when sampled from serum compared to baseline levels or controls [79, 85, 90, 95, 96] and 4.1-fold in CSF [47]. The earliest increase was observed ~ 1 h post-RSHI exposure [90, 94], with the majority of the studies finding a significant increase at ≥ 24 h [46, 47, 79, 85, 95, 96]. Two of the eight studies sampled NfL from CSF [46, 47], with one study demonstrating that increases in CSF NfL concentration were positively correlated with serum NfL levels [47, 95]. Five of the acute studies also suggested a dose–response relationship between impact exposure (severity and/or quantity) and NfL levels [46, 47, 79, 94, 95]. Five of the seven studies identified as having a low or moderate risk of bias reported significant effects. NfL levels were not detectable in one of the 12 studies [48].

Significant increases were also reported in five of the eight studies that examined the semi-acute effects of RSHI in American football [87, 92, 93], boxing [46], and ice hockey [82]. Seven studies investigated whether chronic RSHI exposure results in elevated NfL levels, with three of them reporting significantly higher NfL levels than in controls (~ 2 times higher) [39, 47, 95]. However, none of the three studies investigated the relationship between lifetime exposure to RSHI and NfL levels, and all three studies were conducted in active contact sport athletes. Therefore, of the ‘up-and-coming’ biomarkers (see Fig. 2), NfL appears as one of the most promising in demonstrating the effects of RSHI on the brain, irrespective of the sport.

#### Glial Fibrillary Acidic Protein (GFAP)

Fourteen studies assessing the effects of RSHI on GFAP were identified with a median study sample size of 30 (range 8 to 415) contact sport athletes (see Table 1 for details on study type and design, type of exposure, and participant characteristics). Eight studies investigated the acute effects [42, 46–48, 61, 66, 68, 85] (three in CSF [46–48]), of which three studies found significant increases (1.3-2-fold) in GFAP levels following a boxing bout (in CSF) [46, 47] and soccer heading (in plasma) [85]. GFAP was not detectable (in serum/plasma) in two of the studies [66, 68].

Six studies assessed the semi-acute effects of RSHI by measuring GFAP concentrations [42, 46, 52, 66, 68, 82] (one in CSF [46]), with only one study reporting a significant increase [52]. All five chronic studies (carried out in active athletes) found no effect of RSHI on GFAP levels [47, 55, 57, 77, 83]. GFAP was not detectable (in serum/plasma) in three studies: two assessing semi-acute [66, 68] and one assessing chronic effects [77].

With regard to methodological constraints, the limits of detection for the assays failing to detect GFAP levels were 150 and 780 ng/L. Overall, GFAP appears not to be affected by RSHI; however, this conclusion is subject to limited evidence.

#### Neuron-Specific Enolase (NSE)

Nine studies investigating the effects of RSHI on NSE concentrations were identified, with a median study sample size of 26 (range 8–44) contact sport athletes (Table 1). Seven studies assessed the acute effects, of which all studies with moderate risk of bias (*n *= 6) reported significant findings, with soccer games causing a 1.1-2-fold increase in NSE [38, 75], American football a 1.9-fold increase [71], and boxing a 1.6–2.5-fold increase [59, 97]. The two studies examining the chronic effects yielded mixed results [77, 80]. Therefore, higher quality studies including NSE to examine the effects of RSHI showed promise, demonstrating the acute effects of head impact in sport.

#### Brain-Derived Neurotrophic Factor (BDNF)

Seven studies assessing the effects of RSHI on BDNF were found, with a median study sample size of 30 (range 15 to 44) contact sport athletes (Table 1). The acute effects were assessed in five studies, yielding mixed results [61, 66, 68, 98, 99]. BDNF was found to increase after boxing and taekwondo training [99] and an American football game [61], but not after a rugby match [68], in studies with a moderate risk of bias. The two studies that were identified as having a serious risk of bias showed increased BDNF after soccer heading [98] but no effects after a boxing bout [66]. Two studies investigated the semi-acute effects [66, 68], with one revealing increased BDNF concentrations after a rugby season [68], and two studies investigated the chronic effects without finding evidence of BDNF alterations [77, 80]. Therefore, BDNF as a measure appears to reveal little about the effect of RSHI in sport.

#### Ubiquitin C-Terminal Hydrolase L1 (UCH-L1)

Six studies used UCH-L1 [42, 52, 55, 70, 83, 85] to investigate the effects of RSHI on athletes’ brain health (see Table 1 for details), with the median sample size of 34 (range 15 to 415) contact sport athletes. Three studies reported a significant increase in UCH-L1 levels acutely following RSHI exposure [42, 70, 85]. Two studies also assessed UCH-L1 concentrations in semi-acute and two in chronic settings. One of the semi-acute studies found a significant increase in UCH-L1 concentrations following a season of American football [42], whereas the majority of the samples were not quantifiable in the other study [52]. Neither of the studies assessing the chronic effects of RSHI found increased UCH-L1 levels [55, 83]. Therefore, UCH-L1 appears to be increased acutely but not chronically following RSHI exposure; however, the evidence thus far is limited.

#### Hormonal Studies

Nine studies investigated the effects of RSHI on the hormonal response (see Additional file 1: Table S7), with the median sample size of 22 (range 11 to 68) contact sport athletes. One case study reported the acute and semi-acute effects of RSHI on hormone levels in a kickboxer [113], and eight studies reported the chronic effects [100, 103, 104, 107, 111, 114–116]. Five studies that examined the chronic effects of RSHI in boxing and American football revealed growth hormone secretory deficiencies [103, 104, 114, 115], anti-hypothalamus and anti-pituitary antibodies presence [116], insulin-like growth factor 1 [103, 114] and adrenocorticotropic hormone [114, 115] deficiency, and hypogonadism [104]. RSHI exposure in soccer players revealed no long-term effects on hormonal responses [100, 111]. Overall, sustained exposure to RSHI appears to increase the risk of pituitary dysfunction in contact sport athletes.

## Discussion

This scoping review provides a broad overview of the currently available evidence on the effects of RSHI on biofluid marker levels. We identified 79 studies, with research in this field demonstrating exponential growth (Fig. 2). This review sheds light on a significant body of evidence not previously identified, i.e., two previous systematic reviews on the same topic identified just five relevant papers each [21, 22]. The discrepancy in the number of relevant articles identified in the current and prior reviews is perhaps caused by the latter either not focusing solely on biofluid markers, thus including fewer biomarker-specific keywords in their search strategy [21, 22], or focusing on specific study designs [21].

The findings of our review demonstrate that acute effects of RSHI have been studied most (*n *= 49), while the number of studies assessing biofluid marker levels following semi-acute (*n *= 23) and chronic (*n *= 26) RSHI exposure are similar. Our inclusion criteria allowed us to identify a large panel of biofluid markers linked to traumatic brain injury, such as axonal damage, compromised blood–brain-barrier integrity and neurodegeneration. Although there were several interesting candidate biomarkers with fewer than five studies available, making marker-specific conclusions was not feasible due to methodological differences such as sampling times. Therefore, this review focused on detecting patterns in the most studied biofluid markers.

S100B, an astrocyte-enriched Ca2 + -binding protein that helps regulate intracellular calcium concentrations [120], was the most extensively studied biomarker. However, its utility for the purpose of examining RSHI effects in contact sports is questionable due to its extracerebral presence (S100B is also present in other tissues such as chondrocytes, adipocytes, and bone marrow cells) [120]. Indeed, we noted significant increases in S100B in the control group or control condition of several studies included in this review where exercise was involved [64, 67, 69, 72, 74, 76]. This is unsurprising considering that previous evidence has demonstrated S100B increases in athletes participating in noncontact sports without RSHI [121]. Consequently, although S100B demonstrated a dose–response relationship with impact metrics, this marker may not be suited for assessing the effects of RSHI in a sporting setting due to also being affected by exercise alone. Based on our findings, S100B shows very limited, if any, utility in detecting RSHI-induced changes in semi-acute and chronic settings.

GFAP, a cytoskeletal protein almost exclusively present in astrocytes [122], demonstrated no effects in the majority (~ 67%) of the studies investigating the acute effects of RSHI. Previously, GFAP has been shown to differentiate mild brain injury (Glasgow Coma Scale score 13–15 with clear MRI scans) from healthy control data [24]. Semi-acute and chronic levels of GFAP did not appear to be affected by RSHI exposure.

The axonal injury marker NfL [30] is, perhaps, the most promising of all the studied markers in demonstrating elevated levels acutely following RSHI exposure. Importantly, its levels appear to increase in a dose–response manner [46, 47, 79, 94]. NfL also demonstrated some promise in evidencing the semi-acute effects of RSHI. In contrast, tau, the second most studied marker in RSHI research (and abundant in thin unmyelinated cortical interneurons [30]) yielded mixed findings across acute, semi-acute, and chronic settings. Biofluid marker concentrations are known to scale to the severity of brain injury [123], and as such, it is possible that some studies did not find significant effects because the quantity and severity of the RSHI did not result in injury, whereas some studies may simply have failed to detect changes in marker concentrations due to methodologies surrounding assays and sampling times.

The most frequently studied neuronal injury markers, NSE and UCH-L1, had limited numbers of studies of RSHI available, and no conclusions could be drawn.

### Sampling Source and Time

The majority of the studies sampled biofluid markers from venous blood (*n *= 72), with only six studies assessing the concentrations in CSF and four in saliva. More studies sampling biomarkers from both blood and CSF are necessary to ensure that the changes in blood reflect changes in the central nervous system (CNS). This is particularly important for markers that are not specific to the CNS. Although the current research is not at a stage where blood or saliva samples can be reliably associated with brain alterations caused by RSHI exposure, the end goal in this field of research should be the identification of biofluid markers that can be sampled efficiently and non-invasively for the routine monitoring of athletes’ brain health.

In this review, we were unable to identify the most appropriate marker-specific sampling times following RSHI exposure. This was due to the mixed findings reported, the heterogeneity of the included studies, and the fact that most studies provided little, if any, justification surrounding sampling time choices. The time course of RSHI effects and how it relates to the changes in the levels of different biofluid markers is currently unclear. Critically, more research is needed, as discussed below.

### Quality and Limitations of the Identified Studies

A further aim of this scoping review was to assess the quality of the available evidence and identify research gaps in order to guide future research. We identified limitations in the following three main categories: (1) lack of appropriate control of confounding variables, (2) lack of impact monitoring, and (3) representativeness of the sampled populations. Similar concerns have been highlighted before in the field of RSHI in general [21, 22, 124].

We found that only ~ 10% of the studies could be considered to have a low risk of bias and that the primary domain increasing the bias was controlling for confounding variables. Common confounding variables that were not controlled include prior concussions, concussions occurring during the study, and the effect of exercise. Furthermore, 27 studies (34%) did not employ a control condition or a control group. It is imperative that future research utilizes control groups, or conditions to control for the effect of exercise, to ensure that changes in biomarker concentrations are not driven by confounding variables.

Another important limitation of the current evidence was the lack of monitoring and quantification of RSHI exposure. Strikingly, around 40% of the studies did not quantify or estimate RSHI exposure. Moreover, only 12 studies employed accelerometers to document impact. The sports where accelerometers were utilized most in the context of RSHI were American football (*n *= 6) and soccer (*n *= 5). There were no studies measuring impact magnitude in boxing, despite it being the third most studied sport. The number of studies assuming, rather than measuring, the occurrence of RSHI is concerning, especially since without data on impact metrics it is not possible to examine the dose–response relationship between impact exposure and brain changes. Furthermore, only 15 studies (~ 19%) provided a definition for RSHI. Not characterizing RSHI is an issue, especially if investigators do not separate RHSI from concussive impacts in research.

The studied samples were not fully representative of the population of interest, especially in respect to sex and age, hampering the generalizability of the results. There was limited evidence documenting the effects of RSHI exposure in females using biofluid markers. The majority of the studies (*n *= 42) were carried out in a male-only cohort, whereas there were only two female-only studies. While there were 20 studies that used a mixed-sex approach, only 14 compared or acknowledged sex differences. Concerningly, sex was not specified in 15 studies. We also identified only three studies done in a juvenile-only cohort (~ 13–17-year-olds), with retired contact sport athletes also being understudied, as the majority of the studies assessing chronic effects of RSHI were conducted in active contact sport athletes.

### Strengths and Limitations

The strengths of the current review are the adherence to an a priori-developed and published review protocol, following the PRISMA-ScR guidelines, and most importantly, the comprehensive search strategy used. The latter has reduced the risk of overlooking relevant research conducted in the field of RSHI and biofluid markers, and has enabled us to provide a full overview of the research done in this field from its inception until this review—an overview that was not available until now.

We acknowledge that this review has limitations. The generalizability of our findings is limited to sport-specific effects and may not be true for RSHI occurring in other settings (e.g., military, domestic abuse, etc.). Furthermore, concussion studies that employed a control group of contact sport athletes (where it was not clear whether RSHI had occurred) were not included in this review. As such, potentially relevant research may have been excluded from the current review; however, we believe that any such studies would have added little value for the purpose of this review due to the ambiguity surrounding the occurrence of RSHI.

### Future Research

Our analysis showed that many of the studies included in this review are highly variable and present issues in the study design, quality, and analysis, resulting in biased reporting. This review demonstrated that most of the current research does not define RSHI or quantify impact exposure (Table 2); this prevents studies from drawing firm conclusions and consequently hinders the advancement of the field. Therefore, future studies should ensure that RSHI is clearly defined and distinguishable from concussive impacts. Furthermore, all future studies of RSHI should aim to quantify the impacts, for example, by using sensors.

One of the most common confounding variables identified in this review was the effect of exercise. Notably, S100B was found to be increased in the control groups/conditions of several studies included in this review where exercise was involved [64, 67, 69, 72, 74, 76]. Furthermore, physical exertion and its duration are known to affect serum levels of GFAP and UCH-L1 [125]. Therefore, future biofluid marker RSHI studies need to control for the effects of exercise.

Few studies included in this review were found to examine females and juveniles, while studies including both male and female athletes did not always consider the role of sex. The influence of sex on neurobiology and neurophysiology is largely recognized, and several lines of evidence confirm sex differences in biomarker levels that must be accounted for [126–128]. Therefore, future studies in RSHI and biofluid markers should consider sex differences. Age is also an important covariate that should be controlled. Indeed, studies of juvenile cohorts are limited, and a major knowledge gap remains with regard to how age influences biomarker levels.

One factor that can reduce the heterogeneity of the studies in the field could also be the way the methodological aspects of sampling are standardized and reported. The uncertainty around the best time to sample following RSHI is an urgent pre-analytical factor that needs to be resolved. Furthermore, methods of sampling blood biomarkers are subject to substantial variation with respect to blood collection, choice and preparation of serum or plasma, storage of samples, and the analytical platforms used [129]. Lack of standardization of such pre-analytical variables often makes it impossible to compare results from different laboratories, and potentially adds to noise within studies. In agreement with McDonald et al. [129], we observed that aspects of these processes were often inconsistently documented and note that addressing such variation remains a key issue for future work.

With regard to the current uncertainty about optimal sampling time following exposure to RSHI, marker-specific factors such as half-life should be considered. While it is presently unknown when to sample following RSHI, it may be possible to initially use the marker-specific temporal trends following concussion as a frame of reference. We note, however, that critical RSHI-specific information will rely on researching individual markers’ RSHI-specific response and temporal profile, where possible, sampling at multiple timepoints following RSHI exposure to define the RSHI-specific response and temporal profile of individual markers. Evidence from studies in TBI suggests that temporal profiles of biomarkers are important, and specifically that late biomarker elevation may signal progressive neurological disease [29]. Long-term longitudinal studies in RSHI are needed to address this issue.

There is a need for novel markers capable of providing insight into the pathobiology and pathogenetic mechanisms and demonstrating the link with neurodegeneration (e.g., CTE) [130]. The assessment of circulating levels of P-tau181 and P-tau217 [131], and markers reflecting changes in baseline cerebral physiology and metabolism [132, 133], would be instrumental for the accurate characterization of cerebral health and are therefore a critical avenue for future investigation. Both with regard to novel markers and established markers, we need to understand the mechanisms and understand the link with neurodegeneration. Multimodal studies have a critical role to play, however, in this review, we identified a limited number of studies using multiple methods to date. Therefore, it is recommended that future studies combine biofluid markers with other methods that can reveal the mechanisms of pathology following RSHI exposure, such as combining neuro-imaging and sensitive and informative measures of cognition and motor control [18], and that imaging methods are multi-modal (e.g., [134, 135]).

### Conclusion

In this first review dedicated to systematically scoping the evidence of biofluid marker levels following RSHI exposure, a considerable number of studies were identified. Nevertheless, biofluid marker RSHI research was found to be in its early stages. Presently, the field is overwhelmingly heterogeneous, and the available studies suffer from specific methodological weaknesses. Through systematic scoping of the current evidence, however, we could determine specific ways in which the quality of future studies can be improved. Improving the quality of future research is necessary to assess the utility of under-explored markers as well as those markers that currently appear to show promise. In the meantime, despite the limitations and quality of the current evidence base, the fact that increased levels of brain injury markers were found in biofluids following RSHI exposure warrants caution over the safety of routine RSHI exposure.

### Supplementary Information

**Additional file 1.** Supplementary Materials.

## Data Availability

Extracted data are available at https://osf.io/kd4wn/.

## Abbreviations

BDNF: Brain-derived neurotrophic factor
CNS: Central nervous system CSF: cerebrospinal fluid
CTE: Chronic traumatic encephalopathy
EVs: Extracellular vesicles
GFAP: Glial fibrillary acidic protein
miRNAs: MicroRNAs
NfL: Neurofilament light
NSE: Neuron-specific enolase
PECOS: Population, exposure, comparator, outcomes and study design
PRISMA-ScR: Preferred reporting items for systematic reviews and meta-analyses extension for scoping reviews
p-tau: Phosphorylated tau
ROBINS-I: Risk of bias in non-randomized studies of interventions
RSHI: Repetitive subconcussive head impacts
S100B: S100 calcium-binding protein beta
SST: Subconcussion-specific tool
TBI: Traumatic brain injury
UCH-L1: Ubiquitin C-terminal hydrolase L1
