# Supplementary material for: The Use of Biofluid Markers to Evaluate the Consequences of Sport-Related Subconcussive Head Impact Exposure: A Scoping Review
Source: Sports Med Open. 2024 Jan 25;10:12. doi: 10.1186/s40798-023-00665-6 (PMC10811313; doi:10.1186/s40798-023-00665-6)
Supplement: Supplementary file 1 — Additional file 1. Supplementary Materials. [file 40798_2023_665_MOESM1_ESM.docx]

**The use of biofluid markers to evaluate the consequences of sport-related subconcussive head impact exposure: a scoping review**

Journal: *Sports Medicine – Open*

Liivia-Mari Lember *^,1^, Michail Ntikas *^,1,2^, Stefania Mondello ^3^, Lindsay Wilson ^1^, Thomas G Di Virgilio ^4^, Angus M Hunter ^4,5^, Firas Kobeissy ^6^, Yehia Mchref  ^7^, David I Donaldson ^8^ and Magdalena Ietswaart ^1^

* Joint first authors

1 Department of Psychology, University of Stirling Faculty of Natural Sciences, Stirling, UK

2 The School of Psychology, University of Aberdeen, Aberdeen, UK

3 Biomedical and Dental Sciences and Morphofunctional Imaging, University of Messina Faculty of Medicine and Surgery, Messina, Italy

4 Physiology Exercise and Nutrition Research Group, University of Stirling Faculty of Health Sciences and Sport, Stirling, UK

5 Department of Sports Science, Nottingham Trent University, Nottingham, UK

6 Center for Neurotrauma, Multiomics & amp; Biomarkers, Department of Neurobiology and Neuroscience Institute, Morehouse School of Medicine (MSM), Atlanta, GA 30310, USA

7 Department of Chemistry and Biochemistry, Texas Tech University, Lubbock, TX, USA

8 School of Psychology and Neuroscience, University of St Andrews, St. Andrews, UK

**Corresponding author:** Magdalena Ietswaart; magdalena.ietswaart@stir.ac.uk

Table of Contents

[Table S1 3](#_Toc149253237)

[Table S2 4](#_Toc149253238)

[Table S3 5](#_Toc149253251)

[Table S4 8](#_Toc149253252)

[Table S5 37](#_Toc149253253)

[Table S6 3](#_Toc149253254)8

[Table S7 4](#_Toc149253255)2

[References 4](#_Toc149253256)5

Table S1 Search strategies for each database with search dates

| **Database** | **Search date(s)** | **Search strategy** |
| --- | --- | --- |
| Cochrane Library | 13^th^ of October 2020  Update: 30^th^ March 2022 | #1 MeSH descriptor: [Brain Injuries] explode all trees  #2 MeSH descriptor: [Craniocerebral Trauma] explode all trees  #3 (subconcussi* or brain* or head* or injur* or trauma* or impact* or *TBI or concussi*):ti,ab,kw (Word variations have been searched)  #4 #1 OR #2 OR #3  #5 MeSH descriptor: [Biomarkers] explode all trees  #6 MeSH descriptor: [Blood] explode all trees  #7 MeSH descriptor: [Serum] explode all trees  #8 MeSH descriptor: [Plasma] explode all trees  #9 MeSH descriptor: [Cerebrospinal Fluid] explode all trees  #10 MeSH descriptor: [Urine] explode all trees  #11 MeSH descriptor: [Saliva] explode all trees  #12 MeSH descriptor: [MicroRNAs] explode all trees  #13 MeSH descriptor: [S100 Calcium Binding Protein beta Subunit] explode all trees  #14 MeSH descriptor: [Neurofilament Proteins] explode all trees  #15 MeSH descriptor: [Phosphopyruvate Hydratase] explode all trees  #16 MeSH descriptor: [Glial Fibrillary Acidic Protein] explode all trees  #17 MeSH descriptor: [tau Proteins] explode all trees  #18 MeSH descriptor: [Ubiquitin Thiolesterase] explode all trees  #19 (biomarker* or marker* or blood or cytokine* or coagulation or serum or plasma or saliva or urine or "cerebrospinal fluid" or CSF or neurofilament or NFL or enolase or NSE or tau or "glial fibrillary acidic protein" or "glial intermediate filament protein" or GFAP or "GFA-protein" or "glial protein" or astroprotein* or S100* or S-100* or microRNA or miRNA or UCH-L1 or UCHL1 or ubiquitin*):ti,ab,kw (Word variations have been searched)  #20 #5 OR #6 OR #7 OR #8 OR #9 OR #10 OR #11 OR #12 OR #13 OR #14 OR #15 OR #16 OR #17 OR #18 OR #19  #21 MeSH descriptor: [Football] explode all trees  #22 MeSH descriptor: [Soccer] explode all trees  #23 MeSH descriptor: [Boxing] explode all trees  #24 MeSH descriptor: [Hockey] explode all trees  #25 (collision NEAR/1 sport* or contact NEAR/1 sport* or soccer or football or rugby or boxing or sparring or *hockey):ti,ab,kw (Word variations have been searched)  #26 #21 OR #22 OR #23 OR #24 OR #25  #27 #4 AND #20 AND #26 in Trials |
| CINAHL Complete  Medline EBSCO  PsycINFO  SPORTDiscus | 1937 to 12^th^ October 2020*  1809 to 12^th^ October 2020*  1887 to 12^th^ October 2020*  1892 to 12^th^ October 2020*  *Update: 30^th^ March 2022 | S1 brain* OR head* OR subconcussi* OR injur* OR trauma* OR impact* OR TBI OR mTBI OR concussi*Expanders - Apply related words; Apply equivalent subjects  S2 ( biomarker* OR marker* ) OR ( blood OR serum OR plasma ) OR ( cytokine* OR coagulation ) OR ( urine OR saliva ) OR ( "cerebrospinal fluid" OR CSF ) OR ( S100* OR S-100* ) OR tau OR ( "glial fibrillary acidic protein" OR GFAP OR astroprotein* OR "glial protein" OR GFA-protein* OR "glial intermediate filament protein" ) OR ( enolase OR NSE ) OR ( microRNA* OR miRNA* ) OR ( neurofilament OR NFL ) OR ( ubiquitin* OR UCH-L1 OR UCHL1 ) Expanders - Apply related words; Apply equivalent subjects  S3 ( (collision OR contact) N1 sport* ) OR soccer OR football OR rugby OR boxing OR sparr* OR hockey Expanders - Apply related words; Apply equivalent subjects  S4 S1 AND S2 AND S3 Expanders - Apply equivalent subjects    CINAHL Complete – including only academic journals, dissertations PsycINFO – including only academic journals, dissertations SPORTDiscus – including only academic journals, reports, dissertations |
| Scopus | 1788 to 13^th^ October 2020  Update: 30^th^ March 2022 | ( TITLE-ABS-KEY ( brain* OR head* OR injur* OR trauma* OR impact* OR mtbi OR tbi OR concussi* OR sub-concussi* OR subconcussi* ) ) AND ( TITLE-ABS-KEY ( biomarker* OR marker* OR cytokine* OR coagulation OR blood OR serum OR plasma OR "cerebrospinal fluid" OR saliva OR urine OR csf OR neurofilament OR nfl OR enolase OR nse OR tau OR "glial fibrillary acidic protein" OR "glial intermediate filament protein" OR gfap OR gfa-protein* OR "glial protein" OR astroprotein* OR s100* OR s-100* OR microrna* OR mirna* OR ubiquitin* OR uch-l1 OR uchl1 ) ) AND ( TITLE-ABS-KEY ( ( collision W/1 sport* ) OR ( contact W/1 sport* ) OR soccer OR football OR rugby OR boxing OR sparr* OR *hockey ) ) |
| ClinicalTrials.gov | 13^th^ of October 2020  Update: 30^th^ March 2022 | (subconcussive OR mTBI OR concussion OR impact OR injury) AND (biomarker OR neurofilament OR s100b OR glial OR enolase OR tau OR miRNA OR ubiquitin) AND (soccer OR football OR rugby OR hockey OR sparring OR "collision sports" OR "contact sports") |
| WHO International Clinical Trials Registry Platform | 13^th^ of October 2020: database not working due to COVID-19.  Update: 30^th^ March 2022 | Title: biomarker OR marker OR cytokine OR coagulation OR blood OR serum OR plasma OR "cerebrospinal fluid" OR saliva OR urine OR csf OR neurofilament OR nfl OR enolase OR nse OR tau OR "glial fibrillary acidic protein" OR "glial intermediate filament protein" OR astroprotein OR gfap OR GFA-protein OR "glial protein" OR s100 OR s-100 OR microrna OR mirna OR ubiquitin OR UCHL1 OR UCH-L1  Condition: subconcuss OR mTBI OR concuss OR impact OR injury OR sub-concuss OR mTBI OR head OR brain  Intervention: soccer OR football OR rugby OR hockey OR sparring OR "collision sports" OR "contact sports"  Connected by ‘AND’  Recruiting status: All |
| OpenGrey | 13^th^ of October 2020  Update: 30^th^ March 2022 (database closed down). | (brain* OR head* OR injur* OR trauma* OR impact* OR mtbi OR tbi OR concussi* OR sub-concussi* OR subconcussi*) AND (biomarker* OR marker* OR cytokine* OR coagulation OR blood OR serum OR plasma OR "cerebrospinal fluid" OR saliva OR urine OR csf OR neurofilament OR nfl OR enolase OR nse OR tau OR "glial fibrillary acidic protein" OR "glial intermediate filament protein" OR astroprotein OR gfap OR GFA-protein OR "glial protein" OR s100* OR s-100* OR microrna* OR mirna* OR ubiquitin* OR UCHL1 OR UCH-L1) AND ((collision NEAR/1 sport*) OR (contact NEAR/1 sport*) OR soccer OR football OR rugby OR boxing OR sparr* OR *hockey) |

Table S2 Participants, Exposure, Comparisons, Outcomes, Study design (PECOS) criteria used for inclusion of studies in the review

| PECOS criterion | Description |
| --- | --- |
| Participants | Active or retired male and female contact sport players (including but not limited to American football, rugby, ice-hockey, soccer and boxing) of any age and player level. |
| Exposure | Acute or chronic exposure to RSHI. Those impacts may be a result of either a direct head impact acquired through, for example, soccer heading, sparring and head-to-body collisions, or indirectly through full-body collisions between players or between player and object. |
| Comparisons | All possible comparisons; studies with within and between groups/conditions designs are acceptable, as well as any type of control groups/conditions such as static or exercise-based control groups. Studies without control groups/conditions, as well as comparisons between exposure to high versus low number of impacts are also be included in this scoping review. |
| Outcomes | The concentrations of biofluid markers following acute or chronic exposure to RSHI across groups/conditions and, where reported, the differences between the concentrations pre-to-post RSHI serve as outcome measures. |
| Study design | No restrictions on study design. In addition, all relevant settings are accepted (e.g., field and lab-based studies). |

Table S3 Studies excluded (n=60) during full-text screening with exclusion reasons

| **Citation** | **Exclusion reason** | **Arbitration by a third judge** |
| --- | --- | --- |
| Alosco et al. 2017^1^ | Duplicate | No |
| Alosco et al. 2019^2^ | Redundant publication | No |
| Alosco et al. 2021^3^ | Protocol/registration (no results) | No |
| Alpay 2016^4^ | Not assessing subconcussive head impacts | No |
| Asken et al. 2018^5^ | Duplicate | No |
| Bazarian et al. 2014^6^ | Not assessing biofluid marker(s) in relation to subconcussive impact exposure | No |
| Begum et al. 2020^7^ | Not assessing subconcussive head impacts | No |
| Bernick et al. 2020^8^ | Redundant publication | No |
| Bevilacqua et al. 2019^9^ | Redundant publication | No |
| Brayne et al. 1982^10^ | Duplicate | No |
| Casson et al. 2014^11^ | Not assessing biofluid marker(s) in relation to subconcussive impact exposure | No |
| Chen et al. 2020^12^ | Duplicate | No |
| Daisy et al. 2022^13^ | Not assessing subconcussive head impacts | No |
| Di Pietro et al. 2018^14^ | Not assessing subconcussive head impacts | No |
| Di Pietro et al. 2021^15^ | Not assessing subconcussive head impacts | No |
| Espana et al. 2017^16^ | Not assessing subconcussive head impacts | No |
| Gill et al. 2017^17^ | Not assessing subconcussive head impacts | No |
| Hirad et al. 2019^18^ | Not assessing subconcussive head impacts | No |
| Huibregtse et al. 2020^19^ | Not assessing subconcussive head impacts | No |
| Johnston et al. 2013^20^ | Not assessing subconcussive head impacts | No |
| Joseph et al. 2019^21^ | Duplicate | No |
| National Library of Medicine [NLM], NCT03488381^22^ | Protocol/registration (publications linked to the protocol were duplicates) | No |
| NML, NCT04796207^23^ | Protocol/registration (no results) | No |
| NML, NCT04810130^24^ | Protocol/registration (no results) | No |
| NML, NCT05236127^25^ | Protocol/registration (no results) | No |
| Kawata et al. 2020^26^ | Not assessing biofluid marker(s) in relation to subconcussive impact exposure | No |
| Kilianski et al. 2017^27^ | Not assessing subconcussive head impacts | No |
| Kilic et al. 2019^28^ | Not assessing subconcussive head impacts | No |
| Kochsiek et al. 2021^29^ | Not assessing biofluid marker(s) in relation to subconcussive impact exposure | No |
| Koerte et al. 2022^30^ | Protocol/registration (no results) | No |
| Kokjohn et al. 2013^31^ | Post-mortem | No |
| Koziol et al. 2020^32^ | Not assessing subconcussive head impacts | No |
| Machan et al. 2022^33^ | Not assessing subconcussive head impacts | No |
| McCrea et al. 2020^34^ | Not assessing subconcussive head impacts | No |
| McDonald et al. 2021^35^ | Not assessing subconcussive head impacts | No |
| McLellan et al. 2011^36^ | Not assessing subconcussive head impacts | No |
| McLellan et al. 2011^36^ | Duplicate | No |
| Meier et al. 2017^37^ | Not assessing subconcussive head impacts | No |
| Meier et al. 2017^38^ | Redundant publication | No |
| Meier et al. 2020^39^ | Not assessing subconcussive head impacts | No |
| Meier et al. 2020^40^ | Not assessing subconcussive head impacts | No |
| Meier et el. 2021^41^ | Not assessing subconcussive head impacts | No |
| Meier et al. 2022^42^ | Not assessing subconcussive head impacts | No |
| Neselius et al. 2014^43^ | Redundant publication | No |
| Nitta et al. 2019^44^ | Not assessing subconcussive head impacts | No |
| Oliver et al. 2016^45^ | Redundant publication | No |
| Oliver et al. 2019^46^ | Duplicate | No |
| Otto et al. 2000^47^ | Duplicate | No |
| Owens et al. 2021^48^ | Not assessing subconcussive head impacts | No |
| Oztasyonar 2017^49^ | Duplicate | No |
| Papa et al. 2021^50^ | Redundant publication | No |
| Pin et al. 2021^51^ | Duplicate | No |
| Rogatzki et al. 2021^52^ | Not assessing subconcussive head impacts | No |
| Vike et al. 2022^53^ | Redundant publication | No |
| Vike et al. 2022^53^ | Duplicate | No |
| Shahim et al. 2015^54^ | Not assessing subconcussive head impacts | No |
| Siman et al. 2015^55^ | Not assessing subconcussive head impacts | No |
| Stalnacke and Sojka 2008^56^ | Duplicate | No |
| Tanriverdi et al. 2013^57^ | Redundant publication | No |
| Zetterberg et al. 2006^58^ | Duplicate | No |

Criterion meaning: (1) duplicate – identical publication to one that was assessed for inclusion in the review; (2) not assessing biofluid marker(s) in relation to subconcussive impact exposure – publication assessing the relationship between biofluid markers and other outcome variables (i.e., imaging); (3) not assessing subconcussive head impacts – investigation of e.g., the effects of exercise, peripheral impacts or concussion; (4) post-mortem – investigation of deceased athletes; (5) protocol/registration – registration of trials that were either linked to duplicates or had no results available; (6) redundant publication – publication containing same data (cohort and sample) as in another report that was assessed for inclusion in the review

Table S4 General table including all studies

| **Citation** | **Aim** | **Study type** | **Design** | **Setting** | **Sport** | **Athletes** | **Controls** | **Exposure** | **Biomarkers and source** | **Sample times** | **Findings** |
| --- | --- | --- | --- | --- | --- | --- | --- | --- | --- | --- | --- |
| Akkurt et al. 2020^59^ | Investigate the effects of heading on pituitary function in retired soccer players. | Chronic | Cross sectional study | Other | Soccer | 32 retired professional players, aged 43.4 ± 5.5 | 26 healthy sedentary males, aged 43.3 ± 6.4 | Soccer participation | Basal: fT3, fT4, TSH, PRL, FSH, LH, t-T, IGF-I, ACTH, insulin; peak: C, GH Source: blood | N/A | No significant findings. |
| Alosco et al. 2017^60^ | Examine t-tau concentrations in former NFL players presumably at risk for CTE, compared with same-age controls. | Chronic | Cross sectional study | Other | Am. football | 96 male symptomatic former NFL players, aged 55.2 ± 7.9 | 25 asymptomatic controls w/o contact sport history, aged 57.0 ± 6.6 | Am. football participation (CHII) | Plasma t-tau | N/A | No significant difference between groups. T-tau correlated significantly with CHII. |
| Alosco et al. 2018^61^ | Compare biomarkers between symptomatic former NFL players and same-age asymptomatic controls and examine the relationship between estimated exposure to RHI and biomarkers. | Chronic | Cross sectional study | Other | Am. football | 68 male symptomatic former NFL players, aged 54.4 ± 8.0 | 21 asymptomatic controls w/o contact sport history, aged 57.6 ± 7.1 | Am. football participation (CHII) | CSF: t-tau, p-tau181, Aβ1-42, sTREM2 | N/A | No differences between players and controls. Significant association between CHII and t-tau. |
| Antonio et al. 2021^62^ | Assess NfL concentrations in Division II female soccer players vs highly trained female controls that did not participate in a contact sport. | Chronic | Cross sectional study | Other | Soccer | 8 female Division II soccer players, aged 22 ± 6 | 17 female non-contact sport athletes, aged 25 ± 8 | Soccer participation | Plasma NfL | N/A | NfL levels were significantly higher in soccer players than in controls. |
| Arslan et al. 2010^63^ | Examine serum markers of brain tissue damage following a single match of Free and Greco-Roman style wrestling. | Acute | Cohort study | Field | Wrestling | 15 male Greco-Roman wrestlers, median age (range) 19.0 (19-30); 16 male Free style wrestlers, 20.0 (19-26) | N/A | Wrestling competition (3 x 2 min) | Serum: S100B, H-FABP | Before and 20 min post | S100B and H-FABP significantly increased in both groups. |
| Asken et al. 2018^5^ | Examine the effect of cumulative exposure to collision sports on serum biomarker concentrations. | Chronic | Observational cohort study | Other | Mix of contact sports (football, soccer, diving, wrestling, ice-hockey, Am. football) | 415 (256 M, 159 F) collegiate athletes, aged 19.0 ± 1.2 | N/A | Cumulative exposure to collision sports in yr (and modified CHII) | Serum: S100B, t-tau, Aβ42, UCH-L1, GFAP, CNPase, MAP2 | Off-season | No significant findings. |
| Austin et al. 2021^64^ | Investigate if heading dose and impact biomechanics affect NfL levels. | Acute | Randomised controlled trial | Lab | Soccer | 36 males (12 in each heading group), aged 23.7 ± 4.8 | 8 males, aged 23.7 ± 4.8 | 10, 20 and 40 linear headers | Serum NfL | Baseline and 6 h, 24 h and 7 days post | No significant findings. |
| Bamaç et al. 2011^65^ | Investigate the relationship between expression of two neurotrophins (NGF, BDNF) and repeated head impacts. | Acute | Non-randomised experimental study | Lab | Soccer | 17 male professional soccer players, aged 24.6 ± 4.4 | N/A | 15 jumping headers; headed from a corner kick (launch distance of 30-35 m) | Serum: BDNF, NGF | Before and after | NGF and BDNF levels increased significantly. |
| Bernick et al. 2018^66^ | Evaluate longitudinal change in NfL and tau in professional fighters. | Acute, semi-acute & chronic | Longitudinal cohort study | Other | Boxing, MMA | 52 (50 M, 2 F) retired professional boxers, aged 48.0 ± 10.3; 117 (110 M, 7 F) active professional boxers, aged 30.4 ± 6.9; 169 (152 M, 17 F) active professional MMA athletes, aged 29.6 ± 4.8 | 79 (69 M, 10 F) controls w/o contact sport history, aged 30.8 ± 10.0 | Fights and sparring (martial arts or boxing) | Plasma: NfL, tau | Baseline and ≥2 measurements over 1.6 years (average) (range: 1-5 years); active fighters: ≥45 days from a sanctioned fight | Active boxers had the highest baseline NfL levels. Number of sparring rounds completed by the active fighters within 2 weeks correlated with NfL, but not with tau. Average yearly (n=126) percentage change in tau across groups was significant, with active MMA fighters having greater increases in tau levels than controls. |
| Bouvier et al. 2016^67^ | Investigate the utility of S100B for the determination of concussion in a professional rugby team. | Acute | Prospective cohort study | Field | Rugby | 39 professional rugby players, aged 28.6 ± 3.98 (range: 22-37) (27 non-concussed, 5 concussed) | N/A | Rugby match (collisions) | Serum S100B | 3 basal levels during the season (>48 h from competition) and within 2 and 36 h after a match | S100B was significantly higher immediately post compared to basal and 36 h post levels (n=27). Number of collisions correlated with S100B levels. No significant difference in S100B levels between concussed and non-concussed players. |
| Brayne et al. 1982^10^ | Compare CK-BB before and after boxing and track cycle racing. | Acute | Cohort study | Field | Boxing | 16 amateur boxers, aged 16-25 (mean: 20.3) | 16 track racing cyclists, aged 17-25 (mean: 22.6) | 3 x 3-min boxing rounds; control: 40 mile race | CK-BB (blood) | 1 h before and 10-30 min post | CK-BB increased more in boxers than in cyclists (p <0. 01). CK-BB leves significantly correlated with the number of blows to the head. |
| Di Battista et al. 2016^68^ | Examine a panel of systemic brain injury markers and inflammatory mediators in relation collision sport participation. | Chronic | Cross sectional study | Other | Mix of contact sports (men's ice-hockey, football, rugby, lacrosse, women's rugby) | 41 (39 M, 2 F) collision sport athletes; aged (including all participants): M (n=60) 19.5 ± 2.0, F (n=27) 19.5 ± 1.8 | 46 (21M, 25F) non collision sport athletes (inadvertent contact: soccer, basketball) | Collision sport participation | Neuroinjury markers: S100B, GFAP, NSE, tau, CK-BB, BDNF, neurogranin, VILIP-1, vWF, PRDX-6; cytokines: IL-1a, -1b, -2, -4, -5, -6, -7, -10, -12p40, -12p70, -13, -15, -16, -17A, TNF-α, -β, GM-CSF, VEGF, IFN-γ; chemokines: eotaxin, eotaxin-3, IP -10, IL-8, MCP-1, -4, MDC, MIP-1α, -1β, TARC (plasma) | Before the start of varsity season | Collision sport participation was associated with increases in tau in males. |
| Dorminy et al. 2015^6970^ | Examine the effect of soccer heading ball speed on S-100B, concussion side-line assessments and linear head impact acceleration. | Acute | Randomised controlled trial | Lab | Soccer | 16 (10 M, 6 F), aged 20.4 ± 0.2 (30 mph n= 5, 40 mph n= 6, 50 mph n= 5) | N/A | 5 linear standing headers | Serum S100B | Before and 1-1.5 h post | No significant findings. |
| Graham et al. 2011^70^ | Investigate if punches to the head (PTH), sustained during a boxing bout result in elevated levels of neurochemical markers (compared to punches to the body (PTB)). | Acute | Retrospective cohort study | Field | Boxing | 8 male amateur boxers, aged 17.6 ± 5.3 (PTH – punches to the head and body) | 8 male amateur boxers, age 19.1 ± 3.2 (PTB – punches to the body) | 5 x 2-min boxing rounds | Serum: S100B, NSE, C | 1 h before and after 5 min of cessastion | NSE, S100B, and C increased in the PTH group (p<0.05), but not in the PTB group. S100B and NSE strongly correlated with the number of PTH (r^2^=0.43 and 0.25, respectively). |
| Graham et al. 2015^71^ | Assess if kicks to the head (KTH) compared to kicks to the body (KTB) in a karate contest result in elevated levels of neurochemical markers indicating cerebral damage. | Acute | Cohort study | Field | Karate | 12 males, aged 30.4 ± 6.7 (KTH – kicks to the head and body) | 12 males, aged 28.2 ± 6.5 (KTB – kicks to the body) | 4 x 3-min karate round | Serum: S100B, NSE | Before and immediately after | S100B and NSE increased in the KTH group (p<0.05), but not in the KTB group. S100B was significantly increased in KTH vs KTB group, but not NSE. |
| Heileson et al. 2021^72^ | Examine the effect of supplemental ω-3 fatty acids on changes in NfL, and fatty acids over the course of a competitive season in Am. football athletes. | Semi-acute | Non-randomised experimental study | Field | Am. football | 66 male NCAA Am. football players (31 in supplement group) | N/A | Am. football games and practices | Serum NfL | Baseline: following >14 week period of non-contact, after pre-season camp and throughout season. | The no treatment group exhibited a significant increase in NfL, no change in the supplement group. Serum NfL remained elevated in athletes not receiving supplementation throughout the season, change in NfL in the treatment group not significant. |
| Hicks et al. 2021^73^ | Determine whether 40 miRNAs previously implicated in concussion pathophysiology are affected by participation in a variety of contact and non-contact sports. | Acute & semi-acute | Case control study | Field | Acute: soccer, Am. football; semi-acute: lacrosse, basketball, hockey, soccer, mixed martial arts. | Acute: 32 in total; 12 semi-professional soccer players, 20 collegiate footballers. Semi-acute: 106 athletes (83 M, 23 F), aged 23 ± 5 | Acute: 51 collegiate non contact sport athletes (runners, rowers); semi-acute: N/A | Acute: full-contact soccer scrimmage, full-contact football practice. Semi-acute: season of contact sport participation. | 40 miRNAs (saliva) | Acute: before and <20 min post; semi-acute: pre and post season | Acute: 1 miRNA (miR-4510) increased only among contact sport athletes, no relationship with impacts. Semi-acute: 23 miRNAs changed at post season, 2 of these (miR-26b-3p, miR-29c-3p) were associated with the number of head impacts. |
| Hoffman et al. 2022^74^ | Examine the effect of an Am. football game on changes in cognitive function and circulating concentrations of BDNF and blood markers of both brain and systemic inflammation. | Acute | Cohort study | Field | Am. football | 15 Israel national football team players, aged 26.2 ± 5.3 (range: 18-35) | N/A | Am. football match | Serum: BDNF, CRP, GFAP, S100B, tau. Plasma: IL-4, IL-6, IL-8, IL-10, TNF-α | 1 week before, immediately (<30min) and 24 h post | BDNF and CRP increased significnatly at 24 h post. TNF-α increased significantly immediatly post and returned to baseline levels at 24 h. |
| Horner et al. 1993^75^ | Compare biomarker response to noncontact and contact sport. | Acute | Cohort study | Field (control group: lab) | Boxing | 8 male Olympic boxers, age range: 18-28 | 17 male amateur oarsmen, age range: 18-23 | 3 x 3 min boxing rounds; controls: 6 min ergometer test | Serum: CK-BB, NSE | Before and after | NSE and CK-BB significantly increased after boxing. |
| Huibregtse et al. 2020a^76^ | Assess changes in S100B following heading and kicking (control group). | Acute | Randomised controlled trial | Lab | Soccer | 37 (19 M, 18 F) soccer players, median age (IQR) 21 (19-22) | 31 (14 M, 17 F) soccer players, median age (IQR) 21 (20-22) | 10 linear headers; controls: 10 kicks | Plasma S100B | Before and 0, 2 and 24 h post | No significant differences between groups at any time points. Significant increase at 24 h post heading. |
| Huibregtse et al. 2020b^77^ | Examine changes in inflammatory markers after 10 controlled soccer headers and kicking (control group). | Acute | Randomised controlled trial | Lab | Soccer | 22 (9 M, 13 F) soccer players, aged 20.1 ± 1.5 | 17 (7 M, 10 F) soccer players, aged 21.5 ± 1.8 | 10 linear headers; controls: 10 kicks | Plasma: CCL11, CCL2, IL-10 | Before and 0, 2 and 24 h post | No significant between-group differences. |
| Joseph et al. 2018^21^ | Determine if high-acceleration head impacts (HHIs) that do not result in mTBI lead to increases in biomarkers and observe biomarkers over a football season. | Acute & semi-acute | Prospective observational cohort study | Field | Am. football | 16 male high-school varsity footballers, aged 16.9 ± 0.2 (HHI group n=6, non-HHI group n=5, pre and post season testing n=12) | N/A | Am. football games, practices and season | Serum: NfL, tau, GFAP, SBDPs, UCH-L1 | Semi-acute: pre and post season; acute: 1-2 h post | Acute: tau and UCH-L1 increased more in the HHI group than in the non-HHI. No significant changes in NfL, GFAP, or SBDPs. Semi-acute: tau and UCH-L1 increased significantly pre to post season, no changes in NfL, GFAP or SBDPs. |
| Kawata 2016^78^ | Investigate the acute and longer-term effects of repetitive subconcussive impacts on biomarker levels. | Acute & semi-acute | Prospective longitudinal cohort study | Field | Am. football | 22 male Division I collegiate footballers, aged 20.6 ± 1.5 | N/A | Pre-season Am. football practices; season of Am. football | Plasma: S100B (S100B - pre and post season data only), PINCH | Pre and post season, and before and after 5 practices (1 non-contact, 4 full contact) | Acute: PINCH increased post practice. No correlations between PINCH and impact metrics. Semi-acute: PINCH increased from baseline to post season, but S100B did not. |
| Kawata et al. 2017^79^ | Investigate if repetitive subconcussive impacts cause changes in S100B and tests the associations between S100B and impacts metrics. | Acute | Prospective longitudinal cohort study | Field | Am. football | 22 male Division I collegiate footballers, aged 20.6 ± 1.5 | N/A | Pre-season Am. football practices | Plasma S100B | Baseline, before and after 5 pre-season practices (1 non-contact, 4 full contact) | S100B significantly increased following all practices. Impact metrics were associated with greater pre-post practice increases in S100B. |
| Kawata et al. 2018a^80^ | Examine tau protein and subconcussive impact kinematic data in collegiate football players during pre-season practices. | Acute | Prospective longitudinal cohort study | Field | Am. football | 23 male Division I collegiate footballers, aged 20.5 ± 1.3 | N/A | Pre-season Am. football practices | Plasma tau | Pre season baseline, immediately before and ≤1 h after 4 practices (1 non-contact) | Tau significantly increased after all practices. Subconcussive impact metrics did not predict increases in tau. |
| Kawata et al. 2018b^81^ | Assess neural burden of a single hockey season. | Semi-acute | Cohort study | Field | Ice-hockey | 8 male professional players (including 2 concussed athletes), aged 26.6 ± 1.6 | N/A | Ice hockey season | Plasma exosome markers that reacted positive with CD81 and various neuron- and glia-specific antigens. (SNAP25, NfL, tau, SYP, EAAT1, GFAP, OMG, MBP, CD11b, IL-8, TNF-α, CD81) | Pre and post season | Significant increase in [NfL]SNAP25+/[CD81]SNAP25+ at post season (did not withstand Bonferroni correction for multiple comparisons). All other markers failed to reach statistical significance. |
| Kelestimur et al. 2004^82^ | Assess the pituitary functions by basal hormone measurements and GH axis in boxers and age, sex and BMI matched healthy subjects. | Chronic | Cross sectional study | Other | Boxing | 11 male amateur boxers (3 active, 8 retired), aged 38.0 ± 3.6 (range: 18-55) | 7 healthy males (non-boxers), aged 34.4 ± 3.2 (range: 20-52) | Boxing (duration in yr, number of bouts) | Basal: fT3, fT4, TSH, PRL, C, FSH, LH, f-T, t-T, IGF-I, GH; peak: GH (serum) | N/A | Basal hormone levels were in the normal range. Peak GH levels were significantly different between boxers and controls (5 boxers had severe GH deficiency). Peak GH levels correlated negatively with boxing duration and number of bouts. IGF-I was significantly higher in controls. |
| Kelly et al. 2014^83^ | Determine the rate of pituitary hormonal dysfunction in retired NFL players. | Chronic | Cross sectional study | Other | Am. football | 68 male retired NFL players, aged 47.3 ± 10.2 (range: 30-65) | 30 male non-head-injured controls (used for defining BMI adjusted GHD cut points) | NFL career (years, number of games) | Basal: fT4, tT4, TSH, PRL, C, FSH, LH, f-T, t-T, ACTH, IGF-I; peak: GH, C (serum) | N/A | Hormone deficiency in 23.5% athletes: 10 (14.7%) with isolated GHD, 3 (4.4%) with isolated hypogonadism and 3 (4.4%) with both GHD and hypogonadism. |
| Major et al. 2020^84^ | Investigate the pathophysiological consequences of RHI exposure, and examine how biological sex may modify this response. | Chronic | Cross sectional study | Other | Au. football | 81 (50 M, 31 F) amateur footballers (no-mTBI history n=42; mTBI history n=39), aged ~24 | 42 (23 M, 19 F) age-matched non-contact sport athletes | Au. football participation | Serum: UCH-L1, NfL, tau, p-tau, GFAP, BLBP, PEA15, 4-HNE, VEGF-A, vWF, CLDN5, HMGB1, fibrinogen | Pre season | Male footballers had significantly greater VEGF-A levels compared to the controls. Fibrinogen levels correlated with years of collision sport participation in females. |
| Marchi et al. 2013^85^ | Measure S100B in football players following sub-concussive head hits. | Acute & semi-acute | Cohort study | Field | Am. football | Acute: 27 collegiate players, aged ~21; semi-acute: 10 collegiate players | N/A | Am. football matches (acute) and season (semi-acute) | Serum: S100B, S100B Auto-Ab | Acute: baseline (prior to any football related activity), 24 h before, 1 and 24 h post; semi-acute: pre and post season | No significant differences in S100B pre and post game levels. S100B increased (1 h post) only in the presence of frequent sub-concussive head hits. Progressive increase in anti S100B Ab titer in ~50% of the players over the season. |
| Matuk et al. 2021^86^ | Evaluate acute changes in salivary EVs gene expression in subjects before and after a head injury. | Acute & chronic | Cohort study | Field | MMA | 8 (5 M, 3 F) fighters, aged 18-30 | 7 (2 M, 5 F) healthy, non-fighters, aged 22-33 | MMA fight | Saliva EVs gene expression | Before and <1 h post (controls: baseline only) | EV: No differences in size and concentration. Gene expression was significantly different in MMA fighters at baseline. Pre to post fight: ALOX5, ITGB2, and MAPK8 demonstrated highest upregulation. ADRB2 and HRH1 were downregulated. |
| Meier et al. 2016^87^ | Explore the effects of repeated concussive and subconcussive head impacts on the KYN metabolic pathway (and its relationship with hippocampal volume). | Chronic | Cross sectional study | Other | Am. football | 49 male current or former collegiate football athletes: 24 w/o (aged 20.2 ± 1.2) and 25 with history of mTBI (aged 21.0 ± 1.5) | 27 healthy male non-collision sport and non-athlete controls, aged 21.9 ± 2.2 | Am. football participation | Plasma: KYN, 3HK, QUIN, KYNA | N/A | Controls had higher levels of KYN than athletes. Players with mTBI history had higher QUIN than players w/o. |
| Muñoz et al. 2021^88^ | Investigate the miRNA profile in circulating sEVs derived from human plasma following repetitive head impact exposure. | Acute | Randomised controlled trial | Lab | Soccer | 6 (2 M, 4 F) soccer players, aged 23 ± 6 | 12 soccer players: 6 (5 M, 1 F) in leg impact group, aged 22 ± 4 and 6 (4 M, 2 F) in no intervention group, aged 21 ± 2 | 10 linear headers | Plasma sEV miRNAs | Before and 24 h post | No changes in sEVs concentration or size. 3 miRNAs increased more than 4-fold (miR-92b-5p, miR-423-5p, and miR-24-3p), and 4 miRNAs decreased more than 3-fold (miR-7844-5p, miR-144-5p, miR-221-5p, and miR-22-3p) at 24 h post heading. |
| Muraoka et al. 2019^89^ | Identify a potential biomarker for diagnosing and monitoring of CTE. | Chronic | Cross sectional study | Other | Am. football | 15 male symptomatic former NFL players, aged 56.3 ± 7.3 | 16 asymptomatic males w/o contact sport history, aged 57.1 ± 7.0 | Am. football career | CSF: t-tau and p-tau181 in EVs | N/A | No significant difference between former players and controls. |
| Muraoka et al. 2021^90^ | Examine protein profiles of EVs separated from the plasma of former NFL players at risk for CTE. | Chronic | Cross sectional study | Other | Am. football | 27 male symptomatic former NFL players, aged 56.6 ± 7.6 | 25 asymptomatic males w/o contact sport history, aged 57.0 ± 6.6 | Am. football career | Plasma: proteomics, EV t-tau, EV p-tau181 | N/A | Proteomics: 9 proteins were significantly up- and 8 were down-regulated in former players compared to controls. T-tau and p-tau levels were significantly different in former players. |
| Mussack et al. 2003^91^ | Evaluate S100B levels after controlled heading and compare with measurements after normal exercise and minor TBI. | Acute | Non-randomised experimental study | Lab | Soccer | 61 male amateur players, median age (IQR) 15.3 (14.8-16.4) | Exercise-based control group: 58 male amateur players, median age (IQR) 15.9 (15.0 - 16.8); 81 mTBI controls: 20 CCT+, 41.8 (32.3 - 61.1) and 61 CCT-, 37.1 (27.6 - 53.5) | Controlled soccer heading aimed at the forehead performed for a median of 55 minutes; controls: 61 min of exercise. | Serum S100B | Baseline, 1 and 6 h post | No meaningful differences between or within the groups. S100B levels were significantly higher in the TBI groups. |
| Neselius et al. 2012^92^ | Investigate the relationship between Olympic (amateur) boxing and CSF brain injury biomarkers. | Acute & semi-acute | Prospective cohort study | Field | Boxing | 30 (28 M, 2 F) Olympic boxers, mean age (range) 22 (17-34) | 25 (20 M, 5 F) healthy controls, mean age (range) 22 (17-30) | Boxing bout | S100B, NfL, t-tau, p-tau181, GFAP, Aβ1-42, H-FABP | 1-6 days post and after ≥14 days rest | NfL, GFAP, T-tau and S100B were significantly increased after boxing compared to the levels observed in healthy controls. NfL and GFAP remained elevated after rest period. No significant differences between groups for H-FABP, p-tau and Aβ1-42. |
| Neselius et al. 2013a^93^ | Find biomarkers of mTBI and to investigate the role of APOE e4 allele genotype associated with poor outcome after TBI. | Acute & semi-acute | Prospective cohort study | Field | Boxing | 30 (28 M, 2 F) Olympic boxers, mean age (range) 22 (17-34) | 25 (20 M, 5 F) controls, mean age (range) 22 (17-30) | Boxing bout | CSF: pNFH, sAPPα, sAPPβ, ApoE, ApoA1, AβN38, AβN40, AβN42; plasma: Aβ1-42, ApoE genotype | 1-6 days post and after ≥14 days rest | pNFH was significantly increased after boxing compared to controls; concentrations decreased at follow up but were significantly higher compared to controls. No other significant findings. |
| Neselius et al. 2013b^94^ | Investigate if Olympic (amateur) boxing is associated with elevation of brain injury biomarkers when compared to controls. | Acute & semi-acute | Prospective cohort study | Field | Boxing | 30 (28 M, 2 F) amateur boxers (competing at elite level), mean age (range) 22 (17-34) | 25 (20 M, 5 F) healthy controls, mean age (range) 22 (17-30) | Boxing bout | Plasma: tau, Aβ42; serum: GFAP, BDNF, S100B | 1-6 days post and after ≥14 days rest | Tau was significantly increased in the boxers after a bout compared to the levels observed in healthy controls. No significant difference between groups at follow-up. Other brain injury markers did not differ between the groups. |
| Nowak et al. 2022^95^ | Examine the response to subconcussive head impacts in individuals with and without ADHD diagnosis. | Acute | Case control study | Lab | Soccer | 17 (6 M, 11 F) soccer players with ADHD, aged 20.2 ± 0.2; 17 (10 M, 6 F) w/o ADHD, aged 21.1 ± 0.1 | 17 (7 M, 10 F) soccer players with ADHD, aged 20.5 ± 0.1 | 10 linear headers; controls: 10 kicks | Plasma: NfL, tau, GFAP, UCH-L1 | Baseline, 2 and 24 h post | GFAP increased at 2 and 24 h post heading in ADHD group. UCH-L1 increased 24 h post heading in ADHD group. NfL increased 24 h post heading only in the non-ADHD group (~1.2 fold increase). No changes in the control group. |
| Obminski et al. 2009^96^ | Determine hormone levels in amateur boxers following a boxing tournament in relation to their age and total life-time number of boxing matches. | Acute & chronic | Cross sectional study | Field | Boxing | 15 amateur boxers: 11 winners, aged 21.4 ± 1.7; 4 defeated, aged 21.5 ± 1.3 | N/A | 3 round boxing match; total life-time number of boxing matches | Plasma (capillary blood sample from the earlobe): T, GH, C | 3 min post | No significant findings. |
| O'Brien et al. 2021^97^ | Assess the association between years of collision sport participation (surrogate measure of subconcussive impact exposure) with levels of IL-1β band IL-18 in pre-season serum samples. | Chronic | Cross sectional study | Other | Au. football | 105 (65 M, 40 F) amateur athletes, aged M: 23.06 ± 0.67, F: 24.63 ± 1.44 | N/A | Au. football participation | Serum: IL-1β, IL-18 | Pre season | Weak positive correlation between years of collision sport involvement and serum IL-18 levels in males, no association in females. No association between years of collision sport and IL-1β. |
| O'Connell et al. 2018^98^ | Examine the effects of rugby match play and season on S100B compared with age- and fitness-matched non-contact sport athletes. | Acute & semi-acute | Prospective longitudinal cohort study | Field | Rugby | 38 professional male rugby players, aged 26.6 ± 4.4 | 15 rowers, median age (IQR) 22.0 (20.0-24.0) | Rugby games and season | Serum S100B | Pre and post season, ≤2 h post games. Controls: pre and post 80 min of training. | Acute: Significant increase in S100B after rugby games and after training in rowers, with rugby players having significantly higher S100B levels post game compared to rowers post exercise. Semi-acute: No significant findings. |
| O'Keeffe et al. 2020^99^ | Investigate if the integrity of the blood-brain barrier (BBB) is altered in high-risk population for concussions. | Acute & semi-acute | Cohort study | Field | Rugby | 8 rubgy university team players, mean age (range) 22.1 (18-23); 11 male rugby school team players, mean age 17.4 | 27 non-contact sport athletes, median age (range) 28 (18–36); 26 healthy non-athlete controls, median age (range) 30 (18-40) | Rugby match (university team) and season (school and university team) | Plasma: S100B, BDNF, CCL2† | University team: pre season, ≤2 h post match, 2 months post season.  School team: pre and post season. | Semi-acute (school and university sample): BDNF significantly increased and S100B decreased post-season compared to pre-season. Acute (university sample): MCP-1 and S100B significantly increased post-match. |
| Oliver et al. 2016^100^ | Examine concentrations of NfL over the course of a season in collegiate Am. footballers. | Semi-acute | Observational cohort study | Field | Am. football | 116 Division I American footballers (baseline), aged 20 ± 1 (of whom 19 were sampled over the season; 9 non-starters, 11 starters) | 19 male NCAA Division I swimmers, aged 20 ± 1 (baseline sample only) | Am. football season | Serum NfL | T1: after 9 weeks of non-contact; T2: after training camp; T3: following pre-season camp (highest concentration of impacts); T4 through T8 mid-season, 36-48 h post games. | NfL increased significantly over the course of the season in starters. |
| Oliver et al. 2017^101^ | Examine the effect of a season of Am. football on tau. | Semi-acute | Longitudinal observational cohort study | Field | Am. football | 19 (11 starters, 8 non-starters) Division I footballers, aged 20 ± 1 | 19 NCAA swimmers, aged 20 ± 1 (baseline sample only) | Am. football season | Plasma tau | T1: after 9 weeks of non-contact; T2: after training camp; T3: following pre-season camp (highest concentration of impacts); T4 through T8 mid-season, 36-48 h post games. | No significant findings. |
| Oliver et al. 2019^46^ | Examine changes in biomarkers of subconcussive head trauma over the course of an Am. football season. | Semi-acute | Prospective longitudinal cross-sectional study | Field | Am. football | 35 (20 starters, 15 non-starters) Division III footballers, aged 21 ± 1 | N/A | Am. football season | Serum NfL, plasma tau | T1: after 14-weeks of non-contact; T2: end of camp (period with most impacts); T3: 72 h post full-contact practice, T4 and T5: ~ 36 h following a game; T6 and T7: post season. | Tau decreased and NfL increased over the course of the season. |
| Otto et al. 2000^47^ | Assess S100B before and after boxing and in control groups, in which no head trauma was proposed. | Acute | Cohort study | Field (boxing), lab (soccer) | Boxing, soccer | 25 male amateur boxers: competitive fights n=10, sparring fights n=15 (13 with head protector), aged 17-40; heading: 12 sportsmen, aged 20-52 | 35 male runners (sprinters, 10 and 25km), aged 20-52. 12 male cyclists, aged 23-52. | (1) 5 x 2-min competitive boxing rounds (2) 3 or 5 x 2-min sparring fights (3) 20 standing soccer headers (ball dropped from 7.5 m) | Serum S100B | Before and ≤15 min post | S100B increased after boxing and running disciplines but not after ergometer cycling or heading. The number and severity of the head strikes in boxing correlated positively and significantly with S100B. |
| Owens et al. 2021^102^ | To determine the molecular, cerebrovascular and cognitive consequences of contact events during a season of professional rugby. | Semi-acute | Cohort study | Field | Rugby | 21 professional male players (13 forwards, 8 backs), aged 25 ± 4 | N/A | Rugby games and training | Plasma: ascorbate free radical (AFR); bioactive nitric oxide concentration (combined bioactive concentrations of nitrite (NO-2) and S-nitrosothiols (RSNO)) | Pre and post season | Increase in systemic oxidative–nitrosative stress (OXNOS), confirmed by elevated ascorbate free radical and corresponding suppression of nitric oxide bioavailability. |
| Oztasyonar 2017^49^ | Compare BDNF levels between combat sport braches. | Acute | Cohort study | Field | Boxing, Tae Kwon Do (TW) | 20 male boxers, aged 20.2 ± 1.5; 20 male Tae Kwon Do fighters, aged 20.6 ± 1.7 | 20 male runners, aged 19.9 ± 1.6; 20 sedentary participants, aged 20.4 ± 1.9 | Boxing: 3 x 3-min rounds; Tae Kwon Do: 2 x 3-min rounds; controls: 800 m running competition | Serum BDNF | Immediately before and after | (1) Boxers and fighters had high basal BDNF levels; (2) Significantly higher BDNF concentrations post training for all athletes; (3) fighters had significantly higher BDNF levels than other groups. |
| Papa et al. 2019^103^ | Assess miRNA biomarkers in collegiate football players over the playing season. | Semi-acute | Prospective controlled observational cohort study | Field | Am. football | 22 male Division I collegiate football players, mean age 21 (range: 19-24) | 30 (15 M, 15 F) healthy non-athlete controls, mean age 31 (30-35) | Am. football season | Serum miRNAs (miR-20a, miR-505*, miR-362-3p, miR-30d, miR-92a, miR-486, miR-195, miR-9-3p, miR-151-5p) | Before and post season | All the athletes had elevated levels of circulating miRNAs at the beginning and the end of the season compared with controls (p<0.001); miR-505*, miR-486, miR-30d, miR-92a, miR-362-3p and miR-195 increased significantly from pre to post season. |
| Pin et al. 2021^104^ | Identify new candidate autoimmune targets of IgA and test whether concussion and subconcussive impacts increase IgA reactivity and show similarities. | Semi-acute | Observational cohort study | Field | Contact and team sports (including Am. football, lacrosse and soccer). | 62 male and female team sport athletes | 36 male and female team sport athletes | Contact sports participation (season of training and games) | Saliva autoimmune targets of IgA (IgA reactivity) | Pre and post season | Significant increase in the prevalence of IgA toward protein fragments representing 5-hydroxytryptamine receptor 1A (HTR1A), serine/arginine repetitive matrix 4 (SRRM4) and FAS (tumor necrosis factor receptor superfamily member 6) after subconcussive exposure and concussion. |
| Puvenna et al. 2014^105^ | Investigated the effects of sub-CHIs in collegiate football players on markers of BBBD, markers of CSF leakage and markers of brain damage. | Acute | Cohort study | Field | Am. football | 15 athletes | 406 positive controls with mTBI and 465 negative controls | 2 Am. football games | Serum: S100B, UCH-L1, β-2-transferrin | Baseline (day before) and post (≤1 h) (positive controls: ≤6 h of injury) | S100B and UCH-L1 significantly increased after both games. Beta-2 transferrin levels remained unchanged. No correlation between head hits and UCH-L1. |
| Rogatzki et al. 2016^106^ | Determine if the biomarkers of head injury increase following an Am. football game. | Acute | Cohort study | Field | Am. football | 17 male Division III collegiate footballers, aged 19.5 ± 0.9 | N/A | Am. football game | Serum: S100B, NSE | 2 days before and 1 h post | Significant findings. |
| Rogatzki et al. 2018^107^ | Determine if serum S100B increases similarly as a result of playing Am. football compared to exercise alone. | Acute & semi-acute | Cohort study | Field | Am. football | 16 male Division III collegiate footballers, age range 18 to 22 | 32 controls, age range 18-22 [control groups: resistance exercise n=18 (10 M, 8 F); treadmill running n=8 (5 M, 3 F); treadmill walking n=6 (3 M, 3 F)] | Am. football games | Serum: S100B | Baseline (prior to training camp); before (day before, ≤30 min post practice) and ≤30 min post 4 games. Controls: immediately before and ≤30 min post. | Acute: significant findings for experimental and 2 control groups.  Semi-acute: no significant findings.  Number of hits and plays correlated with S100B. |
| Roser et al. 2017^108^ | Evaluate the somatotrope pituitary function in professional soccer players. | Chronic | Cross sectional study | Other | Soccer | 15 male professional soccer players, aged 31.1 ± 9.6 (range: 21-50) | N/A | Soccer participation | Basal: fT3, fT4, TSH, C, FSH, LH, T, IGF-I, GH; peak: GH, C (blood) | N/A | No significant findings. |
| Rubin et al. 2019^109^ | Investigate if repetitive subconcussive impacts cause changes in NfL levels, and test associations between NfL levels and impact metrics. | Acute | Cohort study | Field | Am. football | 18 Division I college footballers, median age (IQR) 20.5 (20-22) | N/A | Pre-season Am. football practices | Plasma NfL | Baseline: 2 months prior to any practices; <1 h before and <1 h post practices | Number of hits, PLA, PRA, and HIC were significantly associated with pre-to post-practice changes in NfL. |
| Sandmo et al. 2020^110^ | Explore if repetitive headers or accidental head impacts in soccer could cause short-term structural brain damage and assess potential long-term effects. | Acute & chronic | Prospective cohort study | Field | Soccer | Male premier league players: heading exercise group n=47, head impacts during a match n=35 | Male premier league players: high intensity exercise n=47 | (1) heading exercise (90 min), (2) head impacts (some concussive) during match play | Serum: NfL, tau | Baseline, 1 and 12 h post | NfL levels were unaffected. Tau rose significantly 1 h after high-intensity exercise and after repetitive headers but not after accidental head-impacts during a match. |
| Sandmo et al. 2022^111^ | Explore the short-term effects of accidental head impacts and repetitive headers on circulating miRNAs. | Acute | Prospective cohort study | Field | Soccer | Male premier league players, aged 18-35 [heading exercise n=47; head impacts during a match n=35 (non-concussive n=21)] | Male premier league players, aged 18-35 (high intensity exercise, n=47) | Heading exercise, head impacts (some concussive) during match play | Serum miRNAs | Pre season (match), baseline (exercise), 1 and 12 h post (both) | Six miRNAs (miR16-5-p, miR-18a-5p, miR-20a-5p, miR-93-5p, miR-107 and miR-130b-3p) were deregulated after non-concussive head impacts and following heading exercise (miR-24-3p, miR-27a-3p, miR-122-5p, miR-150-5p, miR-499a-5p and miR-885-5p). Repetitive headers target genes were linked to TGF-β signalling pathway. |
| Shahim et al. 2017^112^ | Evaluate whether serum NfL is a sensitive biomarker to detect subtle brain injury or mTBI in contact sports athletes. | Acute & chronic | Prospective cohort study | Field | Boxing | 14 (11 M, 3 F) amateur boxers, median age (IQR) 21.5 (20-26) | 14 healthy nonathletic controls, 23.5 (23-26); 12 gymnasts, 19 (18-22) | Boxing bout | Serum NfL | 7-10 days post and after 3 months of rest | NfL was increased in boxers 7-10 days post compared to after 3 months of rest and controls. NfL decreased after 3 months of rest, but was still higher than in controls. Multiple hits to the head caused higher concentrations of NfL. Serum NfL had a strong, positive correlation with correspondent CSF NfL . |
| Soriano et al. 2022^113^ | Assess alterations in the gut/saliva microbiome and blood biomarkers in college football players after a single concussion and during seasonal sports activity. | Semi-acute | Cohort study | Field | Am. football | 33 male collegiate players, aged 19.3 ± 1.4 | N/A | Am. football season (games and training) | Serum: NfL, GFAP, t-tau, UCH-L1, S100β, SAA; salivary and gut microbiome | Mid-season, post-season and off-season (after a rest period) | GFAP was significantly increased mid-season and post-season compared to off-season. No significant differences for S100β, SAA, NfL and salivary microbiome. Majority of tau and UCH-L1 samples were below quantification limit. |
| Stålnacke and Sojka 2008^56^ | Analyse if controlled heading of soccer balls elicits increased concentrations of S100B (compared to a no heading control group). | Acute | Randomised controlled trial | Lab | Soccer | 10 male amateur players, aged 22 ± 8 (age for entire sample n=19) | 9 male amateur players | 5 headers (ball dropped from 18 m, ball velocity 63.6 km/h) | Serum S100B | Before and 0.5, 2 and 4 h post | No significant findings. |
| Stålnacke et al. 2003^114^ | Investigate changes in biomarkers after a competitive ice hokcey game in relation to head impact metrics and compare with changes in elite basketball players after a game. | Acute | Cohort study | Field | Ice hockey | 26 male elite ice hockey players, aged 28 ± 4 | 18 elite basketball players, aged 25 ± 4 | Ice hockey game (body checkings, falls, collisions, boardings); basketball game (jumps, collisions, falls) | Serum: S100B, NSE | 1-2 h before and ≤1 h post | S100B increased significantly after both games, whereas NSE did not. Jumps in basketball correlated significantly with changes in S100B. |
| Stålnacke et al. 2004^115^ | Analyse serum concentrations of two biochemical markers of brain tissue damage in connection to a soccer game. | Acute | Cohort study | Field | Soccer | 28 male elite players, aged 26 ± 5 | N/A | Headers, jumps, falls and collisions during a competitive soccer game | Serum: S100B, NSE | 1-5 h before and immediately post | S100B and NSE levels were significantly higher after the game. Changes in S100B correlated with impacts, whereas NSE did not. |
| Stålnacke et al. 2006^116^ | Assess concentrations of brain damage markers following a competitive soccer game with respect to head trauma events. | Acute | Cohort study | Field | Soccer | 44 female elite players, aged 23 ± 3 | N/A | Headers, jumps, falls and collisions during a competitive soccer game | Serum: S100B, NSE | Before and immediately post | S100B and NSE increased significantly after the game. Changes in S100B correlated significantly with impacts, whereas NSE did not. |
| Stern et al. 2016^117^ | Examine tau-positive exosomes in plasma as a potential CTE biomarker in former Am. football players. | Chronic | Case-control study | Other | Am. football | 78 male symptomatic former NFL players, aged 54.5 ± 8.0 | 16 male asymptomatic non-contact sport athletes, 56.9 ± 7.2 | Am. football participation | Plasma exosomal tau | N/A | No differences in total plasma exosomes, footballers had significantly higher exosomal tau than controls. |
| Straume-Naesheim et al. 2008^118^ | Compare levels of S100B after head trauma with the effect of heading, high-intensity exercise, and playing in a league match. | Acute | Prospective cohort study | Field | Soccer | Professional soccer players: heading exercise n=46, mean age 26.1; head impacts during a match n= 69, 28.1 | Professional soccer players: high intensity exercise n=48, 26.1; match control n=56, 26.2 | Heading exercise (90 min), head impacts (some concussive) during match play. Controls: 90 min exercise, match w/o head trauma. | Serum S100B | Baseline, 1 and 12 h post | Serum S100B increased from baseline at 1 h post for all groups. |
| Symons et al. 2020^119^ | Investigate oculomotor performance, telomere length, and serum protein bio-markers in male and female Au. footballers. | Chronic | Cross sectional study | Other | Au. football | 95 (69 M, aged 23.3 ± 0.4; 26 F, aged 23.2 ± 0.9) amateur players | 49 (28 M, aged 22.5 ± 0.4; 21 F, aged 23.1 ± 0.8) amateur basketball, tennis, cricket, track and field athletes | Au. football participation | Saliva: telomere length; serum: tau, p-tau, NfL, 4-HNE | Pre-season | Au. footballers had reduced telomere length and increased 4-HNE, tau, and p-tau levels. No significant differences in NfL levels. |
| Tanriverdi et al. 2007a^120^ | Describe a case of decreased libido and impotence 2 weeks after an intensive kickboxing bout. | Acute & semi-acute | Case report | Other | Kickboxing | 20-year-old male amateur kickboxer | N/A | Multiple punches to the face and head during a kickboxing bout. | Basal: fT3, fT4, TSH, PRL, C, FSH, LH, f-T, t-T, IGF-I; peak: GH (blood) | 2 weeks, 3 and 9 months post | 2 weeks: mild hyperprolactinemia and hypogonadotropic hypogonadism, GH response normal. 3 and 9 months post: hormone levels normalised. |
| Tanriverdi et al. 2007b^121^ | Investigate the pituitary function in both retired and active amateur kickboxers. | Chronic | Cross sectional study | Other | Kickboxing | 22 (16 M, 6 F) amateur kickboxers (16 active, 6 retired), aged 27.3 ± 7.1 | 22 (17 M, 5 F) healthy non-boxing controls, aged 26.1 ± 5.4 | Kickboxing participation (years, number of bouts) | Basal: fT3, fT4, TSH, PRL, C, FSH, LH, t-T, IGF-I, GH; peak: GH, C (serum) | N/A | Basal hormone levels were in normal range. IGF-I was significantly lower in kickboxers than controls. IGF-I correlated negatively with age, duration of sports and number of bouts. 22.7% and 9.1% kickboxers had GH had ACTH deficiency, respectively. |
| Tanriverdi et al. 2008^122^ | Investigate the pituitary function in retired and active amateur boxers. | Chronic | Observational cross-sectional study | Other | Boxing | 61 male amateur, elite boxers (44 active, 17 retired), mean age (range) 26 (17-53) | N/A | Boxing (years, number of bouts) | Basal: fT3, fT4, TSH, PRL, C, FSH, LH, f-T, t-T, ACTH, IGF-I, GH; peak: GH, C (serum) | N/A | 18% of the boxers had a pituitary dysfunction (15% had GH and 8% had ACTH deficiency). |
| Tanriverdi et al. 2010^123^ | Investigate the presence of antipituitary antibodies (APAs) and antihypothalamus antibodies (AHAs) in amateur boxers. | Chronic | Cross sectional study | Other | Boxing | 61 male amateur, elite boxers (44 active, 17 retired), mean age (range) 26 (17–53) | 60 male non-contact sport healthy controls, mean age (range) 25 (18–50) | Boxing (years, number of bouts) | Serum: AHA, APA | N/A | AHAs and APAs detected in boxers (21.3% and 22.9%, respectively) but not in controls. |
| Vike et al. 2022^124^ | Identify metabolite alterations across a collegiate American football season. | Semi-acute | Cohort study | Field | Am. football | 23 male collegiate footballers, aged 21 ± 1 | N/A | Am. football season (training and matches) | Metabolites (serum) | Pre and post season | 200 metabolites changed from pre to post season; 64 of the 200 metabolites were associated with head acceleration events. |
| Wallace et al. 2018^125^ | Examine the effect of a bout of soccer heading on biomarkers (compared to peripheral impacts and no impacts). | Acute | Prospective controlled cohort study | Lab | Soccer | 11 male collegiate players, aged 23.7 ± 3.9 | N/A | 40 headers; sham condition: contact with ball using hands, chest or thigh | Serum NfL, plasma tau | Immediately before, 1 h and 3 weeks post | NfL increased significantly immediately following heading and sham condition and remained elevated 3 weeks post heading. Tau levels were unaffected. |
| Wirsching et al. 2018^126^ | Examine changes in plasma NfL levels following 10 controlled soccer headers (compared to a kicking control group). | Acute | Randomised controlled trial | Lab | Soccer | 18 (7 M, 11 F) players, aged 20.3 ± 1.5 | 16 (6 M, 10 F) players, aged 21.2 ± 1.4 | 10 soccer headers; controls: 10 kicks | Plasma NfL | Before and 0, 2 and 24 h post | Significant increase at 24 h post compared to baseline and controls. |
| Zetterberg et al. 2006^58^ | Determine if amateur boxing and severity of hits are associated with elevated levels of biochemical markers for neuronal injury. | Acute & chronic | Longitudinal cohort study | Field | Boxing | 14 (11 M, 3 F) amateur boxers, aged 22 ± 3.8 | 10 male nonathletic controls, aged 30 ± 6.3 | Boxing bout | CSF: NfL, t-tau, p-tau, GFAP, Aβ1-40, Aβ1-42, albumin | 7-10 days post and after 3 months of rest | NfL, t-tau, and GFAP were increased after a bout compared with after 3 months of rest. Increases were higher among boxers who received many or high-impact hits to the head. NfL and GFAP, but not t-tau, were significantly elevated after a bout compared with controls. At 3 months NfL remained eleveted compared to controls. No significant findings in other markers. |
| Zetterberg et al. 2007^127^ | Determine if standardised headings in soccer are associated with increased levels of biochemical markers for neuronal injury. | Acute | Non-randomised experimental study | Lab | Soccer | 23 male amateur soccer players, median age (range): 10 headers n=10, 26 (19-32); 20 headers n=13, 23 (20-28) | 9 male non athletes, median age (range) 24 (22-27) | 10 or 20 standing headers from a corner kick (kicked from 30 m) | CSF: NfL, t-tau, GFAP, S100B, albumin; serum: S100B, albumin | 7-10 days post | No significant findings. |
| Zetterberg et al. 2009^128^ | Assess the potential of a serum biomarkers to identify chronic neuronal injury in amateur boxers as compared to healthy controls w/o history of head trauma. | Chronic | Observational case-control study | Other | Boxing | 44 male amateur boxers, median age (range) 19 (17-28) | 23 healthy males w/o contact sport history, median age (range) 28 (19-50) | Boxing participation (boxing debut, years boxing, number of bouts) | Serum: S100B, NSE, BDNF, GFAP, H-FABP | After a 2-month period of nonparticipation in boxing | Boxers had higher levels of NSE than controls. NSE levels did not correlate with age, age at boxing debut, boxing duration or total number of bouts. No differences in other markers between groups (GFAP not detectable). |
| Zonner et al. 2019^129^ | Investigate if S100B elevations are associated with frequency and magnitude of subconcussive head impacts. | Acute & semi-acute | Longitudinal prospective cohort study | Field | Am. football | 15 high school footballers, aged 16.4 ± 0.5 | N/A | Am. football games and season | Serum S100B | Semi acute: pre and post season; acute: 4-5 h before and ≤1 h after 5 games | Acute: significant findings; changes in S100B were associated with impact metrics. Semi-acute: no significant findings. |

Am. football – American football; Au. football – Australian rules football; CHII – cumulative head impact index; MMA – mixed martial arts; NCAA – National Collegiate Athletic Association; NFL – National Football League; Aβ – amyloid beta; Apo – apolipoprotein; APP – amyloid precursor protein; AHA – antihypothalamus antibodies; APA – antipituitary antibodies; BDNF – brain-derived neurotrophic factor; BLBP – brain lipid-binding protein; CCL11 – C-C motif chemokine ligand 11 also referred to as eotaxin-1; CCL2 – chemokine (C-C motif) ligand 2 also referred to as monocyte chemoattractant protein 1 (MCP-1); CD11b – integrin alpha M; CD81 – cluster of differentiation 81; CK-BB – creatine kinase brain specific isoenzyme; CLDN5 – claudin 5; CRP – C-reactive protein; EVs – extracellular vesicles; CNPase – 2',3'-cyclic nucleotide 3'-phosphodiesterase; eotaxin-3 – also known as CCL26; EAAT1 – excitatory amino acid transporter 1; GFAP – glial fibrillary acidic protein; GM-CSF – granulocyte-macrophage colony-stimulating factor; H-FABP – heart-type fatty acid binding protein; 3HK – 3-hydroxykynurenine; HMGB1 – high mobility group box protein 1; 4-HNE – 4-hydroxynonenal; IgA – immunoglobulin A; IFN-γ – interferon-gamma; IL – interleukin; IP-10 – interferon gamma-induced protein 10; KYN – kynurenine; KYNA – kynurenic acid; MAP2 – microtubule associated protein 2; MBP – myelin basic protein; MCP-4 – monocyte chemoattractant protein -4; MDC – macrophage-derived chemokine; MIP-1α, -1β – macrophage inflammatory proteins -1 alfa and -1 beta; Ng – neurogranin; NGF – nerve growth factor; NfL – neurofilament light; NSE – neuron-specific enolase; OMG – oligodendrocyte myelin glycoprotein; PEA15 – phosphoprotein enriched in astrocytes-15; PINCH – particularly interesting new cysteine-histidine-rich protein; pNFH – phosphorylated neurofilament heavy protein; PRDX-6 – peroxiredoxin-6; p-tau181 – tau phosphorylated at threonine 181; QUIN – quinolinic acid; S100B – S100 calcium binding protein beta; S100B Auto-Ab – S100B autoantibodies; SAA – serum amyloid A; sAPP – soluble amyloid precursor protein; SBDPs – spectrin breakdown products; SNAP25 – synaptosome-associated protein 25; sTERM2 – soluble triggering receptor expressed on myeloid cells 2; SYP – synaptophysin; TARC – thymus and activation-regulated chemokine; TNF-α, -β – tumor necrosis factor alfa and beta; UCH-L1 – ubiquitin carboxyl-terminal hydrolase L1; VEGF – vascular endothelial growth factor; VILIP-1 – visinin-like protein 1; vWF – von Willebrand factor

Hormones: ACTH – adrenocorticotropic hormone; C – cortisol; FSH – follicle-stimulating hormone; fT3 – free T3; fT4 – free T4; f-T – free testosterone; GH – growth hormone; IGF-I – insulin-like growth factor 1; LH – luteinizing hormone; PRL – prolactin; TSH – thyroid stimulating hormone; t-T – total testosterone; T– testosterone

†Also analysed but not detectable: GFAP, t-tau, IL-6, IL-18, IL-1β, IL-33, IL-17A, IL-12 p70, IL-23, transforming growth factor-alpha (TGF-α), interferon-gamma (IFN-γ)

Table S5 Definitions of subconcussive head impacts provided in the studies that are included in the scoping review

| **Citation** | **Definition** |
| --- | --- |
| Huibregtse et al. 2020a^130^ | "Exposure to subconcussive head impacts, or impacts to the cranium that do not result in clinical signs and symptoms of concussions [1, 2], has the potential to lead to long-term neurological consequences, including neurocognitive impairments [3] and chronic traumatic encephalopathy (CTE) [4, 5]." |
| Kawata 2016^78^ | "Subconcussion can be defined as a head impact resulting from a low magnitude of force that does not elicit clinical signs of concussion (Bailes, Petraglia, Omalu, Nauman, & Talavage, 2013; McCrory et al., 2013)." |
| Kawata et al. 2017^131^ | "Athletes are exposed to single and often repetitive head impacts during contact sports and these impacts may not necessarily elicit signs of a concussion.1,2 Nevertheless, these impacts may damage neural cell integrity as well as increase brain vulnerability to a concussion from a less severe impact.3-5 The vast majority of research has focused on a single impact that elicits a concussion, whereas many athletes are frequently exposed to subconcussive head impacts prior to the concussive blow.5,6" |
| Kawata et al. 2018a^80^ | "Head impacts or rapid acceleration-deceleration of the body or torso that cause the brain to "slosh" within the cranium commonly occur in collision or contact sports without signs and symptoms associated with clinical diagnosis of concussion.1 These milder forms of head injury are called subconcussion and accumulating evidence suggests that exposure to repeated sub-concussive head impacts may cause long-term damage.1-3" |
| Major et al. 2020^84^ | "Although much research has focused on concussion, a form of mild traumatic brain injury (mTBI) that typically results in the rapid onset of short-lived impairment in neurological function (4), there is growing evidence that sub-concussive impacts that do not result in overt neurological impairment may also contribute to chronic consequences, particularly if experienced repeatedly (5)." |
| Nowak et al. 2022^95^ | "However, subconcussive head impacts in sports, which are defined as hits to the head that do not cause overt concussion symptoms, have emerged as a major public health concern (McCrory et al., 2017)." |
| O'Keeffe et al. 2020^132^ | "Additionally, it must be recognized that there does not need to be any subjective clinical signs or symptoms for a brain injury to have occurred. In that regard, the nature of certain sports such as American football, rugby, and boxing are such that repetitive exposure of the head to what is termed subconcussive forces may lead to an accumulation of silent damage to distinct brain regions.6-8" |
| Oliver et al. 2017^101^ | "In football, the majority of those impacts occur in the absence of an acute concussive injury, are repetitive in nature, and are thus categorized as sub-concussive head trauma. Nonetheless, even in the absence of an acute concussive injury, quantifiable neurological damage can be observed when examined via advanced imaging techniques6-10 and fluid biomarker quantification.11-13 Repetitive sub-concussive impacts associated with a lifetime of sports participation may lead to neurological impairments such as chronic traumatic encephalopathy (CTE), which also can now be detected via advanced positron emission tomography imaging such as [F18] FDDNP.9,14,15" |
| Oliver et al. 2019^46^ | "Throughout the course of a season, American football athletes are routinely exposed to head impacts that vary in magnitude and frequency.9,11 Most of these incidents do not result in a concussion diagnosis and are thus known as subconcussive events."  "The degree of neurological injury caused by subconcussive head trauma is not easily detectible, and this injury does not induce readily identifiable clinical signs or symptoms." |
| Pin et al. 2021^51^ | "Rather, it is accepted that some individuals may sustain one seemingly benign impact and be diagnosed with a concussion whereas others who sustain repeated seemingly high energy impacts report no ill effects.4 Such asymptomatic impacts, heretofore referred to as "subconcussive impacts," are common in contact sports." |
| Puvenna et al. 2014^105^ | "Sub-CHIs have been defined as impacts that are not of a magnitude sufficient to cause a clinically diagnosable concussion yet sufficient to cause detectable changes [2,3]." |
| Rubin et al. 2019^109^ | "Milder forms of head impact that do not elicit signs or symptoms associated with concussion are termed subconcussion." |
| Soriano et al. 2022^113^ | "Additionally, athletes in SRC suffer from the cumulative effects of repetitive subconcussive impacts, which are defined as events similar to those giving rise to a concussion but involving insufficient impact forces or accelerations to produce symptoms associated with mTBI (Shuttleworth-Edwards et al., 2008)." |
| Wirsching et al. 2019^126^ | "Subconcussive head impact is defined as an impact to the head that does not trigger clinical symptoms of concussion." |
| Zonner et al. 2019^129^ | "Annually, approximately 2.5 million high school and college athletes engage in contact sports that frequently induce rapid acceleration-deceleration of the body and head. These forces, without eliciting outward clinical symptoms of concussion, are referred to as subconcussive impacts, and contact-sport athletes reportedly endure several hundreds to a thousand subconcussive impacts per season." |

Table S6 Linear and rotational acceleration from repetitive subconcussive head impacts measured using an accelerometer and a gyroscope

| **Citation** | **RSHI exposure** | **Number of impacts** | **Linear (g)** | **Rotational (rad/s^2^)** | **Biomarker(s)** | **Device details** |
| --- | --- | --- | --- | --- | --- | --- |
| Dorminy et al. 2015^69^ | Linear standing headers (speed: 30, 40 and 50 mph; distance: 60, 90 and 120 ft respectively) | 5 per setting (speed, distance) | Mean (SD) per impact:  30 mph 34.7 (6.1)  40 mph 49.2 (10.09)  50 mph 50.8 (7.68) | Not measured | Serum S100B | Accelerometer (model 35A, Endevco Corporation, San Juan Capistrano, CA, USA) placement: custom-fitted mouthpiece |
| Huibregtse et al. 2020a^76^ | Linear headers (speed: 25 mph, distance: 40 ft) | 10 | Mean (SD) per impact: 33.2 (6.8) | Mean (SD) per impact: 3600 (1400) | Plasma S100B | Accelerometer (SIM-G, Triax Technologies, Inc., Norwalk, Connecticut) placement: at occipital protuberance using custom headband |
| Huibregtse et al. 2020b^77^ | Linear headers (speed: 25 mph, distance: 40 ft) | 10 | Mean (SD) per impact: 33.5 (4.3) | Mean (SD) per impact: 3630 (780) | Plasma: CL11, CCL2, IL-10 | Accelerometer (SIM-G; Triax Technologies, Inc., Norwalk, Connecticut) placement: below occipital protuberance using custom headband |
| Joseph et al. 2019^21^ | American football season (games and practices) | Mean (SD) per player per season:  471.6 (69.4) | Mean per season (cumulative):  1.25 × 10^4^  HHI group; mean (SD) per impact: 114.7 (5.3)  Non-HHI group; mean (SD) per impact: 63.6 (10.5) | Mean per season (cumulative):  5.66 × 10^5^  HHI group; mean (SD) per impact: 5224.5 (260.1)  Non-HHI group: mean (SD) per impact:  2346.8 (166.2) | Serum: NfL, tau, GFAP, SBDPs, UCH-L1 | Riddell Head Impact Telemetry System (HITS; Simbex) embedded in helmet |
| Kawata 2016^78^ | American football practices | Sum of five practices, mean (SD) per person: 31.5 (23.5) | Sum of five practices, mean (SD) per person:  867.3 (663.1) | Sum of five practices, mean (SD) per person: 43,377 (32,725) | Plasma: 100B, PINCH | Vector mouth guard (i1 Biometrics, Inc., Kirkland, WA, USA); accelerometer (ADXL377, Analog Devices, Norwood, MA, USA); gyroscope (L3GD20H, ST Microelectrics, Geneva, Switzerland) |
| Kawata et al. 2017^79^ | American football (pre-season) practices | Sum of five practices, median (range) per person:  32.5 (1-96) | Sum of five practices, median (range) per person: 1003.9 (40.9-2590.4) | Sum of five practices, median (range) per person: 68,259 (1348-14,9283) | Plasma S100B | Vector mouthguard (i1 Biometrics, Inc., Kirkland, WA, USA); accelerometer (ADXL377, Analog Devices, Norwood, MA, USA); gyroscope (L3GD20H, ST Microelectrics, Geneva, Switzerland) |
| Kawata et al. 2018a^80^ | American football (pre-season) practices | Sum of four practices median (IQR) per person: 26 (38) | Sum of four practices median (IQR) per person: 817.83 (856.61) | Sum of four practices median (IQR) per person: 44,612.57 (46,177.05) | Plasma Tau | Vector mouthguard (i1 Biometrics, Inc., Kirkland, WA, USA); accelerometer (ADXL377; Analog Devices, Norwood, MA); gyroscope (L3GD20H; ST Microelectrics, Geneva, Switzerland) |
| Nowak et al. 2022^95^ | Linear headers  (speed: 25 mph, distance: 40 ft) | 10 | Per header†:  ADHD 30.9 ± 2.1; non-ADHD 31.2 ± 3.91 | Per header†:  ADHD 3500 ± 1100; non-ADHD 3200 ± 700 | Plasma NfL, tau, GFAP, UCH-L1 | Triaxial accelerometer (Triax Technologies, Inc.) |
| Pin et al. 2021 ^133^ | Contact sport (mix) games during a season | Cumulative number of impacts (entire sample):  females: 3244; males: 21,290 | Median (IQR):  females: 13.3 (11.5-20.5);  males: 17.8 (15.7-26.6) | Not measured | Saliva autoimmune targets of IgA (IgA reactivity) | Triaxial accelerometer (gForce Tracker [GFT], Markham, ON, Canada or xPatch sensors, X2 Biosystems, Seattle, WA) |
| Rubin et al. 2019^109^ | American football (pre-season training camp) practices | Median (IQR) per person (cumulative):  23 (8-36) | Median (IQR) per person (cumulative): 642 (148-1038) | Median (IQR) per person (cumulative): 39,423 (8066-61,774) | Plasma NfL | Vector mouthguard (i1 Biometrics, Inc., Kirkland, WA, USA); accelerometer (ADXL377, Analog Devices, Norwood, MA, USA); gyroscope (L3GD20H, ST Microelectrics, Geneva, Switzerland) |
| Wirsching et al. 2019^126^ | Linear headers (speed: 11.2 m/s, distance: 12.2 m) | 10 | Median (IQR) per impact:  31.8 (31.1-34.5) | Median (IQR) per impact:  3560 (2930-4040) | Plasma NfL | Accelerometer (SIM-G; Triax Technologies, Inc., Norwalk, CT) placement: below occipital protuberance inside headband |
| Zonner et al. 2019^129^ | American football season (practices and games) | Median (IQR) per season: 596 (361.5-981.5) | Median (IQR) per season: 11,907 (7644-21,014) | Median (IQR) per season: 1,202,758 (637,515-2,100,871) | Serum S100B | Vector mouthguard (i1 Biometrics, Inc., Kirkland, WA, USA); accelerometer (ADXL377, Analog Devices, Norwood, MA); gyroscope (L3GD20H, ST Microelectrics, Geneva, Switzerland) |

† Not specified whether data provided was mean with SD or SEM

Table S7 Biofluid marker table for hormonal studies

| **Hormones** |  |  |  |  |  |  |  |  |  |  |  |  |  |
| --- | --- | --- | --- | --- | --- | --- | --- | --- | --- | --- | --- | --- | --- |
| **Publication** | **Study type** | **Design** | **Setting** | **Sport** | **Athlete group** | **Control group** | **Exposure** | **Hormones** | **Source** | **Sample times** | **Findings** | **Bias** | **QA** |
| Akkurt et al. 2020^59^ | Chronic | Cross sectional study | Other | Soccer | 32 retired professional players, aged 43.4 ± 5.5 | 26 healthy sedentary males, aged 43.3 ± 6.4 | Soccer participation | Basal: fT3, fT4, TSH, PRL, FSH, LH, t-T, IGF-I, ACTH, insulin; peak: C, GH | Blood | N/A | No significant findings. | Moderate | B (4) |
| Kelestimur et al. 2004^82^ | Chronic | Cross sectional study | Other | Boxing | 11 male amateur boxers (3 active, 8 retired), aged 38.0 ± 3.6 (range: 18-55) | 7 healthy non-boxing males, aged 34.4 ± 3.2 (range: 20-52) | Boxing (years, number of bouts) | Basal: fT3, fT4, TSH, PRL, C, FSH, LH, f-T, t-T, IGF-I, GH  Peak: GH | Serum | N/A | GH: significant findings (45% deficicent).  IGF-I: significantly lower in boxers. | Moderate | C (2) |
| Kelly et al. 2014^83^ | Chronic | Cross sectional study | Other | Am. football | 68 male retired NFL players, aged 47.3 ± 10.2 (range: 30-65) | 30 male non-head-injured controls (used for defining BMI adjusted GHD cut points) | NFL career (years, number of games) | Basal: fT4, tT4, TSH, PRL, C, FSH, LH, f-T, t-T, ACTH, IGF-I  Peak: GH, C | Serum | N/A | Hormone deficiency in 23.5% athletes. | Low | B (3) |
| Obminski et al. 2009^96^ | Chronic | Cross sectional study | Field | Boxing | 15 amateur boxers:  11 winners, aged 21.4 ± 1.7  4 defeated, aged 21.5 ± 1.3 | N/A | 3-round boxing match; total life-time number of boxing matches | T, GH, C | Plasma, capillary sample from earlobe | 3 min post | No significant findings. | Serious | C (0) |
| Roser et al. 2017^108^ | Chronic | Cross sectional study | Other | Soccer | 15 male professional soccer players, aged 31.1 ± 9.6 (range: 21-50) | N/A | Soccer participation | Basal: fT3, fT4, TSH, C, FSH, LH, T, IGF-I, GH  Peak: GH, C | Blood | N/A | No significant findings. | Moderate | C (2) |
| Tanriverdi et al. 2007a^120^ | Acute & semi-acute | Case report | Other | Kickboxing | 20-year-old male amateur kickboxer | N/A | Multiple punches to the face and head during kickboxing bout | Basal: fT3, fT4, TSH, PRL, C, FSH, LH, f-T, t-T, IGF-I  Peak: GH | Blood | 2 weeks, 3 and 9 months post | 2 weeks: mild hyperprolacti-nemia, hypogonado-tropic hypogonadism  3 and 9 months: hormone levels normal | Serious | C (1) |
| Tanriverdi et al. 2007b^121^ | Chronic | Cross sectional study | Other | Kickboxing | 22 (16 M, 6 F) amateur kickboxers (16 active, 6 retired), aged 27.3 ± 7.1 | 22 (17 M, 5 F) healthy non-boxing controls, aged 26.1 ± 5.4 | Kickboxing participation (years, number of bouts) | Basal: fT3, fT4, TSH, PRL, C, FSH, LH, t-T, IGF-I, GH  Peak: GH, C | Serum | N/A | IGF-I: significant findings.  22.7% and 9.1% kickboxers had GH had ACTH deficiency, respectively. | Moderate | C (2) |
| Tanriverdi et al. 2008^122^ | Chronic | Observational cross-sectional study | Other | Boxing | 61 male amateur, elite boxers (44 active, 17 retired), mean age (range) 26 (17-53) | N/A | Boxing (years, number of bouts) | Basal: fT3, fT4, TSH, PRL, C, FSH, LH, f-T, t-T, ACTH, IGF-I, GH  Peak: GH, C | Serum | N/A | 18% boxers had pituitary dysfunction. | Moderate | C (2) |
| Tanriverdi et al. 2010^123^ | Chronic | Cross-sectional study | Other | Boxing | 61 male amateur, elite boxers (44 active, 17 retired), mean age (range) 26 (17–53) | 60 male non-contact sport healthy controls, mean age (range) 25 (18–50) | Boxing (years, number of bouts) | AHA, APA | Serum | N/A | AHAs and APAs detected in boxers (22.9%) but not in controls. | Moderate | C (2) |

ACTH – adrenocorticotropic hormone; AHA – anti-hypothalamus antibodies; APA – anti-pituitary antibodies; C – cortisol; FSH – follicle-stimulating hormone; fT3 – free triiodothyronine; fT4 – free thyroxine; f-T – free testosterone; GH – growth hormone; IGF-I – insulin-like growth factor 1; LH – luteinizing hormone; NFL – National Football League; PRL – prolactin; TSH – thyroid stimulating hormone; t-T – total testosterone; T– testosterone

# References

1. Alosco ML, Tripodis Y, Jarnagin J, et al. Repetitive head impact exposure and later-life plasma total tau in former National Football League players. *Alzheimer’s Dement Diagnosis, Assess Dis Monit*. 2017;7:33-40. doi:10.1016/j.dadm.2016.11.003

2. Alosco ML, Tripodis Y, Koerte IK, et al. Interactive Effects of Racial Identity and Repetitive Head Impacts on Cognitive Function, Structural MRI-Derived Volumetric Measures, and Cerebrospinal Fluid Tau and Aβ. *Front Hum Neurosci*. 2019;13(December):1-14. doi:10.3389/fnhum.2019.00440

3. Alosco ML, Mariani ML, Adler CH, et al. Developing methods to detect and diagnose chronic traumatic encephalopathy during life: rationale, design, and methodology for the DIAGNOSE CTE Research Project. *Alzheimers Res Ther*. 2021;13(1):136. doi:10.1186/s13195-021-00872-x

4. Alpay N. Effects of acute exercise on serum cytokine composition in elite boxers: Th1/Th2/Th17 balance. *Stud Ethno-Medicine*. 2016;10(1):1-5. doi:10.1080/09735070.2016.11905464

5. Asken BM, Bauer RM, Dekosky ST, et al. Article concussion basics II baseline serum biomarkers, head impact exposure, and clinical measures. *Neurology*. 2018;91(23):E2123-E2132. doi:10.1212/WNL.0000000000006616

6. Bazarian JJ, Zhu T, Zhong J, et al. Persistent, long-term cerebral white matter changes after sports-related repetitive head impacts. *PLoS One*. 2014;9(4):e94734. doi:10.1371/journal.pone.0094734

7. Begum G, Reddy R, Yakoub KM, Belli A, Davies DJ, Di Pietro V. Differential expression of circulating inflammatory proteins following sport-related traumatic brain injury. *Int J Mol Sci*. 2020;21(4). doi:10.3390/ijms21041216

8. Bernick C, Shan G, Zetterberg H, et al. Longitudinal change in regional brain volumes with exposure to repetitive head impacts. *Neurology*. 2020;94(3):e232-e240. doi:10.1212/WNL.0000000000008817

9. Bevilacqua ZW, Huibregtse ME, Kawata K. In Vivo Protocol of Controlled Subconcussive Head Impacts for the Validation of Field Study Data. *J Vis Exp*. 2019;(146). doi:10.3791/59381

10. Brayne CEG, Dow L, Calloway SP, Thompson RJ. Bood Creatine Kinase Isoenzyme BB In Boxers. *Lancet*. Published online 1982:1308-1309.

11. Casson IR, Viano DC, Haacke EM, Kou Z, LeStrange DG. Is There Chronic Brain Damage in Retired NFL Players? Neuroradiology, Neuropsychology, and Neurology Examinations of 45 Retired Players. *Sports Health*. 2014;6(5):384-395. doi:10.1177/1941738114540270

12. Chen Y, Herrold AA, Martinovich Z, et al. Brain Perfusion Mediates the Relationship Between miRNA Levels and Postural Control. *Cereb cortex Commun*. 2020;1(1):tgaa078. doi:10.1093/texcom/tgaa078

13. Daisy CC, Varinos S, Howell DR, et al. Proteomic Discovery of Noninvasive Biomarkers Associated With Sport-Related Concussions. *Neurology*. 2022;98(2):e186-e198. doi:10.1212/WNL.0000000000013001

14. Di Pietro V, Porto E, Ragusa M, et al. Salivary MicroRNAs: Diagnostic Markers of Mild Traumatic Brain Injury in Contact-Sport. *Front Mol Neurosci*. 2018;11(August):1-13. doi:10.3389/fnmol.2018.00290

15. Di Pietro V, O’Halloran P, Watson CN, et al. Unique diagnostic signatures of concussion in the saliva of male athletes: the Study of Concussion in Rugby Union through MicroRNAs (SCRUM). *Br J Sports Med*. 2021;55(24):1395-1404. doi:10.1136/bjsports-2020-103274

16. España LY, Lee RM, Ling JM, Jeromin A, Mayer AR, Meier TB. Serial Assessment of Gray Matter Abnormalities after Sport-Related Concussion. *J Neurotrauma*. 2017;34(22):3143-3152. doi:10.1089/neu.2017.5002

17. Gill J, Merchant-Borna K, Jeromin A, Livingston W, Bazarian J. Acute plasma tau relates to prolonged return to play after concussion. *Neurology*. 2017;88(6):595-602. doi:10.1212/WNL.0000000000003587

18. Hirad AA, Bazarian JJ, Merchant-Borna K, et al. A common neural signature of brain injury in concussion and subconcussion. *Sci Adv*. 2019;5(8):1-12. doi:10.1126/sciadv.aau3460

19. Huibregtse ME, Zonner SW, Ejima K, et al. Association between muscle damage and head impacts in high school American football. *Int J Sports Med*. 2020;41(1):36-43. doi:10.1055/a-1021-1735

20. Johnston RD, Gibson N V, Twist C, Gabbett TJ, MacNay SA, MacFarlane NG. Physiological responses to an intensified period of rugby league competition. *J strength Cond Res*. 2013;27(3):643-654. doi:10.1519/JSC.0b013e31825bb469

21. Joseph JR, Swallow JS, Willsey K, et al. Elevated markers of brain injury as a result of clinically asymptomatic high-acceleration head impacts in high-school football athletes. *J Neurosurg*. 2019;130(5):1642-1648. doi:10.3171/2017.12.JNS172386

22. National Library of Medicine (U.S.). *Effects of Soccer Heading on Ocular-Motor Function and Blood Biomarker*. ClinicalTrials.gov identifier: NCT03488381. Accessed October 13, 2020. https://clinicaltrials.gov/ct2/show/NCT03488381

23. National Library of Medicine (U.S.). *The Effects of Fish Oil Supplementation on the Brain Health of Collegiate Football Athletes*. ClinicalTrials.gov identifier: NCT04796207. Accessed March 30, 2022. https://clinicaltrials.gov/ct2/show/NCT04796207

24. National Library of Medicine (U.S.). *Physiological Effects of Soccer Heading.* ClinicalTrials.gov identifier: NCT04810130. Accessed March 30, 2022. https://clinicaltrials.gov/ct2/show/NCT04810130

25. National Library of Medicine (U.S.). *The Effect of Intermittent Fasting on Acute Subconcussive Head Impacts.* ClinicalTrials.gov identifier: NCT05236127. Accessed March 30, 2022. https://clinicaltrials.gov/ct2/show/NCT05236127

26. Kawata K, Steinfeldt JA, Huibregtse ME, et al. Association Between Proteomic Blood Biomarkers and DTI/NODDI Metrics in Adolescent Football Players: A Pilot Study. *Front Neurol*. 2020;11. doi:10.3389/fneur.2020.581781

27. Kilianski J, Peeters S, Debad J, et al. Plasma creatine kinase B correlates with injury severity and symptoms in professional boxers. *J Clin Neurosci*. 2017;45:100-104. doi:10.1016/j.jocn.2017.07.021

28. Kılıc Y, Cetin HN, Sumlu E, Pektas MB, Koca HB, Akar F. Effects of boxing matches on metabolic, hormonal, and inflammatory parameters in male elite boxers. *Med*. 2019;55(6):1-11. doi:10.3390/medicina55060288

29. Kochsiek J, O’Donnell LJ, Zhang F, et al. Exposure to Repetitive Head Impacts Is Associated With Corpus Callosum Microstructure and Plasma Total Tau in Former Professional American Football Players. *J Magn Reson Imaging*. 2021;54(6):1819-1829. doi:10.1002/jmri.27774

30. Koerte IK, Bahr R, Filipcik P, et al. REPIMPACT - a prospective longitudinal multisite study on the effects of repetitive head impacts in youth soccer. *Brain Imaging Behav*. 2022;16(1):492-502. doi:10.1007/s11682-021-00484-x

31. Kokjohn TA, Maarouf CL, Daugs ID, et al. Neurochemical profile of dementia pugilistica. *J Neurotrauma*. 2013;30(11):981-997. doi:10.1089/neu.2012.2699

32. Kozioł K, Zebrowski J, Betlej G, et al. Changes in γH2AX and H4K16ac levels are involved in the biochemical response to a competitive soccer match in adolescent players. *Sci Rep*. 2020;10(1):1-17. doi:10.1038/s41598-020-71436-6

33. Machan M, Tabor JB, Wang M, et al. The Impact of Concussion, Sport, and Time in Season on Saliva Telomere Length in Healthy Athletes. *Front Sport Act living*. 2022;4:816607. doi:10.3389/fspor.2022.816607

34. McCrea M, Broglio SP, McAllister TW, et al. Association of Blood Biomarkers with Acute Sport-Related Concussion in Collegiate Athletes: Findings from the NCAA and Department of Defense CARE Consortium. *JAMA Netw Open*. 2020;3(1):1-16. doi:10.1001/jamanetworkopen.2019.19771

35. McDonald SJ, O’Brien WT, Symons GF, et al. Prolonged elevation of serum neurofilament light after concussion in male Australian football players. *Biomark Res*. 2021;9(1):4. doi:10.1186/s40364-020-00256-7

36. McLellan CP, Lovell DI, Gass GC. Biochemical and Endocrine Responses to Impact and Collision During Elite Rugby League Match Play. *J Strength Cond Res*. 2011;25(6). https://journals.lww.com/nsca-jscr/Fulltext/2011/06000/Biochemical_and_Endocrine_Responses_to_Impact_and.11.aspx

37. Meier TB, Nelson LD, Huber DL, Bazarian JJ, Hayes RL, McCrea MA. Prospective Assessment of Acute Blood Markers of Brain Injury in Sport-Related Concussion. *J Neurotrauma*. 2017;34(22):3134-3142. doi:10.1089/neu.2017.5046

38. Meier TB, Lancaster MA, Mayer AR, Teague TK, Savitz J. Abnormalities in functional connectivity in collegiate football athletes with and without a concussion history: Implications and role of neuroactive kynurenine pathway metabolites. *J Neurotrauma*. 2017;34(4):824-837. doi:10.1089/neu.2016.4599

39. Meier TB, Huber DL, Bohorquez-Montoya L, et al. A Prospective Study of Acute Blood-Based Biomarkers for Sport-Related Concussion. *Ann Neurol*. 2020;87(6):907-920. doi:10.1002/ana.25725

40. Meier TB, Nitta ME, Teague TK, Nelson LD, McCrea MA, Savitz J. Prospective study of the effects of sport-related concussion on serum kynurenine pathway metabolites. *Brain Behav Immun*. 2020;87(February):715-724. doi:10.1016/j.bbi.2020.03.002

41. Meier TB, España L, Nitta ME, et al. Positive association between serum quinolinic acid and functional connectivity following concussion. *Brain Behav Immun*. 2021;91:531-540. doi:10.1016/j.bbi.2020.11.011

42. Meier TB, Guedes VA, Smith EG, et al. Extracellular vesicle-associated cytokines in sport-related concussion. *Brain Behav Immun*. 2022;100:83-87. doi:10.1016/j.bbi.2021.11.015

43. Neselius S, Brisby H, Marcusson J, Zetterberg H, Blennow K, Karlsson T. Neurological assessment and its relationship to CSF biomarkers in amateur boxers. *PLoS One*. 2014;9(6):1-8. doi:10.1371/journal.pone.0099870

44. Nitta ME, Savitz J, Nelson LD, et al. Acute elevation of serum inflammatory markers predicts symptom recovery after concussion. *Neurology*. 2019;93(5):E497-E507. doi:10.1212/WNL.0000000000007864

45. Oliver JM, Jones MT, Kirk KM, et al. Effect of Docosahexaenoic Acid on a Biomarker of Head Trauma in American Football. *Med Sci Sports Exerc*. 2016;48(6):974‐982. doi:10.1249/MSS.0000000000000875

46. Oliver JM, Anzalone AJ, Stone JD, et al. Fluctuations in blood biomarkers of head trauma in NCAA football athletes over the course of a season. *J Neurosurg*. 2019;130(5):1655-1662. doi:10.3171/2017.12.JNS172035

47. Otto M, Holthusen S, Bahn E, et al. Boxing and running lead to a rise in serum levels of S-100B protein. *Int J Sports Med*. 2000;21(8):551-555. doi:10.1055/s-2000-8480

48. Owens TS, Calverley TA, Stacey BS, et al. Concussion history in rugby union players is associated with depressed cerebrovascular reactivity and cognition. *Scand J Med Sci Sports*. 2021;31(12):2291-2299. doi:10.1111/sms.14046

49. Oztasyonar Y. Interaction between different sports branches such as taekwondo, box, athletes and serum brain derived neurotrophic factor levels. *J Sports Med Phys Fitness*. 2017;57(4):457-460. doi:10.23736/S0022-4707.16.06070-X

50. Papa L, Johnson B, Walter AE, et al. Decreases in Dorsal Cervical Spinal Cord White Matter Tract Integrity Are Associated with Elevated Levels of Serum MicroRNA Biomarkers in NCAA Division I Collegiate Football Players. *Neurotrauma reports*. 2021;2(1):476-487. doi:10.1089/neur.2021.0036

51. Pin E, Petricoin EF, Cortes N, et al. Immunoglobulin A Autoreactivity toward Brain Enriched and Apoptosis-Regulating Proteins in Saliva of Athletes after Acute Concussion and Subconcussive Impacts. *J Neurotrauma*. 2021;38(17):2373-2383. doi:10.1089/neu.2020.7375

52. Rogatzki MJ, Morgan JE, Baker JS, Knox A, Serrador JM. Protein S100B and brain lipid-binding protein concentrations in the serum of recently concussed rugby players. *J Neurotrauma*. 2021;38(16):2247-2254. doi:10.1089/neu.2021.0004

53. Vike NL, Bari S, Stetsiv K, et al. A preliminary model of football-related neural stress that integrates metabolomics with transcriptomics and virtual reality. *iScience*. 2022;25(1). doi:10.1016/j.isci.2021.103483

54. Shahim P, Mattsson N, Macy EM, et al. Serum visinin-like protein-1 in concussed professional ice hockey players. *Brain Inj*. 2015;29(7-8):872-876. doi:10.3109/02699052.2015.1018324

55. Siman R, Shahim P, Tegner Y, Blennow K, Zetterberg H, Smith DH. Serum SNTF Increases in Concussed Professional Ice Hockey Players and Relates to the Severity of Postconcussion Symptoms. *J Neurotrauma*. 2015;32(17):1294-1300. doi:10.1089/neu.2014.3698

56. Stålnacke BM, Sojka P. Repeatedly heading a soccer ball does not increase serum levels of S-100B, a biochemical marker of brain tissue damage: An experimental Study. *Biomark Insights*. 2008;2008(3):87-91. doi:10.4137/bmi.s359

57. Tanriverdi F, Suer C, Yapislar H, et al. Growth hormone deficiency due to sports-related head trauma is associated with impaired cognitive performance in amateur boxers and kickboxers as revealed by P300 auditory event-related potentials. *Clin Endocrinol (Oxf)*. 2013;78(5):730-737. doi:10.1111/cen.12037

58. Zetterberg H, Hietala MA, Jonsson M, et al. Neurochemical aftermath of amateur boxing. *Arch Neurol*. 2006;63(9):1277-1280. doi:10.1001/archneur.63.9.1277

59. Akkurt S, Tanriverdi F, Kalay N, et al. Investigation of pituitary dysfunction in retired professional soccer players. *Rev Bras Med do Esporte*. 2020;26(6):503-507. doi:10.1590/1517-869220202606215617

60. Alosco ML, Tripodis Y, Jarnagin J, et al. Repetitive head impact exposure and later-life plasma total tau in former National Football League players. *Alzheimer’s Dement Diagnosis, Assess Dis Monit*. 2017;7(December):33-40. doi:10.1016/j.dadm.2016.11.003

61. Alosco ML, Tripodis Y, Fritts NG, et al. Cerebrospinal fluid tau, Aβ and sTREM2 in Former National Football League Players: Modeling the relationship between repetitive head impacts, microglial activation, and neurodegeneration. *Alzheimer’s Dement*. 2018;14(9):1159-1170. doi:10.1016/j.jalz.2018.05.004

62. Antonio J, Cabrera D, Knafo S, Thomas J, Peacock C, Tartar J. Neurofilament Light (NFL) in Division II Female Soccer Players: A Potential Biomarker for Brain Trauma. *J Exerc Physiol Online*. 2021;24(1):1-6. http://ezproxy.stir.ac.uk/login?url=https://search.ebscohost.com/login.aspx?direct=true&db=sph&AN=149262281&site=ehost-live

63. Arslan F, Büyükyazi G, Ulman C, Taneli F, Gözlükaya F, Çalkan M. Examining acute changes in some serum biochemical markers of brain tissue damage after free and Greco-Roman style wrestling. *Turkish J Biochem*. 2010;35(4):307-312.

64. Austin K, Lee BJ, Flood TR, et al. Serum neurofilament light concentration does not increase following exposure to low velocity football heading. *Sci Med Footb*. 2021;5(3):188-194. http://ezproxy.stir.ac.uk/login?url=https://search.ebscohost.com/login.aspx?direct=true&db=sph&AN=151190669&site=ehost-live

65. Bamaç B, Tamer GS, Colak T, et al. Effects of repeatedly heading a soccer ball on serum levels of two neurotrophic factors of brain tissue, BDNF and NGF, in professional soccer players. *Biol Sport*. 2011;28(3):177-181. doi:10.5604/959284

66. Bernick C, Zetterberg H, Shan G, Banks S, Blennow K. Longitudinal Performance of Plasma Neurofilament Light and Tau in Professional Fighters: The Professional Fighters Brain Health Study. *J Neurotrauma*. 2018;35(20):2351-2356. doi:10.1089/neu.2017.5553

67. Bouvier D, Duret T, Abbot M, et al. Utility of S100B Serum Level for the Determination of Concussion in Male Rugby Players. *Sport Med*. 2017;47(4):781-789. doi:10.1007/s40279-016-0579-9

68. Battista APD, Rhind SG, Richards D, Churchill N, Baker AJ, Hutchison MG. Altered blood biomarker profiles in athletes with a history of repetitive head impacts. *PLoS One*. 2016;11(7). doi:10.1371/journal.pone.0159929

69. Dorminy M, Hoogeveen A, Tierney RT, Higgins M, McDevitt JK, Kretzschmar J. Effect of soccer heading ball speed on S100B, sideline concussion assessments and head impact kinematics. *Brain Inj*. 2015;29(10):1158-1164. doi:10.3109/02699052.2015.1035324

70. Graham MR, Myers T, Evans P, et al. Direct hits to the head during amateur boxing is associated with a rise in serum biomarkers for brain injury. *Int J Immunopathol Pharmacol*. 2011;24(1):119-125. doi:10.1177/039463201102400114

71. Graham MR, Pates J, Davies B, et al. Should an increase in cerebral neurochemicals following head kicks in full contact karate influence return to play? *Int J Immunopathol Pharmacol*. 2015;28(4):539-546. doi:10.1177/0394632015577045

72. Heileson JL, Anzalone AJ, Carbuhn AF, et al. The effect of omega-3 fatty acids on a biomarker of head trauma in NCAA football athletes: a multi-site, non-randomized study. *J Int Soc Sports Nutr*. 2021;18(1):1-13. http://ezproxy.stir.ac.uk/login?url=https://search.ebscohost.com/login.aspx?direct=true&db=sph&AN=152654367&site=ehost-live

73. Hicks SD, Onks C, Kim RY, et al. Refinement of saliva microRNA biomarkers for sports-related concussion. *J Sport Heal Sci*. Published online August 28, 2021. doi:10.1016/j.jshs.2021.08.003

74. Hoffman JR, Ostfeld I, Zamir A, et al. Examination of Cognitive Function, Neurotrophin Concentrations, and both Brain and Systemic Inflammatory Markers Following a Simulated Game of American Football. *J Strength Cond Res (Lippincott Williams Wilkins)*. 2022;36(3):686-694. doi:10.1519/JSC.0000000000004218

75. Horner EB, Lee TC, Tipton KF, O’Brien M, Phillips JP. Creatine kinase and neuron-specific enolase: Serum markers of cell damage in the central nervous system in boxers. *Clin J Sport Med*. 1993;3(3):144-148. doi:10.1097/00042752-199307000-00002

76. Huibregtse ME, Nowak MK, Kim JE, et al. Does acute soccer heading cause an increase in plasma S100B? A randomized controlled trial. *PLoS One*. 2020;15(10 October):1-15. doi:10.1371/journal.pone.0239507

77. Huibregtse ME, Ejima K, Chen Z, Kalbfell RM, Koppineni A, Kawata K. Acute Time-Course Changes in CCL11, CCL2, and IL-10 Levels after Controlled Subconcussive Head Impacts: A Pilot Randomized Clinical Trial. *J Head Trauma Rehabil*. 2020;35(5):308-316. doi:10.1097/HTR.0000000000000597

78. Kawata K. Subconcussive head impact effect on plasma expression of S100-beta and PINCH proteins in collegiate football players. Published online 2016.

79. Kawata K, Rubin LH, Takahagi M, et al. Subconcussive Impact-Dependent Increase in Plasma S100β Levels in Collegiate Football Players. *J Neurotrauma*. 2017;34(14):2254-2260. doi:10.1089/neu.2016.4786

80. Kawata K, Rubin LH, Wesley L, et al. Acute Changes in Plasma Total Tau Levels Are Independent of Subconcussive Head Impacts in College Football Players. *J Neurotrauma*. 2018;35(2):260-266. doi:10.1089/neu.2017.5376

81. Kawata K, Mitsuhashi M, Aldret R. A preliminary report on brain-derived extracellular vesicle as novel blood biomarkers for sport-related concussions. *Front Neurol*. 2018;9(APR):1-11. doi:10.3389/fneur.2018.00239

82. Kelestimur F, Tanriverdi F, Atmaca H, KUnluhizarci, Selcuklu A, Casanueva FF. Boxing as a sport activity associated with isolated GH deficiency. *J Endocrinol Invest*. 2004;27(11). doi:10.1007/BF03345299

83. Kelly DF, Chaloner C, Evans D, et al. Prevalence of pituitary hormone dysfunction, metabolic syndrome, and impaired quality of life in retired professional football players: A prospective study. *J Neurotrauma*. 2014;31(13):1161-1171. doi:10.1089/neu.2013.3212

84. Major BP, McDonald SJ, O’Brien WT, et al. Serum Protein Biomarker Findings Reflective of Oxidative Stress and Vascular Abnormalities in Male, but Not Female, Collision Sport Athletes. *Front Neurol*. 2020;11. doi:10.3389/fneur.2020.549624

85. Marchi N, Bazarian JJ, Puvenna V, et al. Consequences of Repeated Blood-Brain Barrier Disruption in Football Players. *PLoS One*. 2013;8(3). doi:10.1371/journal.pone.0056805

86. Matuk R, Pereira M, Baird J, et al. The role of salivary vesicles as a potential inflammatory biomarker to detect traumatic brain injury in mixed martial artists. *Sci Rep*. 2021;11(1):8186. doi:10.1038/s41598-021-87180-4

87. Meier TB, Savitz J, Singh R, Teague TK, Bellgowan PSF. Smaller Dentate Gyrus and CA2 and CA3 Volumes Are Associated with Kynurenine Metabolites in Collegiate Football Athletes. *J Neurotrauma*. 2016;33(14):1349-1357. doi:10.1089/neu.2015.4118

88. Muñoz ER, Caccese JB, Wilson BE, et al. Effects of purposeful soccer heading on circulating small extracellular vesicle concentration and cargo. *J Sport Heal Sci*. 2021;10(2):122-130. doi:10.1016/j.jshs.2020.11.006

89. Muraoka S, Jedrychowski MP, Tatebe H, et al. Proteomic Profiling of Extracellular Vesicles Isolated From Cerebrospinal Fluid of Former National Football League Players at Risk for Chronic Traumatic Encephalopathy. *Front Neurosci*. 2019;13(October):1-12. doi:10.3389/fnins.2019.01059

90. Muraoka S, DeLeo AM, Yang Z, et al. Proteomic Profiling of Extracellular Vesicles Separated from Plasma of Former National Football League Players at Risk for Chronic Traumatic Encephalopathy. *Aging Dis*. 2021;12(6):1363-1375. doi:10.14336/AD.2020.0908

91. Mussack T, Dvorak J, Graf-Baumann T, Jochum M. Serum S-100B protein levels in young amateur soccer players after controlled heading and normal exercise. *Eur J Med Res*. 2003;8(10):457‐464. https://www.cochranelibrary.com/central/doi/10.1002/central/CN-00468959/full

92. Neselius S, Brisby H, Theodorsson A, Blennow K, Zetterberg H, Marcusson J. Csf-biomarkers in olympic boxing: Diagnosis and effects of repetitive head trauma. *PLoS One*. 2012;7(4):1-8. doi:10.1371/journal.pone.0033606

93. Neselius S, Zetterberg H, Blennow K, Marcusson J, Brisby H. Increased CSF levels of phosphorylated neurofilament heavy protein following bout in amateur boxers. *PLoS One*. 2013;8(11):1-5. doi:10.1371/journal.pone.0081249

94. Neselius S, Zetterberg H, Blennow K, et al. Olympic boxing is associated with elevated levels of the neuronal protein tau in plasma. *Brain Inj*. 2013;27(4):425-433. doi:10.3109/02699052.2012.750752

95. Nowak MK, Ejima K, Quinn PD, et al. ADHD May Associate With Reduced Tolerance to Acute Subconcussive Head Impacts: A Pilot Case-Control Intervention Study. *J Atten Disord*. 2022;26(1):125-139. doi:10.1177/1087054720969977

96. Obmiński Z, Hübner-Wožniak E, Stanisław Ł. Hormonal and Metabolic Blood Status in Boxers After a 3-Round Match. *Polish J Sport Tour*. 2009;16(4):221-224. http://ezproxy.newcastle.edu.au/login?url=http://search.ebscohost.com/login.aspx?direct=true&db=s3h&AN=47616281&login.asp&site=ehost-live&scope=site

97. O’Brien WT, Symons GF, Bain J, et al. Elevated Serum Interleukin-1β Levels in Male, but not Female, Collision Sport Athletes with a Concussion History. *J Neurotrauma*. 2021;38(10):1350-1357. doi:10.1089/neu.2020.7479

98. O’Connell B, Wilson F, Boyle N, et al. Effects of match play and training on circulating S100B concentration in professional rugby players. *Brain Inj*. 2018;32(13-14):1811-1816. doi:10.1080/02699052.2018.1532112

99. O’Keeffe E, Kelly E, Liu Y, et al. Dynamic Blood-Brain Barrier Regulation in Mild Traumatic Brain Injury. *J Neurotrauma*. 2020;37(2):347-356. doi:10.1089/neu.2019.6483

100. Oliver JM, Jones MT, Kirk KM, et al. Serum Neurofilament Light in American Football Athletes over the Course of a Season. *J Neurotrauma*. 2016;33(19):1784-1789. doi:10.1089/neu.2015.4295

101. Oliver JM, Jones MT, Anzalone AJ, et al. A Season of American Football Is Not Associated with Changes in Plasma Tau. *J Neurotrauma*. 2017;34(23):3295-3300. doi:10.1089/neu.2017.5064

102. Owens TS, Calverley TA, Stacey BS, et al. Contact events in rugby union and the link to reduced cognition: evidence for impaired redox-regulation of cerebrovascular function. *Exp Physiol*. 2021;106(9):1971-1980. doi:10.1113/EP089330

103. Papa L, Slobounov SM, Breiter HC, et al. Elevations in MicroRNA Biomarkers in Serum Are Associated with Measures of Concussion, Neurocognitive Function, and Subconcussive Trauma over a Single National Collegiate Athletic Association Division i Season in Collegiate Football Players. *J Neurotrauma*. 2019;36(8):1343-1351. doi:10.1089/neu.2018.6072

104. Pin E, Petricoin EF, Cortes N, et al. Immunoglobulin A Autoreactivity toward Brain Enriched and Apoptosis-Regulating Proteins in Saliva of Athletes after Acute Concussion and Subconcussive Impacts. *J Neurotrauma*. 2021;38(17):2373-2383. doi:10.1089/neu.2020.7375

105. Puvenna V, Brennan C, Shaw G, et al. Significance of ubiquitin carboxy-terminal hydrolase L1 elevations in athletes after sub-concussive head hits. *PLoS One*. 2014;9(5):1-9. doi:10.1371/journal.pone.0096296

106. Rogatzki MJ, Soja SE, McCabe CA, Breckenridge RE, White JL, Baker JS. Biomarkers of brain injury following an American football game: A pilot study. *Int J Immunopathol Pharmacol*. 2016;29(3):450-457. doi:10.1177/0394632016657091

107. Rogatzki MJ, Keuler SA, Harris AE, et al. Response of protein S100B to playing American football, lifting weights, and treadmill running. *Scand J Med Sci Sport*. 2018;28(12):2505-2514. doi:10.1111/sms.13297

108. Roser P, Wehrhahn T, Krogmann H, et al. Somatotrope Pituitary Function in Professional Soccer Players. *Exp Clin Endocrinol Diabetes*. 2018;126(5):306-308. doi:10.1055/s-0043-119876

109. Rubin LH, Tierney R, Kawata K, et al. NFL blood levels are moderated by subconcussive impacts in a cohort of college football players. *Brain Inj*. 2019;33(4):456-462. doi:10.1080/02699052.2019.1565895

110. Sandmo SB, Filipcik P, Cente M, et al. Neurofilament light and tau in serum after head-impact exposure in soccer. *Brain Inj*. 2020;34(5):602-609. doi:10.1080/02699052.2020.1725129

111. Sandmo SB, Matyasova K, Filipcik P, et al. Changes in circulating microRNAs following head impacts in soccer. *Brain Inj*. 2022;36(4):560-571. doi:10.1080/02699052.2022.2034042

112. Shahim P, Zetterberg H, Tegner Y, Blennow K. Serum neurofilament light as a biomarker for mild traumatic brain injury in contact sports. *Neurology*. 2017;88(19):1788-1794. doi:10.1212/WNL.0000000000003912

113. Soriano S, Curry K, Sadrameli SS, et al. Alterations to the gut microbiome after sport-related concussion in a collegiate football players cohort: A pilot study. *Brain, Behav Immun - Heal*. 2022;21:100438. doi:10.1016/j.bbih.2022.100438

114. Stålnacke BM, Tegner Y, Sojka P. Playing ice hockey and basketball increases serum levels of S-100B in elite players: A pilot study. *Clin J Sport Med*. 2003;13(5):292-302. doi:10.1097/00042752-200309000-00004

115. Stålnacke BM, Tegner Y, Sojka P. Playing soccer increases serum concentrations of the biochemical markers of brain damage S-100B and neuron-specific enolase in elite players: A pilot study. *Brain Inj*. 2004;18(9):899-909. doi:10.1080/02699050410001671865

116. Stålnacke BM, Ohlsson A, Tegner Y, Sojka P. Serum concentrations of two biochemical markers of brain tissue damage S-100B and neurone specific enolase are increased in elite female soccer players after a competitive game. *Br J Sports Med*. 2006;40(4):313-316. doi:10.1136/bjsm.2005.021584

117. Stern RA, Tripodis Y, Baugh CM, et al. Preliminary study of plasma exosomal tau as a potential biomarker for chronic traumatic encephalopathy. *J Alzheimer’s Dis*. 2016;51(4):1099-1109. doi:10.3233/JAD-151028

118. Straume-Naesheim TM, Andersen TE, Jochum M, Dvorak J, Bahr R. Minor head trauma in soccer and serum levels of S100B. *Neurosurgery*. 2008;62(6):1297‐305; discussion 1305‐6. doi:10.1227/01.neu.0000333301.34189.3d

119. Symons GF, Clough M, O’Brien WT, et al. Shortened telomeres and serum protein biomarker abnormalities in collision sport athletes regardless of concussion history and sex. *J Concussion*. 2020;4:205970022097560. doi:10.1177/2059700220975609

120. Tanriverdi F, Unluhizarci K, Selcuklu A, Casanueva FF, Kelestimur F. Transient hypogonadotropic hypogonadism in an amateur kickboxer after head trauma. *J Endocrinol Invest*. 2007;30(2):150-152. doi:10.1007/BF03347414

121. Tanriverdi F, Unluhizarci K, Coksevim B, Selcuklu A, Casanueva FF, Kelestimur F. Kickboxing sport as a new cause of traumatic brain injury-mediated hypopituitarism. *Clin Endocrinol (Oxf)*. 2007;66(3):360-366. doi:10.1111/j.1365-2265.2006.02737.x

122. Tanriverdi F, Unluhizarci K, Kocyigit I, et al. Brief communication: Pituitary volume and function in competing and retired male boxers. *Ann Intern Med*. 2008;148(11):827-831. doi:10.7326/0003-4819-148-11-200806030-00005

123. Tanriverdi F, De Bellis A, Battaglia M, et al. Investigation of antihypothalamus and antipituitary antibodies in amateur boxers: Is chronic repetitive head trauma-induced pituitary dysfunction associated with autoimmunity? *Eur J Endocrinol*. 2010;162(5):861-867. doi:10.1530/EJE-09-1024

124. Vike NL, Bari S, Stetsiv K, et al. Metabolomic response to collegiate football participation: Pre- and Post-season analysis. *Sci Rep*. 2022;12(1):3091. doi:10.1038/s41598-022-07079-6

125. Wallace C, Smirl JD, Zetterberg H, et al. Heading in soccer increases serum neurofilament light protein and SCAT3 symptom metrics. *BMJ Open Sport Exerc Med*. 2018;4(1):1-5. doi:10.1136/bmjsem-2018-000433

126. Wirsching A, Chen Z, Bevilacqua ZW, Huibregtse ME, Kawata K. Association of Acute Increase in Plasma Neurofilament Light with Repetitive Subconcussive Head Impacts: A Pilot Randomized Control Trial. *J Neurotrauma*. 2019;36(4):548-553. doi:10.1089/neu.2018.5836

127. Zetterberg H, Jonsson M, Rasulzada A, et al. No neurochemical evidence for brain injury caused by heading in soccer. *Br J Sports Med*. 2007;41(9):574-577. doi:10.1136/bjsm.2007.037143

128. Zetterberg H, Tanriverdi F, Unluhizarci K, Selcuklu A, Kelestimur F, Blennow K. Sustained release of neuron-specific enolase to serum in amateur boxers. *Brain Inj*. 2009;23(9):723-726. doi:10.1080/02699050903120399

129. Zonner SW, Ejima K, Bevilacqua ZW, et al. Association of increased serum S100B levels with high school football subconcussive head impacts. *Front Neurol*. 2019;10(APR):1-10. doi:10.3389/fneur.2019.00327

130. Huibregtse ME, Nowak MK, Kim JE, et al. Does acute soccer heading cause an increase in plasma S100B? A randomized controlled trial. Peyré-Tartaruga LA, ed. *PLoS One*. 2020;15(10):e0239507. doi:10.1371/journal.pone.0239507

131. Kawata K, Rubin LH, Takahagi M, et al. Subconcussive impact-dependent increase in plasma S100β levels in collegiate football players. *J Neurotrauma*. 2017;34(14):2254-2260. doi:10.1089/neu.2016.4786

132. O’Keeffe E, Kelly E, Liu Y, et al. Dynamic Blood-Brain Barrier Regulation in Mild Traumatic Brain Injury. *J Neurotrauma*. 2020;37(2):347-356. doi:10.1089/neu.2019.6483

133. Pin E, Petricoin E, Cortes N, et al. IgA autoreactivity towards brain enriched and apoptosis regulating proteins in saliva of athletes after acute concussion and subconcussive impacts. *J Neurotrauma*. Published online 2021. doi:10.1089/neu.2020.7375
